# Neurodevelopmental effects of methylmercury (MeHg): a review of epidemiological points of departure (PoDs), toxicological reference values (TRVs), and key uncertainties in human health risk assessment

**DOI:** 10.1007/s00204-026-04345-8

**Published:** 2026-03-10

**Authors:** Scott R. Blechinger, Kavita Singh, Abdul Afghan, Catherine A. Smith

**Affiliations:** 1 Bureau of Chemical Safety, Food and Nutrition Directorate, Health Canada, Ottawa, Canada; 2 Environmental Health Science and Research Bureau, Health Canada, Ottawa, Canada

**Keywords:** Methylmercury (MeHg), Neurodevelopment, Point of departure (PoD), Toxicological reference value (TRV), Epidemiology, Risk assessment

## Abstract

The adverse neurological effects of methylmercury are well characterized in animal models and humans. Neurodevelopmental effects in infants and children following prenatal exposure to methylmercury have been the critical endpoint used for many years in risk assessment for the derivation of toxicological reference values. Most risk assessments of methylmercury have continued to rely on the same epidemiological birth-cohorts from New Zealand, the Faroe Islands, and the Seychelles, in which neurodevelopmental effects of methylmercury were examined during the 1980s and 1990s. New epidemiological studies have since emerged, prompting several organizations to reassess whether current reference values remain protective. As these evaluations proceed, clarity on the basis for derivation of the existing values will be critical particularly if different toxicological reference values are recommended. This review summarizes the history, pivotal data, and methodologies used by different regulatory bodies and expert committees (US EPA, ATSDR, Health Canada, JECFA, and EFSA) to derive toxicological reference values for neurodevelopmental effects of prenatal methylmercury exposure. Although methodologies differed in the choice of points of departure, uncertainty factors, and dosimetry conversions, there is good agreement between the toxicological reference values derived by these organizations. Nonetheless, there remain important uncertainties and data gaps particularly related to how observational epidemiology data were used in derivation of the toxicological reference values. Discussion of these uncertainties should help inform future work on methylmercury and have broader implications for other chemical risk assessments relying on similar types of observational data.

**Supplementary Information:**

The online version contains supplementary material available at 10.1007/s00204-026-04345-8.

## Introduction

Methylmercury (MeHg) is a toxic form of mercury that is associated with adverse health outcomes in animals and humans. The most notable and well-established effects of MeHg are related to neurotoxicity and neurodevelopment and are considered the critical endpoints of concern (ATSDR 2024; EFSA 2012; NASEM 2024; US EPA 2001a). Early documented evidence of MeHg neurotoxicity in humans was based on historic case reports from occupational exposures (Ahlmark 1948; Hunter et al. 1940) and isolated small-scale poisoning incidents (Pierce et al. 1972). However, it was the widespread environmental MeHg contamination of fish and seafood in the Japanese regions of Minamata Bay and Niigata in the 1950s–1960s (Harada 1995) and a poisoning incident in Iraq (1971–1972) from the consumption of MeHg-treated seed grain (Bakir et al. 1973) that provided the earliest causal evidence of neurological effects in large samples of MeHg-exposed people. The major symptoms of MeHg neurotoxicity in adults observed during these incidents were paresthesia, ataxia, visual field constriction, dysarthria, hearing difficulties, and death in the most severe cases (Bakir et al. 1973; Harada 1995; Yorifuji et al. 2015).

Adverse neurodevelopmental effects were also reported in children whose mothers were exposed to MeHg during pregnancy. In Japan, severe effects in prenatally exposed children included profound intellectual disability, limb deformities, ataxia and other cerebral palsy-like symptoms; these effects were collectively classified as congenital Minamata Disease (CMD), of which up to 66 cases have been reported from the Japanese poisoning incidents (Harada 1995; Yorifuji et al. 2013). The lack of prenatal MeHg exposure data for the Japanese CMD cases made it difficult to establish a dose–response relationship for neurodevelopmental effects (FAO/WHO 1972; SEG 1971). A greater range of neurodevelopmental effects were recorded in the poisoning incident in Iraq, with severe symptoms reported at higher exposures (blindness, loss of hearing, seizures, spastic paralysis, severe cognitive and motor disability) and less severe symptoms reported at both lower and higher exposures (delays in developmental milestones and increased abnormal neurological scores) (Amin-Zaki et al. 1974). However, the initial analyses of the Iraqi poisoning data were based on a limited number of cases. In addition, there was significant uncertainty in the exposure biomarker since THg was only measured in maternal blood (MB) several months after delivery and therefore was not a reliable metric of prenatal MeHg exposure.

The earliest authoritative reviews of the health effects of MeHg focused on adult neurotoxicity as the critical endpoint because the Japanese and Iraqi poisoning incidents provided better data in adults to model dose–response relationships (FAO/WHO 1972; SEG 1971; WHO 1976) (see Supplemental File S1 for summaries of these reviews). While these early reviews raised the hypothesis of greater sensitivity of children exposed prenatally to MeHg, the available data at the time were insufficient to characterize the dose–response for childhood neurodevelopmental effects. Starting in the 1980s, an increasing number of epidemiological analyses of neurodevelopmental effects in children from the Iraq poisoning incident were published. These analyses used maternal hair (MH) THg during pregnancy, which was a more informative metric for prenatal MeHg exposure than MB THg sampled up to several months post-partum (Cox et al. 1989; Marsh et al. 1987). In addition, analyses on prospective birth-cohort data from New Zealand (Kjellström et al. 1989), the Faroe Islands Cohort 1 (Grandjean et al. 1997) and the Seychelles Pilot and Main Cohorts (Myers et al. 1995a, b) provided data for dose–response characterization of neurodevelopmental effects in children. These cohorts collected reliable THg biomarkers of prenatal exposure in MH, MB, or cord blood (CB), and the exposure distributions of these cohorts were much lower than the Japanese and Iraq poisonings, although generally above the range of mercury exposures for general populations in the United States (Wells et al. 2022) and Canada (Health Canada 2021). Several risk assessment organizations have used data from these cohorts to select points of departure (PoDs) and derive toxicological reference values (TRVs) for human health risk assessments of MeHg.

Hundreds of epidemiological studies on this topic have been published since the late 1990s including analyses from dozens of newer birth-cohorts (ATSDR 2024), however there remain multiple challenges when using these types of observational epidemiology data in chemical risk assessments (EFSA 2024; Phillips et al. 2024; Schaefer et al. 2025; WHO 2020). Risk assessment organizations are currently exploring whether the PoDs and/or TRVs for MeHg should be refined based on these newer data, as summarized in a recent review by the European Union (EU) HBM4EU Programme (Halldorsson et al. 2022), and ongoing activities by the US EPA (US EPA, n.d.-c). As refinements by these risk assessment organizations become available, a clear understanding of the derivation of these PoDs and TRVs will benefit risk assessors, policy makers, and others interested in MeHg.

The purpose of this review was to summarize the pivotal epidemiological data and methodologies supporting the current PoDs and TRVs for MeHg used by risk assessment organizations in Canada (Health Canada), the United States (US EPA, ATSDR), Europe (EFSA), and internationally (JECFA). In addition, a sensitivity analysis was conducted to derive supplementary PoDs based on (1) previously published meta-analyses of data from New Zealand, the Faroe Islands, and Seychelles cohorts (Axelrad et al. 2007; Coull et al. 2003), (2) a more recent meta-analyses including newer cohort data (Kopylev & Segal 2025), and (3) updated benchmark dose (BMD) modelling of adverse neurological scores in children from previously published Iraq poisoning data (Marsh et al. 1987). The supplementary PoDs from the sensitivity analyses showed close agreement with the range of PoDs currently used by risk assessment organizations, providing support for the continued use of the existing values for risk assessment. The key data gaps and uncertainties surrounding these values are also discussed, including the need for a quantitative synthesis of all eligible epidemiology data, uncertainties with deriving BMDs from reported linear regression slopes, and guidance on the choice of an appropriate benchmark response (BMR) for neurodevelopmental data. These data gaps and uncertainties have broader application to other chemical risk assessments that rely on similar types of observational epidemiology data.

### Terminology used in this review

**Fig. 1:**
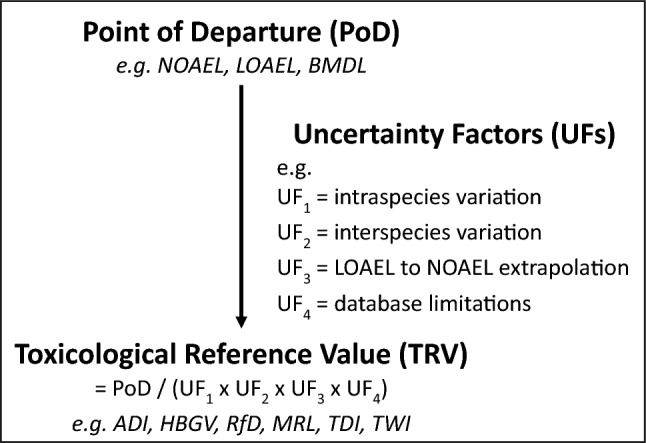
Terminology used in this review for point of departure (PoD) and toxicological references value (TRV)

For MeHg and other chemical hazards, regulatory and toxicology organizations select critical effect levels and derive corresponding acceptable/safe/tolerable exposure values which are subsequently used for risk assessment. While different terminologies are frequently employed for these values, this review used the following generic terms based on definitions from the United States Environmental Protection Agency (US EPA) Integrated Risk Information System (IRIS) Glossary (US EPA, n.d.-b) (https://www.epa.gov/iris/iris-glossary#tab1):

- **Point of Departure (PoD):** *“The dose–response point that marks the beginning of a low-dose extrapolation. This point can be the lower bound dose for an estimated incidence or a change in response level from a dose–response model (BMD), or a NOAEL or LOAEL for an observed incidence, or change in level of response”* (see Fig. 1).
- **Toxicological**^1^**Reference Value (TRV):** *“An estimate of an exposure for a given duration to the human population (including susceptible subgroups) that is likely to be without an appreciable risk of adverse health effects over a lifetime. It is derived from a BMDL (benchmark dose lower confidence limit), a NOAEL (no-obsevered-adverse-effect-level), a LOAEL (lowest-observed-adverse-effect-level), or another suitable point of departure, with uncertainty/variability factors applied to reflect limitations of the data used.”* Various terms are used by different groups to indicate a TRV, including ADI (allowable daily intake), HBGV (health-based guidance value), MRL (minimal risk level), RfD (reference dose), TDI or pTDI (tolerable daily intake, with p added for “*provisional*”, TDI and pTDI are used interchangeably), and TWI or pTWI (tolerable weekly intake, with p added for “*provisional*”, TWI and pTWI are used interchangeably) (see Fig. 1).

## PoDs and TRVs for neurodevelopmental effects of prenatal exposure to MeHg

In 1990, the World Health Organization (WHO) International Programme on Chemical Safety (IPCS) published the first review that attempted to quantify the effect level for neurodevelopmental effects in infants and children following prenatal MeHg exposure (WHO 1990). The WHO conducted a detailed evaluation of analyses from updated publications on the neurodevelopmental effects from the Iraq poisoning incident (Cox et al. 1989; Marsh et al. 1987), from frequent-fish eating populations including First Nations children in Quebec, Canada (McKeown-Eyssen et al. 1983), and a New Zealand birth-cohort (Kjellström et al. 1986, 1989). Based primarily on published dose–response data from the Iraq incident and supported by findings from the Canadian and New Zealand studies, the WHO report concluded:

*“The fetus is at particular risk. Recent evidence shows that at peak maternal hair mercury levels above 70 µg/g there is a high risk (more than 30%) of neurological disorder in the offspring. A prudent interpretation of the Iraqi data implies that a 5% risk may be associated with a peak mercury level of 10-20 µg/g in the maternal hair. There is a need for epidemiological studies on children exposed in utero to levels of methylmercury that result in peak maternal hair mercury levels below 20 µg/g, in order to screen for those effects only detectable by available psychological and behavioural tests.”* (WHO 1990).

While the 1990 WHO report did not formally establish a TRV for neurodevelopmental effects of MeHg, this work served as the foundational reference that informed later efforts by other risk assessment organizations. The earliest published TRV for neurodevelopmental effects was an oral reference dose (RfD) established in 1995 by the US EPA (US EPA, 1997b), and the most recently established TRV is a chronic minimum risk level (MRL) derived by the United States Agency for Toxic Substances and Disease Registry (ATSDR) in 2024 (ATSDR 2024). The following sections and Table 1 provide a brief history and explanation for the neurodevelopmental PoDs and TRVs for MeHg derived by the US EPA, the Joint FAO/WHO Expert Committee on Food Additives (JECFA), Health Canada (HC), the European Food Safety Authority (EFSA), and the ATSDR. The PoDs and TRVs described below and in Table 1 are not an exhaustive list, but rather a representative sample of values typically applied in risk assessments of MeHg.

**Table 1:** PoDs and TRVs for neurodevelopmental effects of prenatal exposure to MeHg used by various risk assessment organizations

	ATSDR	HC	JECFA	EFSA	US EPA
Critical Studies and Endpoints	PoD of 5.6 µg/g MH THg based on: 1. pooled β = − 0.18 IQ points per 1 µg/g in MH THg from a meta-analysis of βs from Faroe Islands Cohort 1, Seychelles Main Cohort, New Zealand conducted for the US EPA by Axelrad et al. (2007). The ATSDR used a critical effect size of − 1 IQ point to calculate an exposure of 5.6 µg/g MH THg used for the PoD (ATSDR 2024)	PoD “*approximate threshold*” of 10 µg/g MH THg based on: 1. Faroe Islands Cohort 1 at 7 years: dysfunction in language, attention, memory at < 10 µg/g MH THg (Grandjean et al. 1997) 2. WHO reported a PoD range of 10–20 µg/g MH THg based on the Iraq poisoning incident and New Zealand cohort (WHO 1990) 3. US EPA BMD modelling of Iraq poisoning data: BMDL_10_ of 11 µg/g MH THg for increased incidence of adverse effects (all outcomes) (US EPA 1995, 1997b) 4. Seychelles: adverse effects in the Pilot study at > 12 µg/g MH THg (Myers et al. 1995a, b) but no adverse effects observed in the Main Cohort (Myers et al. 1997)	PoD average of 14 µg/g MH THg based on: 1. Faroe Islands Cohort 1 at 7 years: BMDL_05_ of 12 µg/g MH THg (Budtz-Jørgensen et al. 2000; Budtz-Jørgensen et al. 1999a, b; NAS 2000) 2. Seychelles Main Cohort at 5.5 years: NOAEL of 15.3 µg/g MH THg (children of the highest exposed group ATSDR 1999; Davidson et al. 1998)	PoD average of 11.5 µg/g MH THg based on: 1. Faroe Islands Cohort 1 at 7 years: BMDL_05_ of 12 µg/g MH THg previously selected by JECFA (FAO/WHO 2004) 2. Seychelles Nutrition Cohort 1 at 9 and 30 months, adjusted for n-3 PUFAs: NOEL of 11 µg/g MH THg (Lynch et al. 2011)	PoD range of 46–79 µg/L CB THg based on: 1. Faroe Islands Cohort 1 at 7 years: BMD modeling performed on four outcome tests for the full cohort, and reduced sample adjusted for PCBs; BMDL_05_ values ranged from 46–79 µg/L CB THg as reported in the IRIS oral RfD summary (US EPA 2001a) and from Table 4–8 on p.4–61 of the US EPA report (US EPA 2001b) 2. Supporting BMD modelling outcomes from the New Zealand cohort at 6–7 years (Crump et al. 2000; NAS 2000) and Seychelles Main Cohort at 5.5 years (Crump et al. 2000) including an “*integrative analysis*” provided by the NAS (2000) (US EPA 2001b)
PoD_MH_ (µg/g, THg in maternal hair)	5.6 µg/g	10 µg/g	14 µg/g	11.5 µg/g	9–12 µg/g (measured)^a^ (11.5–20 µg/g converted from PoD_CB_ below)^a^
MH:CB ratio	N/A^b^	N/A^b^	N/A^b^	N/A^b^	0.250:1^c^
PoD_CB_ (µg/L, THg in cord blood)	N/A^b^	N/A^b^	N/A^b^	N/A^b^	46–79 µg/L^d^
MH(µg/g):MB(µg/L) ratio	0.203:1^f^	0.250:1^c^	0.250:1^c^	0.250:1^c^	N/A^e^
PoD_MB_ (µg/L, THg in maternal blood)	27.6 µg/L^f^	40 µg/L	56 µg/L	46 µg/L	46–79 µg/L^e^
Oral Intake of MeHg (conversion from MB) ^g^	oral intake of MeHg (µg/kg-bw/day) = [MB THg (µg/L)] *[(B*V)/(A*F*BW)] = [MB THg (µg/L)] * [CF]				
B (d^−1^, elimination rate constant)	0.0106	0.014	0.014	0.014	0.014
V (L, blood volume)	4.80	5	5.85	5.85	5
A (fraction oral absorption of MeHg) ^g^	0.94	0.95	0.95	0.95	0.95
F (fraction of A) partitioned to whole blood)	0.060	0.05	0.05	0.05	0.059
BW (kg, body weight)	60	60	65	65	67
CF (conversion factor) to convert MB to oral where: CF = (B*V)/(A*F*bw)	0.0150	0.0246	0.0265	0.0265	0.0186
PoD_oral_ (µg/kg-bw/day as MeHg)^g^	0.41 µg/kg-bw/day	1 µg/kg-bw/day (rounded from 0.984)	1.5 µg/kg-bw/day	1.2 µg/kg-bw/day	0.857–1.472 µg/kg-bw/day^h^
UF	3 (UF = 3 for human variability in TK and TD)	5 (UF = 5 to account for interindividual variability)	6.4 (UF = 2 for MH:MB ratio * UF = 3.2 for TK uncertainty)	6.4 (UF = 2 for MH:MB ratio * UF = 3.2 for TK uncertainty)	10 (UF = 3 for TK uncertainty including biomarker to oral conversions * UF = 3 for TD uncertainty)
TRV_oral_ (µg/kg-bw/day as MeHg)^g^	0.10 µg/kg/day^i^ (MRL)	0.20 µg/kg/day (pTDI)	0.23 µg/kg/day (pTDI equivalent to the reported pTWI of 1.6 µg/kg/week)	0.19 µg/kg/day (TDI equivalent to the reported TWI of 1.3 µg/kg/week)	≈ 0.1 µg/kg/day^j^ (RfD)
References	(ATSDR 2024)	(Feeley & Lo 1998; Health Canada 1997, 2007; Legrand et al. 2010)	(FAO/WHO 2004, 2007)	(EFSA 2012)	(US EPA 2001a, 2001b)

^a^PoD_MH_ range based on BMD modelling of measured MH THg was not explicitly selected by the US EPA since the US EPA chose CB over MH as the most appropriate biomarker for PoD selection (on p.4–64 of US EPA 2001b). The PoD_CB_ ranges from 46–79 µg/L CB THg (from US EPA 2001a and footnote ‘d’ in this Table) and is assumed to be based on the low–high range of candidate BMDL_05_ values (green highlighted cells in Table 2). The same candidate test scores correspond to a range BMDL_05_ values from 9–12 µg/g MH THg from measured MH THg and BMDL_05_ values from 11.5**–**20 µg/g MH THg converted from the PoD_CB_ range of 46–79 µg/L CB THg using a hair:blood ratio of 0.250:1 (see footnote ‘c’ in this Table)

^b^PoD_CB_ was not explicitly selected by these organizations, therefore no MH:CB ratio is shown

^c^More commonly reported as a ratio of 250:1 when the units are expressed as MH(µg/kg):blood(µg/L) where blood could be either CB or MB

^d^The US EPA PoD_CB_ ranges from 46–79 µg/L CB THg (from US EPA 2001a) and is assumed to be based on the low–high range of candidate BMDL_05_ values (green highlighted cells in Table 2). However, it should be noted that the specific PoD_CB_ range was not reported in the IRIS supporting document (i.e., US EPA Water Quality Criterion document; (US EPA 2001b)

^e^The US EPA PoD_MB_ was based on assumption of equivalent concentrations between CB and MB without conversion factor applied, therefore the PoD_MB_ = PoD_CB_ range from 46–79 µg/L CB THg (see footnote ‘d’ in this Table)

^f^The ATSDR did not explicitly report the PoD_MB_, however can be calculated with the 0.203:1 ratio used by the ATSDR (see Table A-26 on p.A-67 of ATSDR 2024). Note that this hair:blood ratio was most likely based on data for cord blood but was used by the ATSDR to apply to maternal blood

^g^The estimated oral intakes are for MeHg (not THg) and the oral absorption factor of 0.95 (95%) for ‘A’ reflects the much higher oral absorption of MeHg compared to inorganic Hg (EFSA 2012; ATSDR 2024). However, the reverse dosimetry oral intake calculation was based on measured THg in MB, despite the large majority expected to be in the form of MeHg (e.g., > 80%). Since all groups applied the same value of 0.95 for oral absorption (except ATSDR which used 0.94), it can be implied that all groups assumed 100% of MB was in the form of MeHg. Therefore, the PoD_oral_ and the corresponding TRV_oral_ for MeHg derived by all organizations in the table can be considered slightly overestimated (e.g., if MB is assumed to be 80% MeHg rather than 100%, then the PoD_oral_ and TRV_oral_ in this table could be multiplied by 0.80 to reasonably arrive at values that are 20% lower than shown in the table)

^h^The US EPA PoD_oral_ ranges from 0.857–1.472 µg/kg-bw/day MeHg (from US EPA 2001a) and is assumed to be based on the low–high range of candidate BMDL_05_ values (green highlighted cells in Table 2) as converted from the PoD_CB_ range from 46–79 µg/L CB THg (see footnote ‘d’ in this Table)

^i^The reported ATSDR oral MRL of 0.10 µg/kg-bw/day was rounded down from 0.137 (i.e., 0.41 µg/kg-bw/day ÷ UF3 = 0.137 µg/kg-bw/day)

^j^The US EPA “*calculated RfD values converge at the same point: 0.1 µg/kg/day*” from p.4–60 and in Table 4–8 on p.4–61 of the US EPA report (US EPA 2001b). Based on the range of PoD_oral_ of 0.857 to 1.472 µg/kg-bw/day MeHg and applying the UF of 10, the reported candidate oral RfDs would be 0.09 to 0.15 µg/kg-bw/day MeHg rounded to 2 decimals

This review does not provide an in-depth account of the epidemiological data underlying the PoDs and TRVs but instead focuses on the basis and assumptions for their derivation. A summary of the principal cohorts informing PoD selection (i.e., New Zealand, Faroe Islands Cohort 1, and Seychelles Main Cohort) is available in Supplemental File S2. For further details on cohort characteristics, assessed outcomes, and statistical approaches, readers are encouraged to consult the original publications on these studies. All pivotal epidemiological studies assumed the major maternal and prenatal mercury exposure was in the chemical form of MeHg, such as from contaminated seafood (Japanese poisoning), MeHg-treated grain (Iraq poisoning), diets high in fish (Seychelles, New Zealand, Quebec), or the consumption of marine mammals (pilot whale in Faroe Islands). However, the biomarkers of exposure in these studies were almost exclusively based on the measurement of THg in MH, MB, and CB. Since a high percentage of THg in these biomarkers is MeHg (80–90%), with strong correlation between THg and MeHg, it is generally considered that THg reasonably approximates MeHg exposure in these studies (EFSA 2012; US EPA 2001a).

### United States Environmental Protection Agency (US EPA)

#### 1995 RfD

In 1995, the US EPA lowered their chronic oral RfD to 0.1 µg/kg-bw/day based on neurodevelopmental effects in children, from a previous chronic oral RfD of 0.3 µg/kg-bw/day for neurologic effects in adults derived in 1985 (see Supplemental File S1). The details of the 1995 oral RfD derivation come from summaries in secondary publications by the US EPA, including the 1995 draft and 1997 final *Methylmercury Study Report to Congress* (MSRC) (US EPA 1995, 1997b) and the 2001 *Water Quality Criterion for the Protection of Human Health: Methylmercury* (US EPA 2001b). The updated RfD of 0.1 µg/kg-bw/day was based on neurodevelopmental effects in children exposed to MeHg prenatally during the Iraq poisoning incident (US EPA 1995, 1997b), using BMD modelling of outcome and exposure data from 81 Iraqi mother–child pairs, with peak MH THg as the measure of prenatal exposure (range: 1–674 µg/g MH THg) (Marsh et al. 1987). Neurodevelopmental outcomes in children included maternal reported outcomes (i.e., month first walking and talking, presence of “*mental*” symptoms and seizures) and a composite neurological score (0–11 with higher score for more abnormal) based on clinical examination by two neurologists who were blinded to study participants’ MeHg exposure status. For BMD modelling, the US EPA dichotomized outcomes as normal or abnormal,^2^ while exposure was modelled as a categorical variable using group means from 5 categories of peak MH THg (US EPA 1997b). The US EPA used a quantal Weibull model with benchmark response (BMR) of 10% extra risk considered as the most appropriate effect level for estimating the PoD. The BMD_10_ values ranged from 17.1–125.4 µg/g MH THg and the 95% lower confidence limit (BMDL_10_) ranged from 11.1–70.5 µg/g MH THg (Table 6–5 on p.6–28 of US EPA 1997b). The lowest (i.e., most conservative) estimates were derived from the incidence of all outcomes combined, with a BMD_10_ and BMDL_10_ of 17.1 and 11.1 µg/g MH THg, respectively; the latter was selected by the US EPA as their PoD. Sensitivity analyses were conducted to evaluate alternative BMR levels (including 1% and 5%), variations in exposure category groupings, and separate modelling of male and female children (Table 6–9 in US EPA 1997a, b). Despite these methodological variations, and the resulting estimates consistently supported a BMDL_10_ of 11 µg/g MH THg (US EPA 1997b).

The US EPA considered other dose–response data from the Iraq poisoning incident, a Seychelles Pilot study, Faroe Islands Cohort 1, and cohorts from New Zealand, Peru, Amazon, and First Nations groups in Canada. These additional dose–response data provided NOAELs, LOAELs, and “*thresholds*” that ranged from 5–26.7 µg/g MH THg (Table 6–12 in US EPA 1997b), demonstrating overlap and support for the US EPA’s selected BMDL_10_ of 11 µg/g. The US EPA converted the BMDL_10_ of 11 µg/g MH THg to 44 µg/L MB THg using a MH:MB ratio of 0.250:1 and then to an oral intake of 1.1 µg/kg-bw/day MeHg using a single-compartment toxicokinetic (TK) model,^3^ which was first used by the WHO (1990) and shown in Table 1. Applying a composite uncertainty factor (UF) of 10 resulted in an oral RfD of 0.1 µg/kg-bw/day MeHg (US EPA 1997b). This rationale formed the basis of the US EPA’s oral RfD until it was updated in 2001.

#### 2001 RfD

Following the establishment of the 1995 oral RfD of 0.1 µg/kg-bw/day, data became available from more recent prospective birth cohorts, prompting several initiatives to consider these new data. As part of a technical review of the 1995 draft MSRC (US EPA 1995), a subcommittee of the US EPA Science Advisory Board (SAB) recommended that the 1995 oral RfD, while based primarily on neurodevelopmental effects from the Iraq poisoning incident, continue to be retained until newer data from the Faroe Islands and Seychelles birth-cohorts became available and could be critically reviewed (US EPA 1997a). In 1998, a US inter-agency workshop on "*Scientific Issues Relevant to Assessment of Health Effects from Exposure to Methylmercury*” chaired by the National Institute of Environmental Health Sciences (NIEHS) reviewed many of the newer cohort studies, and, in particular, considered data from the Faroe Islands and Seychelles to be “*credible and provide valuable insights into the potential health effects of methylmercury*” (NIEHS 1998).

In 1999, US Congress directed the US EPA to contract the National Academy of Sciences (NAS) to conduct an independent expert panel review of the available health effects data for MeHg and provide recommendations to the US EPA on selecting an appropriate oral RfD (NAS 2000). The NAS report recommended basing a new RfD primarily on BMD modelling of neurodevelopmental test scores from the Faroe Islands Cohort 1 (NAS 2000). The NAS recommendations were largely adopted by the US EPA in a 2000 draft RfD document (US EPA 2000a), which underwent external peer review (US EPA 2000b) and was later adopted in 2001 by IRIS (US EPA 2001a, 2001b). The sections below briefly summarize the basis for the 2001 oral RfD, citing information extracted primarily from the NAS report (NAS 2000) and US EPA IRIS supporting document (US EPA 2001b).

The NAS report highlighted several uncertainties with the neurodevelopmental Iraq poisoning data upon which the earlier 1995 oral RfD was based. Much of the criticism was focused on uncertainty related to the maternal-reported milestones (first month walking and talking) since there was a long delay between the time of prenatal exposure and when the children were assessed with an average age of 2.5 years, and exact birth dates not generally known. Another strong criticism of this data was uncertainty about how the 81 mother–child pairs were recruited, including the sampling frame, eligibility criteria, and potential for selection bias. Additional dose–response modelling of the Iraq data subsequently suggested a very large range in potential PoDs depending on the model and assumptions made (Cox et al. 1995; Crump et al. 1995). In addition, other than child sex, other important covariates, such as birth outcomes, maternal and socioeconomic factors, were not available for adjustment (NAS 2000).

In line with opinions from the earlier NIEHS workshop (NIEHS 1998), the NAS report recommended the US EPA to focus instead on more recently published results on cohorts from New Zealand (child age 6–7 years), the Faroe Islands (child age 6.8 years) and the Seychelles (child age 5.5 years) (NAS 2000). In particular, the NAS summarized BMD modelling on individual participant data for 16 outcome tests from these cohorts, which is shown in Table 2 and in Supplemental File S2 for more detail. It should be noted that the BMD estimates of 16 outcome tests reported in NAS (2000) comprise only 34.8% of the 46 total eligible outcome tests assessed in these three cohorts as shown in Table 3 (see Supplemental File S2 for details). The basis for the selection of only these 16 tests for BMD modelling was not explicitly reported (NAS 2000).

**Table 2:**
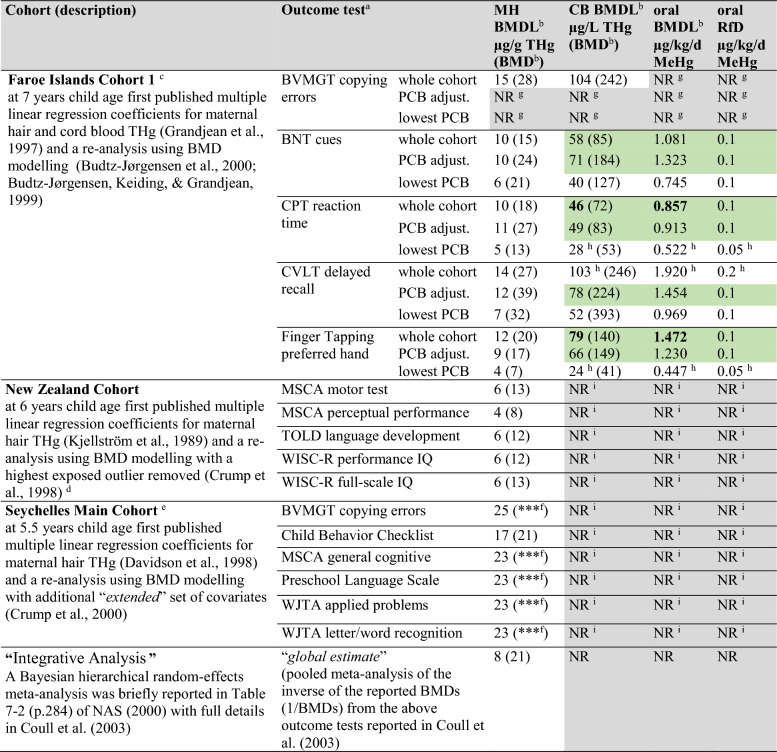
BMD modelling estimates for neurodevelopmental effects in cohorts from New Zealand, the Faroe Islands, and Seychelles (adapted from Table 7–2, Table 7–3, and Table 7–4 of NAS 2000; oral BMDL^b^ and RfD from Table 4–8 of US EPA 2001b)

Green highlighted cells indicate the candidate cord blood and oral BMDL^b^ values for the IRIS oral RfD and bolded values represent the reported PoD range in the IRIS summary (US EPA 2001a)

^a^BNT (Boston Naming Test), BVMGT (Bender Visual-Motor Gestalt Test), CPT (Continuous Performance Test), CVLT (California Verbal Language Test), MSCA (McCarthy Scales of Children’s Abilities), TOLD (Test of Language Development), WISC-R (Wechsler Intelligence Scale for Children-Revised), WJTA (Woodcock-Johnson Test of Achievement)

^b^Based on background abnormal P(0) = 5% and BMR = 5% added risk, the equivalent continuous outcome BMR =  ± 0.36 SDy (see Supp. File S3A)

^c^There are multiple birth-cohorts from the Faroe Islands exploring the association between neurodevelopment and prenatal MeHg exposure including Cohorts 1, 2, and 3 (Weihe & Joensen 2012)—see Supplemental File S2 for more information

^d^BMD modelling was originally reported using a P(0) = 5% and a BMR = 10% added risk = 0.61 SDy (Crump et al. 1998, 2000), but new BMD estimates using BMR = 5% added risk = 0.36 SDy were provided by Crump for the NAS report (see p.283 and Table 7–2 of NAS 2000)

^e^There are multiple birth-cohorts from the Seychelles exploring the association between neurodevelopment and prenatal MeHg exposure including the Pilot Study, Main Cohort, and Nutrition Cohorts 1 and 2 (van Wijngaarden et al. 2012)—see Supplemental File S2

^f^BMDs were *undefined* and reported as “***” (NAS 2000) as the regression coefficient was in the direction of benefit (Crump et al. 2000)

^g^NR (not reported): no BMD estimates or oral intakes were provided for PCB adjustment/strata for the BVMGT copying errors

^h^The US EPA reported these low- and high-end BMD estimates and their calculated RfDs as “*deviations*” (US EPA 2001b)

^i^NR (not reported): cord blood (CB) was not measured in New Zealand or Seychelles, BMDs and oral intakes were not reported

**Table 3:** Summary of relevant neurodevelopmental data from three early cohorts, including the total number of outcome tests assessed, reported regression coefficients (βs), coefficients included in the meta-analysis by Axelrad et al. (2007), and BMD estimates reported in NAS (2000)

Cohort information						Meta-analysis by Axelrad et al. (2007)		BMD modelling reported in NAS (2000)	
Cohort Name	Child age (years)	References where data are reported	Outcome tests assessed	Outcome tests with βs reported	Outcome tests with βs not reported	Outcome tests eligible for meta-analysis	βs Included in the meta-analysis	Outcome tests eligible for BMD modelling with individual participant data	BMD estimates reported
New Zealand	6–7	(Crump et al. 1998; Kjellström et al. 1989)	23	5	18	23	4	23	5
Faroe Islands Cohort 1	6.8	(Budtz-Jørgensen et al. 2000, 2005; Budtz-Jørgensen et al. 1999a, b; Grandjean et al. 1997)	17	17	0	17	6 (4*)	17	5
Seychelles Main Cohort	5.5	(Crump et al. 2000; Davidson et al. 1998)	6	6	0	Not Considered	Not Considered	6	6
	9	(Myers et al. 2003)	22	22	0	22	5	Not Considered	Not Considered
Total			68	50	18	62	15 (13*) (24.2% of eligible or 15/62)	46	16 (34.8% of eligible or 16/46)

*Three of six regression coefficients from the Faroe Islands Cohort 1 were used to create a single composite regression coefficient to represent WISC full-scale IQ, resulting in four coefficients from Faroe Islands Cohort 1, four from New Zealand, and five from Seychelles Main Cohort at 9 years for a total of 13 total coefficients used for pooling in the meta-analysis by Axelrad et al. (2007)

The BMD analyses reported in Table 2 were based on modelling of individual participant data on exposure (either MH or CB THg) and outcome test scores as a continuous variable, adjusted for covariates for each of the cohorts (Budtz-Jørgensen et al. 2000; Budtz-Jørgensen et al. 1999a, b; Crump et al. 1998, 2000). The BMD estimates were based on the K-power model (i.e., change in outcome test score = β*biomarker^K^), with K = 1 as the best fit which corresponds essentially to modelling a linear dose–response relationship. Since the outcome was modelled as a continuous variable, the “*hybrid approach*” method was used to determine a BMR as a change in the continuous outcome that was mathematically equivalent to the BMR risk of an abnormal score when modelled as a binary outcome (Crump 1995, 2002; Crump et al. 2000). Although the BMR as a continuous change used for the BMD modelling was not explicitly stated in the NAS report, based on previously described methods (Crump 1995, 2002; Crump et al. 2000) it can be inferred that a BMR of 0.36 standard deviations of the outcome score^4^ (0.36 SDy) was in fact used, which corresponds mathematically to the reported binary outcome BMR of 5% added risk (reported as “*excess risk*”) and a background probability of an “*abnormal*” score of P(0) = 5% (i.e., the lower 5th percentile of participant test scores for each outcome) (see Supplemental File S3A and S3B for description and example calculations for the BMR hybrid approach). Under this hybrid approach with a continuous change BMR = 0.36 SDy, the BMD and BMDL can be estimated directly from linear regression coefficient β and the lower limit on its 90% confidence interval (CI) or β_LCL90%CI_ as: BMD = BMR/ β and BMDL = BMR/ β_LCL90%CI_ as previously described (Coull et al. 2003; Grandjean et al. 2022) or by directly modelling the BMD after re-parameterizing the linear regression model to estimate the BMD rather than the coefficient β (Crump et al. 1998, 2000). Therefore, while both the NAS and US EPA reported the BMD model estimates using the binary outcome BMR definition as BMD_05_ and BMDL_05_ (NAS 2000; US EPA 2001b), it should be understood that the outcome test scores were all modelled as continuous variables with a continuous outcome BMR of 0.36 SDy, and therefore the reported BMD_05_ and BMDL_05_ estimates could also be equivalently reported as BMD_0.36SDy_ and BMDL_0.36SDy_ respectively (see Supplemental File S3A. and S3B).

Table 2 summarizes the key BMD and BMDL estimates extracted from the primary data and synthesized in reports by the NAS (2000) and US EPA (2001b). Additional BMD modelling was also provided for the Faroe Islands Cohort 1 outcomes adjusted for cord serum polychlorinated biphenyls (PCB) concentration as a continuous variable and by tertiles (lowest PCB tertile shown in Table 2). Pilot whale contains high levels of PCBs and may confound the association of mercury with outcomes, and therefore adjusting for PCBs in this cohort was important to more accurately model the association (NAS 2000). The bottom row of Table 2 also shows the results from an additional unpublished “*integrative analysis*” reported by the NAS that employed a hierarchical random-effects meta-analysis to arrive at an overall pooled estimate of the BMD and corresponding BMDL from all three cohorts combined (NAS 2000). This BMD meta-analysis was subsequently published in more detail by Coull et al. (2003); more details can be found in Sect. 4.2 of this review. Prenatal methylmercury exposure was measured as MH THg in all three cohorts, but CB THg was only measured in Faroe Islands Cohort 1. Therefore, BMD estimates for the New Zealand and Seychelles cohorts, originally expressed as MH THg, were converted to CB THg in the US EPA report using a hair:blood ratio of 0.250:1.^5^

Both the NAS (2000) and an external peer-review of the 2000 draft RfD (US EPA 2000b) recommended the use of the Faroe Islands Cohort 1 BMD modelling estimates as the basis for the PoD, but diverged in the choice of critical outcome test and BMDL_05_ for the derivation of the RfD. The NAS recommended a BMDL_05_ of 58 µg/L CB THg as the PoD based on Boston Naming Test (BNT) scores for the whole cohort (NAS 2000). The external peer-review instead recommended a BMDL_05_ of 71 µg/L CB THg as the PoD based on BNT scores adjusted for PCBs, but also proposed other alternative PoDs including based on a BMDL_05_ for the California Verbal Language Test (CVLT) delayed recall scores and a composite BMDL_05_ that integrated scores from multiple test instruments (US EPA 2000b). The external peer-review advised against using the Continuous Performance Test (CPT) reaction time scores since they noted several issues in data collection for this test instrument (US EPA 2000b). In consideration of the recommendations from the NAS and external peer-review, the US EPA compared different combinations of BMD modelling estimates, including a set of critical BMDL_05_ values from the Faroe Islands Cohort 1 as candidate PoDs for the derivation of the oral RfD (from Table 4–8 of the US EPA report and extracted in Table 2 in this review). While BMD estimates were available for both MH and CB THg, the US EPA stated “*…the best choice for exposure metric for RfD calculation is cord-blood mercury*” (p.4–64 of US EPA 2001b). The Faroe Islands Cohort 1 BMDL_05_ estimates in CB based on 4 outcome tests from the whole cohort adjusted for cord-blood PCBs, and the subset in the lowest PCB tertile, were assumed to be equivalent to BMDL_05_ estimates in MB.^6^ The BMDL_05_ estimates were first converted to BMDL_05_ oral equivalents based on a one-compartment TK model developed by the WHO (1990), and after application of a composite UF of 10, representing UFs of 3 each for TK and toxicodynamics (TD), the BMDL_05_ oral-equivalents were then expressed as candidate oral RfDs (Table 2).

The US EPA noted that the oral BMDL_05_ estimates ranged from 0.447–1.920 µg/kg-bw/day MeHg (Table 2) and after the application of a UF of 10 and rounding, “*the calculated RfD values converge at the same point: 0.1 µg/kg/day*” (p.4–60 of US EPA 2001b). The US EPA, however, noted a few “*deviations*” with two RfDs, one on the low end (0.05 µg/kg-bw/day based on CPT reaction time and Finger Tapping in the lowest PCB tertile) and one on the high end (0.2 µg/kg-bw/day based on CVLT delayed recall for the whole cohort), which were presumed to have been treated by the US EPA as outliers (Table 2). As stated in the US EPA report: “*Rather than choosing a single measure for the RfD critical endpoint, EPA considers that this RfD is based on several scores from the Faroes measures…The BMDLs for these scores are all within a relatively close range*” (p.4–60 of US EPA 2001b) and that “*The critical endpoint is drawn from the series of neuropsychological test results reported from the Faroese cohort*” (p.4–87 of US EPA 2001b).

While the 2001 RfD supporting document did not explicitly indicate which Faroe Islands outcome tests and corresponding BMDL_05_ values were the most critical (US EPA 2001b), the IRIS oral RfD summary (US EPA 2001a) reported the PoDs as a range of BMDL_05_ values from 46–79 µg/L CB THg and corresponding oral BMDL_05_ values from 0.857–1.472 µg/kg-bw/day MeHg. However, the IRIS summary did not provide an explicit justification for this particular range of BMDL_05_ values (US EPA 2001a). Based on Table 4–8 in US EPA 2001b and Table 2 in this review, these BMDL_05_ values match the CPT reaction time for the whole cohort on the low end (46 µg/L CB THg, 0.857 µg/kg-bw/day MeHg) and the Finger Tapping test for the whole cohort on the high end (79 µg/L CB THg, 1.472 µg/kg-bw/day MeHg) (Table 2). Since the BMDL_05_ range reported in IRIS omits several BMDL_05_ values listed in Table 2, it can be inferred that the US EPA applied selective exclusions when determining which values to report. These exclusions may have been influenced, at least in part, by the reported “*deviations*” in specific outcomes, including the lowest PCB tertile estimates for CPT reaction time and Finger Tapping, and the highest cohort-wide estimate for CVLT delayed recall, as described in the previous paragraph. If BMDL_05_ values associated with the lowest PCB tertile and CVLT delayed recall outcome for the full cohort are excluded as candidate BMDL_05_ values, the remaining CB BMDL_05_ values in Table 2 fall within a range of 46–79 µg/L CB THg, with corresponding oral BMDL_05_ values of 0.857–1.472 µg/kg-bw/day MeHg. This range aligns with the PoD range reported in the IRIS oral RfD summary (US EPA 2001a). While no specific PoD ranges were identified in the RfD supporting document (US EPA 2001b), this PoD range was reported in the IRIS oral RfD summary (US EPA 2001a), and is therefore assumed to represent the US EPA IRIS PoD range supporting the RfD (as presented in Tables 1–3). Notably, applying the same candidate PoD exclusions to the BMD modelling estimates for MH THg, yields a PoD range for maternal hair of 9–12 µg/g MH THg, as shown in Tables 2 and 3.

More recently, the US EPA IRIS program has initiated an update of the 2001 oral RfD that includes a systematic review of the published epidemiological data on neurodevelopmental effects of MeHg (US EPA 2020). As of January 2026, this work remains ongoing (US EPA, n.d.-c). A meta-analysis stemming in part from this systematic review was recently published, focusing specifically on estimates within the language/verbal subdomain (Kopylev & Segal 2025); however, these results have yet been integrated by the US EPA into a revised PoD.

### Axelrad et al. (2007) meta-analysis of regression coefficients from Faroe Islands, Seychelles, and New Zealand

While the 2001 IRIS RfD was based principally on PoDs selected from ranges of BMDL_05_ values from Faroe Islands Cohort 1, more complex methods to combine results from the Faroe Islands, New Zealand, and Seychelles were recommended by both the NAS expert panel (NAS 2000) and the external peer-reviewers for the 2000 draft RfD (US EPA 2000b). More sophisticated meta-analytical dose–response approaches for MeHg were subsequently developed to support the US EPA’s regulatory impact analyses related to reducing mercury air emissions (US EPA 2005, 2011). Based on earlier work by Ryan (2005), a publication by Axelrad et al. (2007) reported results of a Bayesian hierarchical meta-analysis of regression coefficients for outcomes test scores from Faroe Islands Cohort 1 at 7 years (Budtz-Jørgensen et al. 2005), New Zealand at 6–7 years (Kjellström et al. 1989), and Seychelles Main Cohort at 9 years (Crump et al. 2000; Myers et al. 2003). Of the 62 eligible regression coefficients identified across these studies, only 15 (24.2%) were included in the meta-analysis, as shown in Table 3 (see Supplemental File S2 for details). All of the regression coefficients used for pooling were from multiple linear regression models originally expressed as a change in unit of the outcome test score for an increase in the reported THg biomarker. The exposure increase for coefficients in the Faroe Islands Cohort 1 was an increase in 10 µg/L CB THg (Budtz-Jørgensen et al. 2005), which was standardized in Axelrad et al. (2007) as an increase in 1 µg/g MH THg to match the exposure increase reported for coefficients from New Zealand and Seychelles Main Cohort (see Supplemental File S2, Table S2.2 for details). Since the measured outcomes included various test instruments assessing different neurodevelopmental domains, including both cognitive and motor function which used different measurement scales, the coefficients were first standardized to a common unit of standard deviation (SD) of the outcome test score to facilitate pooling as a single neurodevelopmental score, and were then rescaled as a change in IQ score^7^ for easier interpretation (Axelrad et al. 2007).

The “*primary analyses*” resulted in a pooled, rescaled, multi-domain regression coefficient expressed as IQ score units of − 0.18 IQ points (SE = 0.092, σ_between-cohort_ = 0.112, 95% Bayesian Credible Interval: − 0.378, − 0.009) for an increase in 1 µg/g MH THg; see Table 5 of Axelrad et al. (2007). While the pooled regression coefficient is not a PoD per se, it has been used on several occasions for MeHg risk assessment,^8^ including for setting a mercury air quality standard (US EPA 2011), as the dose–response risk component of a quantitative risk–benefit analyses of seafood consumption (FAO/WHO 2011; US FDA 2014), and as a dose–response function for calculating global burden of disease for intellectual disability from prenatal MeHg exposure (Bellinger et al. 2019). The pooled regression coefficient from Axelrad et al. (2007) was also explicitly used by the ATSDR for derivation of a PoD in their final Toxicity Profile for Mercury (ATSDR 2024) (discussed in Sect. 2.3.2).

### United States Agency for Toxic Substances and Disease Registry (ATSDR)

#### 1999 MRL

In a review from 1999, the ATSDR summarized findings from the Seychelles Main Cohort at age 5.5 years (Davidson et al. 1998), which reported no adverse effects in multiple neurodevelopmental tests at exposures up to 15.3 µg/g MH THg (mean concentration in the highest exposed group) and an average exposure of 6.8 µg/g MH THg (ATSDR 1999). ATSDR considered 15.3 µg/g MH THg as the NOAEL from this study, which was converted to 61 µg/L MB THg using a hair:blood ratio of 0.250:1 and then to an oral intake of 1.3 µg/kg-bw/day MeHg using the one-compartment TK model from the WHO (1990). The ATSDR applied a composite UF of 4.5^9^ to derive a MRL of 0.3 µg/kg-bw/day MeHg (ATSDR 1999). In 2013, the ATSDR published an “*Addendum for Organic Mercury Compounds (Alkyl and Dialkyl Mercury Compounds)*” as a supplement to the 1999 Toxicity Profile (ATSDR 2013). The 2013 Addendum reviewed newer epidemiology studies on MeHg, including follow-up studies at older child ages from Faroe Islands Cohort 1 (Debes et al. 2006) and Seychelles Main Cohort (Myers et al. 2003), as well as cohorts from Oswego, New York State (Stewart et al. 2003), Poland (Jedrychowski et al. 2006), and Nunavik in Northern Quebec, Canada (Saint-Amour et al. 2006). However, the previous 1999 oral MRL was neither explicitly discussed nor updated in the 2013 Addendum (ATSDR 2013).

#### 2024 MRL

In October 2024, the ATSDR published a final Toxicology Profile for Mercury, which involved a comprehensive literature search and review of the available data on the neurodevelopmental effects of MeHg (ATSDR 2024). In addition to a robust evaluation of neurodevelopmental toxicity studies in mammalian models, this review summarized epidemiological results from more than 50 publications, including analyses of data on cohorts from the Faroe Islands, Seychelles, New Zealand, Nunavik, the Amazon basin, and others. The summary of epidemiology data included details such as child age at follow-up, type of biomarker specimen collected, average THg concentration in the biomarker, outcome tests administered, and overall result for each test categorized as showing either a positive, null, or inverse association with biomarker THg^10^ (see Tables and text on p.288–317 of ATSDR 2024). Based on this review the ATSDR concluded that “*Studies conducted in animals (nonhuman primates and rodents) and human epidemiological studies provide strong support for the developing nervous system being the most sensitive target of methylmercury*” (p.A-48 of ATSDR 2024).

From the large number of epidemiological studies reviewed, the ATSDR did not select a single pivotal endpoint, but rather chose the meta-analysis conducted by Axelrad et al. (2007) as the basis for their PoD, which they termed the NAEL (non-adverse effect level) (see p.A48 to A-70 of ATSDR 2024 and Sect. 2.2 for a description of the Axelrad et al. meta-analysis). The standardized coefficients were pooled using a Bayesian hierarchical meta-analysis to arrive at the primary pooled multi-domain regression coefficient of − 0.18 for an increase in 1 µg/g MH THg (Axelrad et al. 2007). Since this pooled estimate represented the slope for a linear relationship, the ATSDR applied what they defined as a critical change of − 1 IQ points to calculate a corresponding exposure increase of 5.6 µg/g MH THg,^11^ which was used as the NAEL or PoD_MH_ (p.A-65 to A-66 of ATSDR 2024). This NAEL of 5.6 µg/g MH THg was then converted to 27.6 µg/L MB THg based on a hair:blood ratio of 0.203:1^12^ and then to an oral intake of 0.41 µg/kg-bw/day using a one-compartment TK model (see Table 1). The TK model used by the ATSDR^13^ was cited as from Albert et al. (2010), but is the exact same model originally derived by the WHO (1990) to estimate oral intake of MeHg from MB THg. The ATSDR then applied a UF of 3 to account for human variability in TK and TD. The UF of 3 was reduced from a default UF of 10 since the studies included in the meta-analysis were based on the fetus (the most sensitive subpopulation) and there was reasonable confidence in the TK model to estimate maternal oral intake (p.A-70 of ATSDR 2024). After applying the UF of 3 to the oral NAEL of 0.41 µg/kg-bw/day, a chronic oral MRL of 0.1 µg/kg-bw/day^14^ was derived (ATSDR 2024).

### Joint FAO/WHO Expert Committee on Food Additives (JECFA)

The text below describes JECFA evaluations related to estimating the effect level for adverse neurodevelopment effects in children following prenatal MeHg exposure. See Supplemental File 1 for earlier JECFA evaluations from 1972 and 1976 (FAO/WHO 1972, 1978) that established PoDs and TRVs for adult neurotoxicity based on adult poisoning cases from Japan and Iraq.

#### 1989 Evaluation

At the 33rd JECFA meeting, additional data relevant to the neurodevelopmental effects of MeHg were considered (FAO/WHO 1989). Updated analyses of data on prenatally exposed children from the Iraq poisoning incident (Amin‐Zaki et al. 1981; Cox et al. 1989) indicated delays in maternal reported milestones (walking & talking) and a dose-dependent increase in adverse neurological signs in infants of mothers with peak gestational MH THg exposures ranging from 23–674 µg/g. Cox et al. (1989) suggesting a “*practical threshold*” or ELEL (estimated lowest effect level) of 10 µg/g THg, based on the intersection point of two linear fits from a “*hockey-stick*” dose–response model. In addition, follow-up data at 4 years of age from the New Zealand cohort suggested adverse effects on the Denver Development Screening Test (DDST) among children whose mothers had MH THg levels of 9–10 µg/g (Kjellström et al. 1986). In contrast, a study of prenatally exposed First Nations children from Quebec (Canada) reported inconclusive results at comparable exposure levels (McKeown-Eyssen et al. 1983). The JECFA committee reaffirmed the earlier derived TRV for the neurotoxicity of MeHg in adults (i.e., 300 µg/day MeHg expressed as a pTWI of 200 µg/week or 3.3 µg/kg-bw/week for a 60 kg adult—see Supplemental File S1), but indicated that this value was no longer applicable to the sensitive subgroup of pregnant women and nursing mothers. The JECFA committee stated that *“the available data were considered insufficient”* to determine a clear TRV for neurodevelopmental effects in this sensitive subgroup (FAO/WHO 1989).

#### 2000 evaluation

At the 53rd JECFA meeting (FAO/WHO 2000), the JECFA committee conducted a critical review of newer epidemiological studies including from: multiple updated analyses of neurodevelopmental data from the Iraq poisoning incident (Cox et al. 1995; Crump et al. 1995), a fishing community in Peru (Marsh et al. 1995), the New Zealand Cohort at 6 years (Kjellström et al. 1989), Faroe Islands Cohort 1 at 7 years (Grandjean et al. 1997, 1998), and Seychelles Pilot and Main Cohort data at 5.5 years (Axtell et al. 1998; Davidson et al. 1998). The JECFA committee noted substantial inconsistencies among these data, in particular the adverse findings from the Faroe Islands Cohort 1 and generally null-beneficial effects in the Seychelles Main Cohort, which were possibly attributable to differences in sources of MeHg exposure (pilot whale vs marine fish, respectively). The JECFA committee also noted uncertainty in the case series from the Iraq poisoning incident due to potential recall bias for some mother-reported outcomes. The JECFA committee acknowledged the PoD range of 10–20 µg/g MH THg previously proposed by the WHO IPCS (WHO 1990), but considered the collective data too inconsistent to arrive at definitive conclusions for maternal hair levels of ≤ 20 µg/g MH THg (FAO/WHO 2000) and the discussion was deferred to a future JECFA meeting.

#### 2004 Evaluation

At the 61st JECFA meeting (FAO/WHO 2004), additional neurodevelopmental studies were considered, including new results from the Seychelles Pilot study at 9 years (Davidson et al. 2000), the Seychelles Main Cohort at 5.5 years (Palumbo et al. 2000) and 9 years (Myers et al. 2003), re-analyses of the Seychelles Main Cohort at 5.5 years (Axtell et al. 2000; Davidson et al. 2001), re-analysis of the Faroe Islands Cohort 1 at 7 years (Budtz-Jørgensen et al. 1999a, b; Grandjean et al. 1999), data from the Faroe Islands Cohort 2 at 2 weeks of age (Steuerwald et al. 2000), and a study in Greenland Inuit children assessed at 9–12 years (Weihe et al. 2002). 

Despite the availability of new data from multiple cohorts, JECFA continued to focus their PoD selection on previous data from the Iraq poisoning incident,^15^ Faroe Islands Cohort 1 (NAS 2000), New Zealand (Crump et al. 1998), and Seychelles Pilot and Main Cohorts (Clewell 1998; Crump et al. 2000; Shipp et al. 2000). The critical PoDs were selected from BMD modelling estimates available for the Faroe Islands Cohort 1 at 7 years and Seychelles Main Cohort at 5.5 years (see Table 9 and Sect. 4.3, p.611–613 of FAO/WHO 2004). From Faroe Islands Cohort 1, the JECFA committee selected a single PoD of 12 µg/g MH THg from among various BMD estimates published by the original authors (Budtz-Jørgensen et al. 1999a, b; Steuerwald et al. 2000), and also reported in the NAS review of the US EPA (NAS 2000) and in the US EPA Water Quality Criterion document, in which the 2001 oral RfD was derived (see Table 9 and Sect. 4.3, p.611–613 of FAO/WHO 2004). These studies presented multiple BMDL_05_ values in both MH and CB THg, however it is unclear which specific outcome test score or BMD estimate corresponded to the PoD of 12 µg/g MH THg. The JECFA committee also selected a NOAEL of 15.3 µg/g MH THg for the Seychelles Main Cohort at 5.5 years (Davidson et al. 1998) based on the conclusion by the ATSDR that no adverse effects were observed in children in the highest exposure category of 15.3 µg/g MH THg (ATSDR 1999).

The JECFA committee derived an overall PoD of 14 µg/g MH THg from the average of the BMDL_05_ of 12 µg/g from Faroe Islands Cohort 1 and the NOAEL of 15.3 µg/g from Seychelles Main Cohort. The PoD_MH_ of 14 µg/g MH THg was then converted to a PoD_MB_ of 56 µg/L MB THg using a hair:blood ratio of 0.250:1,^16^ which was considered *“a concentration of mercury in maternal blood that would have no appreciable adverse effects on offspring in these two study populations”* (p.613 of FAO/WHO 2004). Oral intake was estimated from the PoD_MB_ using the same one-compartment TK model employed by the US EPA but with slight differences in inputs (Table 1) resulting in a PoD_oral_ of 1.5 µg/kg-bw/day MeHg. A composite UF of 6.4 was applied to account for TK variability due to the hair:blood ratio (UF of 2) and variability in estimating oral intake from MB (UF of 3.2) (i.e., composite UF = 2 * 3.2 = 6.4). The JECFA committee considered that an additional UF for human variability in toxicodynamics was not needed given the critical neurodevelopmental effect was for a sensitive subgroup and the pivotal studies were from diverse populations. The JECFA committee applied the composite UF of 6.4 to arrive at a provisional tolerable daily intake (pTDI) of 0.23 µg/kg-bw/day MeHg (Table 1), which was expressed by JECFA as a provisional tolerable weekly intake (pTWI) of 1.6 µg/kg-bw/week MeHg and considered “*sufficient to protect developing fetuses, the most sensitive subgroup of the population*” (FAO/WHO 2004).

#### 2007 Evaluation

At the 67th JECFA meeting, the JECFA committee considered additional studies not previously reviewed in their 2004 evaluation, including new data from Faroe Islands Cohort 1 at 14 years (Debes et al. 2006), as well as re-analyses from the Seychelles Main Cohort at 5.5 years (Davidson et al. 2004; Huang et al. 2003) and 9 years (Huang et al. 2005) (FAO/WHO, 2007). The JECFA committee concluded that these newer data supported previous findings of adverse and null effects in the Faroe Islands and Seychelles cohorts, respectively. Other cohort data not reviewed in previous JECFA evaluations were also considered, including higher-exposure studies from Inuit children in Nunavik (Després et al. 2005; Saint-Amour et al. 2006), as well as three studies from lower exposed populations from Krakow, Poland (Jedrychowski et al. 2006), the ALSPAC cohort in Southwest England (Daniels et al. 2004), and the Project Viva cohort from Boston, USA (Oken et al. 2005). However, no formal dose–response characterization, BMD analysis, nor critical synthesis of candidate PoDs from these newer studies was conducted. Rather, the JECFA committee considered that these new data supported the statement that “*the embryo and fetus [was] the most vulnerable stage of life with respect to adverse effects of MeHg*” and concluded that the previously derived pTWI of 1.6 µg/kg-bw/week MeHg (calculated from a pTDI of 0.23 µg/kg-bw/day MeHg) did not need to be revised (FAO/WHO 2007).

### Health Canada (HC)

In 1997, the Bureau of Chemical Safety (BCS) in Health Canada’s Food Directorate determined that a PoD of 10 µg/g MH THg represented an “*approximate threshold*”^17^ for neuropsychological dysfunction from prenatal MeHg exposure (Feeley & Lo 1998; Health Canada 1997, 2007). This PoD of 10 µg/g MH THg relied on four lines of evidence:

- evidence of effects at < 10 µg/g MH THg in outcome domains of language, attention, and memory from 7 year-old children of Faroe Islands Cohort 1, based on statistically significant adverse regression coefficients in a reduced sample including only observations with exposure < 10 µg/g MH THg (see “*Low-Level Exposure*” in Table 4 of Grandjean et al. 1997);
- a PoD range of 10–20 µg/g MH THg selected by the WHO IPCS 1990 report for evidence of delayed motor development in children from the Iraq poisoning incident and the New Zealand cohort (WHO 1990);
- a BMDL_10_ of 11 µg/g MH THg selected by the US EPA as a PoD for neurodevelopmental effects in children from the Iraqi poisoning incident for the derivation of their 1997 oral RfD (US EPA 1995, 1997a, b);
- an effect level > 12 µg/g MH THg based on a decrease in the percentage of 2 year-old children from the Seychelles Pilot study with normal scores on the Denver Developmental Screening Test (DDST) (Myers et al. 1995a, b); however the BCS noted that there were no apparent adverse effects observed in 1.6 year-old children (reported as 19 months) of the Seychelles Main Cohort (Myers et al. 1997)

The approximate threshold of 10 µg/g MH THg was converted to 40 µg/L MB THg based on a hair:blood ratio of 0.250:1^18^ and then to an oral intake of 1 µg/kg-bw/day MeHg employing the same one-compartment TK model first developed by the WHO (WHO 1990) and the US EPA (US EPA 1995), but with slightly different inputs (Health Canada 1997) (see Table 1). The BCS applied a total UF of 5 to account for inter-individual variability to arrive at an oral pTDI of 0.2 μg/kg-bw/day MeHg (Feeley & Lo 1998; Legrand et al. 2010).

The BCS pTDI and supporting PoDs have been reaffirmed several times since 1997 and have not changed from the initial derivation (Health Canada 1999, 2003, 2007). In 1999, the BCS considered the newly published 1999 oral MRL of 0.3 µg/kg-bw/day MeHg derived by the ATSDR (1999). The BCS reviewed the supporting Seychelles study used for the MRL derivation and concurred with ATSDR that there was no evidence of adverse effects in the reported range of 6.8–15.3 µg/g MH THg (Health Canada 1999). The BCS, therefore, determined that the previous “*estimated NOAEL*” of 10 µg/g MH THg and derived pTDI of 0.2 µg/kg-bw/day MeHg were appropriate and did not require revision (Health Canada 1999). In 2003, the BCS considered results from updated BMD modelling of the Faroe Islands Cohort 1 data reported in a review by the NAS (2000) and the US EPA (US EPA 2001b) (Health Canada 2003). The BCS considered the BMDL_05_ of 58 µg/L CB THg based on BNT score recommended by the NAS, which corresponded to 12 µg/g MH THg after conversion using a hair:cord blood ratio of 0.200:1.^19^ The BCS stated that the NAS-recommended PoD was still in the range of 10–12 µg/g MH THg hair and supported the earlier BCS PoD of 10 µg/g MH THg (Feeley & Lo 1998; Health Canada 1997). In a dietary risk assessment report from 2007 (Health Canada 2007) the BCS again reaffirmed their original pTDI of 0.20 µg/kg-bw/day MeHg based on close agreement with JECFA’s pTDI of 0.23 µg/kg-bw/day MeHg derived in 2004 (FAO/WHO 2004).

A later summary review made explicit that the BCS pTDI of 0.20 µg/kg-bw/day for MeHg could also be expressed as biomarker guidance values of 8 µg/L MB THg and 2 µg/g MH THg (Legrand et al. 2010), which can be considered as the equivalent to TRVs for blood and hair, respectively. These biomarker TRVs were derived by dividing the PoD in blood (40 µg/L) and hair (10 µg/g) by a UF of 5. The TRVs for neurodevelopmental effects were initially established for pregnant women and on a precautionary basis have been applied more broadly to women of child-bearing age, nursing mothers, and children,^20^ which were considered to represent the most sensitive subgroups for this endpoint (Legrand et al. 2010). PoDs and TRVs established by the BCS for neurotoxicity in the general population of adults are discussed in greater detail in Supplementary File S1.

### European Food Safety Authority (EFSA)

In 2004, EFSA used the pTWI of 1.6 µg/kg-bw/week derived by JECFA in 2004 (FAO/WHO, 2004) without conducting a *de novo* evaluation of neurodevelopmental epidemiology data (EFSA 2004). In 2012, EFSA published an updated opinion that involved a more thorough review of the available epidemiological data published since 2004 (EFSA 2012). For the Faroe Islands, the newer data included results for children at 14 years from Cohort 1 (Debes et al. 2006; Julvez et al. 2010; Murata et al. 2004), re-analyses of data for children at 7 years and 14 years of age from Cohort 1 to account for PCB exposure and fish intake (Budtz-Jørgensen et al. 2007; Grandjean et al. 2012), and data for children at 7 years from the smaller Cohort 2 (Budtz-Jørgensen et al. 2010). Collectively, EFSA considered that these new data supported the previous findings of adverse neurodevelopment in Faroe Islands Cohort 1 at 7 years but with a diminished effect size, while the findings from Faroe Islands Cohort 2 were more uncertain. Based on the re-analyses of the Faroe Islands Cohort 1 data that included adjustment and stratification for PCB exposure, EFSA concluded that the “*assessment of Faroese Cohort 1 and 2 together did not identify major confounding from PCB exposure*” (EFSA 2012). EFSA selected the PoD derived by JECFA in 2004 (FAO/WHO, 2004) and stated, “*it could not identify a better point of departure from the Faroese studies than the BMDL*_*05*_* of 12 mg/kg in maternal hair that has been selected previously by JECFA*” (p.128 of EFSA 2012).

Newer studies from the Seychelles included many analyses of the Main Cohort for children at different ages: at 5.5 years (Davidson et al. 2004), 9 years (Huang et al. 2005, 2007; Thurston et al. 2009; van Wijngaarden et al. 2006, 2009), 10.5 years (Davidson et al. 2008a, 2008b), and 17 years (Davidson et al. 2010, 2011). The re-analyses and assessment at later follow-up from the Seychelles Main Cohort replicated the previous findings that there were no clear adverse neurodevelopmental effects “*associated with prenatal mercury exposure*”; however, uncertainties regarding potential negative confounding by fish nutrients could not be assessed in the Seychelles Main Cohort since these variables were not collected. EFSA reviewed new results from the Seychelles Nutrition Cohort 1 at child age of 9 and 30 months (Davidson et al. 2008a, b; Lynch et al. 2011; Stokes-Riner et al. 2011; Strain et al. 2008), which included data on biomarkers for fish nutrients, such as omega-3 fatty acids and selenium, and therefore, could inform about potential confounding by these factors. EFSA considered that the Seychelles Nutrition Cohort 1 data supported a possible adverse effect at > 11 µg/g MH THg primarily based on spline curve analyses demonstrating that the regression coefficient of maternal omega-3 fatty acids on child psychomotor index (PDI) score at 30 months went from beneficial to adverse at prenatal mercury exposure > 11 µg/g MH THg (Lynch et al. 2011). This finding partly formed the basis for EFSA’s PoD selection described below. The assessment of the Nutrition Cohort 1 children at 5 years for other outcomes did not show evidence of adverse effects on neurodevelopment (Strain et al. 2012).

In addition to the Faroe Islands and Seychelles data, EFSA evaluated published data from other higher- and lower-exposed cohorts (Table 24 of EFSA 2012). Among populations with higher mercury biomarker levels in the same range as the Faroese and Seychelles studies, EFSA reviewed five publications from a cohort of Inuit children from Nunavik in northern Quebec, Canada (Boucher et al. 2010, 2012; Després et al. 2005; Plusquellec et al. 2010; Saint-Amour et al. 2006). The EFSA opinion also considered studies of populations with much lower mercury exposure including from Krakow, Poland (Jedrychowski et al. 2006, 2007), the Project Viva cohort from Boston (Oken et al. 2005, 2008), the New Bedford cohort from southern coastal Massachusetts (Sagiv et al. 2012), the Oswego cohort from northern New York State (Stewart et al. 2003, 2006), and the ALSPAC cohort in southwest England (Daniels et al. 2004). However, EFSA neither conducted a dose–response characterization nor selected a PoD based on these data, but rather stated that “*a few, but not all, studies found associations with mercury at levels lower than those reported in the Faroe Islands and Seychelles cohorts, but the overall picture at low-level exposure does not provide information to allow conclusions. In addition, there are indications of beneficial effects of fish consumption…these studies did not provide a better basis for dose response assessment than the studies in the Faroe Islands and Seychelles*” (EFSA 2012). Therefore, the EFSA 2012 opinion continued to rely on PoDs selected from studies in the higher-exposed populations from the Faroe Islands and Seychelles.

Based on two pivotal PoDs (i.e. the BMDL_05_ of 12 µg/g MH THg from Faroe Islands Cohort 1 (previously selected by JECFA) and the NOAEL of 11 µg/g MH THg from the Seychelles Nutrition Cohort 2), EFSA calculated an average PoD of 11.5 µg/g MH THg (EFSA 2012). This was converted to 46 µg/L MB THg based on a hair:blood ratio of 0.250:1^21^ and then to an oral PoD of 1.2 µg/kg-bw/day MeHg using the same TK model from the WHO (WHO 1990), but with custom EFSA inputs (see Table 1) (EFSA 2012). After applying a composite UF of 6.4 for uncertainty in TK (UF = 2 for uncertainty in the hair:blood ratio, UF = 3.2 for interindividual variation in TK), EFSA derived to a TDI of 0.19 µg/kg-bw/day MeHg (Table 1), which was expressed as a TWI of 1.3 µg/kg-bw/week MeHg (EFSA 2012). (*Note:* The EFSA opinion used the non-specific term “*mercury*” when referring to biomarker levels and the oral TWI. Consistent with other sections of this review, we inferred the specific chemical form of mercury, either THg or MeHg, based on the type of measurement and the conversion factors applied, and have accordingly specified the appropriate form throughout the section above.)

## Comparison of the PoDs and TRVs used for risk assessment

The oral TRV values for neurodevelopmental effects of prenatal MeHg exposure, as presented in Table 1, show close alignment across various risk assessment organizations. These oral TRVs (Table 1) range narrowly from a low of 0.10 µg/kg/d MeHg established by both the US EPA and ATSDR to a high of 0.23 µg/kg/d MeHg, as derived by JECFA. The same UFs can also be applied to the biomarker PoDs for maternal hair and blood to derive corresponding biomarker TRVs. The resulting ranges for each biomarker TRVs in maternal hair and maternal blood are 1.2–2.2 µg/g MH THg, and 4.6–9.2 µg/L MB THg respectively (see Table 4). Only the US EPA explicitly selected PoDs and derived TRVs for CB; therefore, no comparison between risk assessment organizations can be made for this biomarker.

**Table 4:** PoDs and theoretical TRVs expressed as concentrations in biomarkers (MH, CB, MB) and as oral intake sorted in order of smallest to largest oral TRV (see Table 1 for additional details) and the overall range for each parameter shown in the yellow highlighted row

Organization	MH (µg/g THg)			CB (µg/L THg)			MH:MB ratio (where MH÷ratio = MB)	MB (µg/L THg)			CF (where MB*CF = oral)	Oral (µg/kg-bw/day MeHg)		
	PoD_MH_	÷ UF	TRV_MH_	PoD_CB_	÷ UF	TRV_CB_		PoD_MB_	÷ UF	TRV_MB_		PoD_oral_	÷ UF	TRV_oral_ (sorted smallest to largest)
US EPA low	9 ^a^	10	0.9	46 ^b^	10	4.6	N/A	46 ^c^	10	4.6	0.0186	0.86	10	0.1 ^d^
ATSDR	5.6	3	1.9	N/A	N/A	N/A	0.203	27.6	3	9.2	0.0150	0.41	3	0.1 ^e^
US EPA high	12 ^a^	10	1.2	79 ^b^	10	7.9	N/A	79 ^c^	10	7.9	0.0186	1.47	10	0.1 ^d^
EFSA	11.5	6.4	1.8	N/A	N/A	N/A	0.250	46.0	6.4	7.2	0.0265	1.219	6.4	0.19
HC	10	5	2.0	N/A	N/A	N/A	0.250	40.0	5	8.0	0.0246	1.0 ^f^	5	0.20
JECFA	14	6.4	2.2	N/A	N/A	N/A	0.250	56.0	6.4	8.75	0.0265	1.484	6.4	0.23
Range (min–max)	**5.6–14**	**3–10**	**0.9–2.2**	**46–79**	**10**	**4.6–7.9**	**0.203–0.250**	**28–79**	**3–10**	**4.6–9.2**	**0.0150–0.0265**	**0.41–1.48**	**3–10**	**0.1–0.23**

^a^BMDL_05_ values from 9–12 µg/g MH THg were calculated from maternal hair in Faroe Islands Cohort 1 (see footnote ‘a’ in Table 1)

^b^BMDL_05_ values from 46–79 µg/L CB THg were calculated from cord blood in Faroe Islands Cohort 1 (see footnote ‘d’ in Table 1)

^c^US EPA assumed equivalent concentrations between CB and MB; therefore, the PoD_CB_ range was also used for PoD_MB_ (see footnote ‘e’ in Table 1)

^d^Reported as 0.1 µg/kg-bw/day from: “*the calculated RfD values converge at the same point: 0.1 µg/kg/day*” from p.4–60 and in Table 4–8 on p.4–61 of the US EPA (2001b). Based on the range of low to high PoD_oral_ of 0.86 to 1.47 µg/kg-bw/day MeHg and applying the UF of 10, the reported candidate low to high oral RfDs would be 0.09 to 0.15 µg/kg-bw/day MeHg rounded to 2 decimals

^e^The oral MRL of 0.10 µg/kg-bw/day was rounded down from 0.137 (i.e., 0.41 µg/kg-bw/day ÷ UF3 = 0.137 µg/kg-bw/day)

^f^Rounded up from 0.984: (40 µg/L) * (CF of 0.0246) = 0.984 µg/kg-bw/day

The JECFA evaluation included other endpoints, such as reproductive toxicity and immunotoxicity, however JECFA considered neurodevelopmental toxicity associated with prenatal exposures to be the most sensitive health effect for risk assessment (WHO, 2004). The EFSA evaluation also considered other health effects, including data from animal studies and epidemiology for cardiovascular and immunological outcomes, but concluded that human studies of prenatal exposure and neurodevelopmental effects were the most relevant for the derivation of a TRV (EFSA 2012). The former neurodevelopmental PoD and TRV established by the US EPA (1997b) were based entirely on data from the Iraq poisoning incident while the more recent PoDs and TRVs from the US EPA (2001b) and other organizations have moved away from the Iraqi data and instead relied on dose–response data from birth-cohorts in New Zealand, Faroe Islands (Cohort 1), and the Seychelles (Main Cohort). The most recent reviews by EFSA (2012) and the ATSDR (2024) reviewed many additional studies that were not previously considered by other organizations, including results of later cohorts from the same populations in the Faroe Islands (Cohort 2) and Seychelles (Nutrition Cohort 1) as well as results from cohorts in other locations. However, in the end, the PoDs and TRVs from both EFSA and ATSDR continued to rely primarily on data from New Zealand, Faroe Islands Cohort 1, and/or the Seychelles Main Cohort (ATSDR 2024; EFSA 2012).

The basis for selecting PoDs by the US EPA (2001b) and ATSDR (2024) could be considered the most computationally robust since they were based on BMD modeling of multiple outcomes using individual participant data from the same three cohorts: Faroe Islands Cohort 1, Seychelles Main Cohort, and New Zealand. The US EPA PoDs were based on a range of cord blood BMDL_05_ values from 46–79 µg/L CB THg (or alternatively a range of maternal hair BMDL_05_ values from 9–12 µg/g MH THg from the same outcome tests) estimated from BMD modelling of individual participant data from the Faroe Islands Cohort 1 at 7 years, with supporting BMDL_05_ values from New Zealand at 6 years, and Seychelles Main Cohort at 5.5 years (US EPA 2001b). In contrast, the ATSDR selected a maternal hair PoD of 5.6 µg/g MH THg which was derived from a pooled multi-domain regression coefficient of − 0.18 IQ points per increase in 1 µg/g MH THg from the meta-analysis by Axelrad et al. (2007) and application of a critical effect (i.e., BMR) of − 1 IQ point to calculate a PoD of 5.6 µg/g MH THg (i.e., PoD = BMR/β = − 1/− 0.18) (ATSDR 2024). After converting to PoDs in MB and applying UFs (UF10 for US EPA, UF3 for ATSDR), both the US EPA and ATSDR arrived at the same oral TRV of 0.1 µg/kg-bw/day MeHg despite the different approaches used (Tables 1 and 4).

## Sensitivity analyses: supplementary PoDs

The PoDs and TRVs presented in Tables 1 and 4, as established by various risk assessment organizations, show close agreement within a relatively narrow range. This consistency reinforces the credibility of the selected values and the continued use of these PoDs and TRVs in risk assessment. To further substantiate these findings, a validation exercise was conducted to derive several additional PoDs de novo based on previously published meta-analyses of observational studies (Axelrad et al. 2007; Coull et al. 2003; Kopylev & Segal 2025) and updated BMD modelling of adverse neurological scores in children prenatally exposure during the Iraq poisoning incident (Marsh et al. 1987). The resulting supplementary PoDs are compared with those currently used by risk assessment organizations in the sections below.

### BMDLs calculated from the Axelrad et al. (2007) and Kopylev & Segal (2025) meta-analyses of regression coefficients

Estimation of BMDLs from linear regression coefficients of continuous outcomes first requires that the BMR is expressed on the same scale as the coefficients. The BMDL_05_ values used by the US EPA in Table 2 were based on a BMR = 5% added risk^22^ with an abnormal test score background incidence of P(0) = 5% (NAS 2000; US EPA 2001b). Based on methodology proposed by Crump to equate BMD modelling of binary and continuous variables (Crump 1995, 2002; Crump et al. 2000), it can be shown that a BMR of 5% extra risk with a background abnormal incidence P(0) of 5% corresponds mathematically to a change in sample mean test score of − 0.363 SDy which corresponds to − 5.45 IQ points since IQ scores have an SD of 15 (see Supplemental File S3.A). This IQ decrease of − 5.45 IQ points which is equivalent to a BMR of 5% extra risk is larger than the critical effect size (i.e., BMR) of − 1 IQ points used by the ATSDR for their PoD calculation (Table 1) and would have resulted in a correspondingly larger PoD when using the ATSDR approach.^23^ However, the BMDL_05_ values used by the US EPA were based on the lower limit of the 90% CI of the estimated BMDs, while the ATSDR used the central tendency coefficient from the pooled meta-analysis. Previous publications have shown that under simple assumptions of a linear dose–response, reported linear regression coefficient (β) and their 90% CI lower limit (LCL) and upper limit (UCL) can be used to directly estimate the BMD, BMDL, and benchmark dose upper confident limit (BMDU) as follows: BMD = BMR/(β), BMDL = BMR/(β_90% LCL_), BMDU = BMR/(β_90% UCL_) respectively (Crump 1995; Crump et al. 1998; Grandjean et al. 2022).

Using the Axelrad et al. (2007) central tendency pooled meta-analysis coefficient of − 0.18 IQ points per 1 µg/g increase in MH THg and its 90% LCL of − 0.331 IQ points (derived using a z-distribution; see Table S4A.2 in Supplemental File S4A), the BMDL_05_ corresponding to a BMR of 5% added risk (− 5.45 IQ points) is 16.5 µg/g MH THg. When applying a more conservative t-distribution the 90% LCL shifts slightly lower to − 0.344 IQ points (see Table S4A.2 in Supplemental File S4A), yielding a BMDL_05_ of 15.8 µg/g MH THg. Validation of the Axelrad et al. (2007) meta-analysis using frequentist methods and the ‘rma.mv’ function in the {metafor} R package (Viechtbauer 2010) produced 90% LCLs of − 0.502 and − 0.481 IQ points based on t- and z-distributions, respectively (see Table S4A.2 in Supplemental File S4A), corresponding to slightly lower BMDL_05_ values of 10.9 and 11.3 µg/g MH THg, respectively.

However, in random-effects meta-analyses, the heterogeneity or dispersion of the true effect is more appropriately characterized by the prediction interval (PI), which accounts for both sampling error and other sources of variance (e.g. between-study heterogeneity), and the PI is therefore generally wider than the CI. Reporting a PI is generally recommended for characterizing the heterogeneity for a random-effects meta-analysis pooled estimate (Borenstein 2023; Botella & Sánchez-Meca 2024; IntHout et al. 2016). Although Axelrad et al. (2007) did not report a PI for their pooled estimate, a 90% PI can be estimated based on their published data (2007) and from the validation meta-analysis using the ‘rma.mv’ function in the {metafor} R package. Based on slightly different assumptions used, the resulting 90% PIs ranged from − 0.441 to 0.081 up to − 0.750 to 0.288 IQ per 1 µg/g increase in MH THg (see Table S4A.2 and forest plots in Supplemental File S4A) with beneficial direction upper limits of the 90% PIs suggesting substantial heterogeneity in the pooled regression coefficients from this data.

For comparison, a more recent meta-analysis by Kopylev & Segal (2025) pooled three regression coefficients each from two cohorts assessing performance on the BNT with and without cues. The resulting pooled regression coefficients were − 0.033 (95% CI − 0.074 to 0.007) and − 0.016 (95% CI − 0.056 to 0.023), respectively, expressed as changes in BNT scores per 1 µg/L increase in MB THg (reported as “*MeHg*” in Kopylev & Segal (2025) but measured as THg in all studies and so THg is used here). After rescaling to the same units of Axelrad et al. (see Supplemental File S5) and using 90% vs 95% CIs, these pooled coefficients correspond to − 0.396 (90% CI − 0.804 to 0.012) and − 0.192 (90% CI − 0.590 to 0.206) IQ points per1 µg/g increase in MH THg. Using the above BMR of − 5.45 IQ points and the 90% LCL, yields BMDL_05_ values ranging from of 6.8 to 9.2 µg/g MH THg. Additionally, Kopylev & Segal (2025) reported a smaller pooled coefficient in the language/verbal function subdomain of − 0.0085 IQ points (95% CI − 0.0167 to − 0.0003) per 1 µg/L increase in MB THg, based on 11 coefficients from 8 cohorts. When standardized to MH THg units and using 90% CIs, this corresponds to − 0.034 IQ points (90% CI − 0.062 to − 0.006) per 1 µg/g increase in MH THg, resulting in a substantially higher BMDL_05_ of 88.6 µg/g MH THg (Supplemental File S5). The Kopylev & Segal study did not report the PIs and information on the random-effects variance was not provided so PIs could not be estimated for these pooled coefficients.

Overall, the meta-analytical results from the Axelrad et al. (2007) dataset, yielded BMDL_05_ values ranging from 10.9 to 16.5 µg/g MH THg based on the 90% LCLs (− 0.502 to − 0.331 IQ points). Similarly, results from the more recent meta-analysis by Kopylev & Segal (2025) produced comparable BMDL_05_ values of 6.8 to 9.2 µg/g MH THg, based on the 90% LCLs of the rescaled pooled regression coefficients for BNT scores (90% LCL: − 0.804 to − 0.590), expressed in the same units as Axelrad et al. (2007). These BMDL ranges shower reasonable agreement with the range of maternal hair PoDs from 6 to 14 µg/g MH THg reported in Tables 1 and 4, despite differences in datasets and methodological approaches used for their derivation.

However, it should be noted that most 90% UCLs for the pooled regression coefficients from both of these meta-analyses were in the beneficial direction (β > 0), corresponding to an increase in IQ per 1 µg/g increase in MH THg (see Table S4A.2 in Supplemental File S4A, and Supplemental S5), which would result in *undefined* BMDUs^24^ (Coull et al. 2003; Crump et al. 2000). Furthermore, the Axelrad et al. meta-analysis predictive distribution for the PIs estimate that 13–21% of regression coefficients from future studies could be in the direction of benefit (β > 0), indicating substantial heterogeneity around the true effect, despite 14 of 15 regression coefficients showing adverse associations (β < 0; see Table S4A.2 in Supplemental File S4A.1). This heterogeneity, as illustrated by the beneficial direction upper limit 90% CIs and PIs of the regression coefficients, indicates that BMDLs derived from the adverse direction lower limit 90% CIs from this data represents a conservative approach. Accordingly, PoDs based on these methods are likely to be protective against potential neurodevelopmental effects of prenatal MeHg exposure.

### BMDLs calculated from the Coull et al. (2003) meta-analysis of inverse BMD/BMDLs

Table 2 and related text of this review described preliminary results by the NAS committee of an “*integrative analysis*” of BMDs from the Faroe Islands Cohort 1, Seychelles Main Cohort, and New Zealand cohort (NAS 2000). While the NAS committee considered the “*integrative analysis*” methodology to be too exploratory for the derivation of the US EPA oral RfD (NAS 2000), the details of this analysis were later published in a study by Coull et al. (2003). Similar to a later publication by Axelrad et al. (2007), Coull et al. (2003) also conducted a Bayesian hierarchical multi-level meta-analysis. However, rather than pooling standardized linear regression coefficients as in Axelrad et al. (2007), Coull et al. (2003) chose to synthesize the corresponding BMDs but they specifically pooled the inverse of the BMDs (1/BMDs) which are more normally distributed, can better handle very large values of BMD, and are a simple transformation of these linear regression coefficients (i.e., 1/BMD = β/BMR, re-arranged from BMD = BMR/β) (Coull et al. 2003; NAS 2000).^25^ From Table 2 of Coull et al. (2003), the meta-analytical pooled BMD and BMDL were reported as 21.0 and 7.7 µg/g MH THg respectively. These values were derived from the modelled posterior distribution of the inverse BMD (1/BMD), with the mean 1/BMD value of 0.048 and the upper 5th percentile 1/BMDL value of 0.130, reflecting the mean and lower 5th percentile of the BMD posterior distribution. The BMDL of 7.7 µg/g MH THg was previously reported in the NAS report as a BMDL of 8 µg/g MH THg for the “*integrative analysis*” (NAS 2000).

Although Coull et al. (2003) did not explicitly report a pooled BMDU, it can be approximated from the lower 5th percentile of 1/BMD distribution, assuming a normal distribution and applying a z-value of 1.645 for a 90% CI.^26^ Based on this approach, the lower 5th percentile of the 1/BMDs distribution is − 0.0346, yielding a BMDU of 1/− 0.0346 or − 29.1 µg/g MH THg, which is characterized as an *undefined* BMDU. This mirrors the situation in the meta-analysis by Axelrad et al. (2007), where the 90% UCLs for the pooled regression coefficients were in the direction of benefit and would, therefore, result in similarly *undefined* BMDUs. Further, the PIs for the modelled 1/BMDs would be even wider when accounting for both sampling error (SE) and the additional heterogeneity introduced by random effects in the multi-level model used by Coull et al. (2003). Specifically, the model included two random effects variance components with standard deviations of 0.0810 and 0.0244 (in units of 1/BMD). As with Axelrad et al. (2007), accounting for these sources of variability would result in a wider 90% PI, increasing the upper 5th of 1/BMDs and yielding a lower, more conservative BMDL. At the same time, the 90% PI would also result in a more negative value for the lower 5th 1/BMD and a higher probability of a negative/*undefined* BMDU reflecting a potential beneficial direction effect.

Similar to the meta-analysis of regression coefficients from Axelrad et al. (2007), the Coull et al. meta-analysis of 1/BMDs resulted in a pooled estimated BMDL of 7.7 µg/g MH THg, which aligns with the maternal hair PoDs currently used in risk assessment, ranging from 5.6 to 12 µg/g MH THg (see Table 4). However, similar to the results from Axelrad et al. (2007), the wide 90% CIs and PIs for the 1/BMD include negative values, resulting in *undefined* BMDU and indicating a potential direction of benefit. This pattern suggests underlying heterogeneity in the true BMD, with some probability of a beneficial effect (i.e., increased IQ with higher prenatal MeHg exposure). Consequently, deriving BMDLs from the lower bounds of these distributions represents a conservative approach. PoDs based on BMDLs from the Coull et al. meta-analysis are therefore likely to be protective against potential neurodevelopmental effects of prenatal MeHg exposure.

### Bayesian model-average BMDLs for abnormal child neurological scores from the Iraq poisoning

Neurodevelopmental data from the Iraq poisoning incident (Marsh et al. 1987) have also been reconsidered in this review for comparison with the PoDs presented in Tables 1 and 4. As discussed in the section for the US EPA 1995 RfD, the US EPA (1997b) originally based its oral RfD on BMD modelling of binary outcome data for 81 mother–child pairs exposed to dietary MeHg during gestation in the 1971–1972 Iraq MeHg poisoning incident. The BMD model used a single Weibull model with frequentist maximum likelihood estimation and a BMR of 10% extra risk for both individual and combined outcomes (see Table 6–5 in US EPA (1997b). However, due to subsequent critiques of these data, particularly regarding the reliability of the maternally reported outcomes, the US EPA shifted its reliance to BMD modeling of birth-cohort data from New Zealand, the Faroe Islands, and Seychelles (see Tables 1 and 4). Despite most regulators moving away from the Iraq poisoning data for their neurodevelopmental PoD, the Iraqi data remains valuable as a sensitivity analyses for PoD selection, especially given the relatively high confidence in the reported neurological scores since these data were based on physical examination by clinicians who were blinded to THg exposure status of the mother–child pairs (Marsh et al. 1981, 1987).

For our analysis here, updated BMD modelling was conducted using the incidence of dichotomized neurological scores, defined as either normal (scores 0–3) or abnormal (scores ≥ 4), from Marsh et al. (1987) along with the five exposure categories selected by the US EPA (1997b) (see Supplemental Files S6.A and S6.B for details). As a sensitivity analysis, BMD estimates were also generated for alternative definition of an abnormal neurological score (≥ 5) consistent with previous BMD modelling by the US EPA (1997b). The updated BMD modeling applied a Bayesian model-average approach via the US EPA BMDS platform (US EPA, n.d.-a), using a BMR of 5% extra risk to align with previous MeHg BMD estimates (Tables 2).^27^ The resulting BMDL_05_'s were 12.9 and 17.5 µg/g MH THg, depending on whether the abnormal score was set at ≥ 4 or ≥ 5, respectively (Supplemental File S6A). The lower bound of this range (12.9 µg/g MH THg) aligns reasonably well with the MH THg PoDs reported in Tables 1 and 4. Despite valid uncertainties with these data from the Iraq poisoning incident, the updated Bayesian BMD modelling using the least biased outcome from this dataset (increased abnormal child neurological scores) provides additional support for the range of PoDs and TRVs currently used in risk assessment.

## Discussion

This review summarized the history and basis of PoDs and TRVs for neurodevelopmental effects of prenatal MeHg derived by different risk assessment organizations. There is relatively high consistency between the PoDs and TRVs in Tables 1 and 4 despite different pivotal data used for PoD selection, different biomarker conversions, and different uncertainty factors applied for TRV derivation. Furthermore, sensitivity analyses using pooled regression coefficients from meta-analyses by Axelrad et al. (2007) and Kopylev & Segal (2025), the inverse of the estimated BMDs from a meta-analysis by Coull et al. (2003), and binary outcome data for neurological scores from the Iraq poisoning incident (Marsh et al. 1987, and Supplemental File S6) also resulted in supplemental PoDs within the same range as those currently used in risk assessment (Tables 1 and 4). While there was strong agreement between the PoDs and TRVs selected by different organizations, several uncertainties remain specifically related to the use of observational epidemiology data in PoD derivation. These uncertainties are discussed below.

1. All of the PoDs in Tables 1 and 4 were based primarily on data from three birth-cohorts published in the 1980s and 1990s: New Zealand cohort, Faroe Islands Cohort 1, and Seychelles Main Cohort. Since then, there have been hundreds of new analyses published, including additional data at later follow-up times from these same birth-cohorts, data from newer birth-cohorts in the Faroe Islands and Seychelles populations, as well as data from several birth-cohorts from different geographical locations with mercury exposures either within the range of exposures in the Faroe Islands and Seychelles populations or at lower exposures closer to general populations in Canada and the United States. Many of these newer birth-cohorts collected more extensive dietary information to allow for better statistical adjustment of nutritional factors in fish, such as selenium and polyunsaturated fatty acids (PUFA), which could have differential moderating effects on the association between neurodevelopment and MeHg exposure, depending on the level of fish intake. This is particularly important when considering that large variation in fish intake have been reported between the different cohorts, which could affect both the extent of MeHg exposure and the levels of these nutritional factors in these populations. While more recent evaluations by EFSA (2012), HBM4EU (Halldorsson et al. 2022), ATSDR (2024), and a recent umbrella review of systematic reviews by O’Connor et al. (2025) considered newer epidemiological datasets, a formal quantitative synthesis of these data was generally either not pursued due to one or more challenges, such as heterogeneity in the outcomes assessed and differences in the statistical models used and units for reporting results. One recent exception is a meta-analysis by Kopylev & Segal (2025) which included data from more recently published birth cohorts and pooled regression coefficients for BNT scores and tests of language/verbal function. However, this analysis was based on a relatively small number of estimates from a narrow subdomain with limited details on the meta-analysis methods, and the underlying systematic review was not available (Kopylev & Segal 2025). Therefore, future systematic reviews in this area are still needed and should continue to incorporate quantitative synthesis of all eligible epidemiological studies, with particular emphasis on newer data that adjusted for the potential moderating effect of fish consumption. In addition, all future work should ensure full transparency in the reporting of systematic review protocols and meta-analytical methods to support reproducibility and scientific rigor.

2. Several of the PoDs in Tables 1 and 4 were based on a subset of available test scores from the New Zealand cohort, Faroe Islands Cohort 1, and Seychelles Main Cohort which were primarily in the direction of adverse effect (as shown in Table 3). These data were subsequently used either for BMD modelling (NAS 2000; US EPA 2001b) or for a meta-analysis of regression coefficients (ATSDR 2024; Axelrad et al. 2007) (see Tables S2.1 to S2.4 in Supplemental File S2). However, each of these datasets reported additional results on multiple other neurodevelopmental tests but which were not considered in PoD selection^28^ including associations that were either null, in the direction of benefit, or uncertain direction. For example, substantial heterogeneity was observed in the 13 regression coefficients^29^ included in the meta-analysis by Axelrad et al. (2007), where the resulting PI estimated a 13–21% probability of a beneficial regression coefficient being observed in a similar future study (see Table S4A.2 in Supplemental File S4A). Therefore, a quantitative synthesis including all eligible estimates of association from these three cohorts could further increase this observed heterogeneity. While selecting PoDs based on the most adverse statistically significant effects can be a conservative precautionary approach appropriate for the purposes of risk assessment, characterization of the full distribution of reported associations (adverse, null, and beneficial regardless of p-value) would also better inform the uncertainties and heterogeneity of the data from which the PoDs were selected.

3. Nearly all of the PoDs discussed in this review and shown in Tables 1 and 4 (except ATSDR) relied on BMD modelling of the dose–response relationship using individual participant data. These BMD analyses were conducted either by the study authors themselves (Faroe Islands Cohort 1) or by researchers who were granted access to individual participant data (New Zealand and Seychelles Main Cohort). External validation of these BMD-based PoDs and any potential future BMD modelling of data from newer cohorts is not possible without access to individual participant data. Additionally, individual participant data would be needed to conduct BMD modelling using more current model-averaging approaches (WHO 2020) versus the single-model methods previously used for derivation of the BMD-based PoDs. The PoD derived by the ATSDR used a meta-analysis of published regression coefficients and therefore, could be at least theoretically validated as was done in this review (see Supplemental File S4A and S4B). However, PoDs from both approaches (BMD-based PoDs using individual participant data, PoDs from summary regression coefficients or meta-analyses of these coefficients) rely on the original modelling assumptions, which typically assumed a linear dose–response relationship. Neither of these current approaches allow the shape of the dose–response in the underlying data to be examined, including for potential non-linear relationships or identifying thresholds of effect. Researchers publishing on this topic should explore supplementing the traditionally reported single regression coefficients from linear models and additionally provide sufficient information to allow readers and risk assessors to independently conduct their own dose–response modelling to aid in PoD selection. For example, authors could report anonymized individual participant data for the covariate-adjusted outcome^30^ and the exposure biomarker. Such individual participant data provides essentially the same information as in covariate-adjusted scatter plots, which are sometimes reported by authors (e.g., Fig. 1 in Davidson et al. (1998) for the Seychelles Main cohort, Fig. 1 in Budtz-Jørgensen et al. (2003) for Faroe Islands Cohort 1) and would therefore not contravene data protection/sharing regulations for epidemiology studies any more than the published scatter plots. Alternatively, authors could report the summary estimates in a format more conducive to dose–response modelling, such as regression coefficients or covariate-adjusted mean test scores stratified by three or more categories of the exposure biomarker, with category mean/median used as the exposure metric for dose–response. It is not uncommon for authors to report this type of categorical exposure modelling, and has been recommended as a potential choice for dose–response modelling of summary epidemiology data in recent guidance by the WHO (2020)^31^ and EFSA (2024). Study authors are encouraged to use either of these alternative methods of data reporting, as this will aid in dose–response modelling and PoD selection by regulators and other users of these types of studies.

4. Variability in the BMR value used for neurodevelopmental outcomes in observational epidemiology studies has been noted in the literature. Previous risk assessments for MeHg have selected PoDs based on BMD estimates using the “*hybrid approach*” with binary outcome BMRs of 5% added risk for a background abnormal probability P(0) of 5% (NAS 2000; US EPA 2001b). In the “*hybrid approach*” this binary BMR equates to a continuous outcome BMR of 0.36 SDy and corresponds to a − 5.45 IQ point change in IQ tests (Supplemental File S3.A). However, a much wider range of continuous BMRs have been previously used for IQ tests in risk assessment of other chemicals, ranging from as small as − 1 IQ point for lead (Budtz-Jørgensen et al. 2013; EFSA 2010), from − 1 to − 3 IQ points for perchlorate (US EPA 2019), from − 1 to − 5 IQ points for manganese (Kullar et al. 2019), from − 2.8 to − 9.1 IQ points^32^ for PCBs (Jacobson et al. 2002), and from − 1 (Grandjean et al. 2022) up to − 13 IQ points for fluoride (Hirzy et al. 2017). A precautionary approach in risk assessment generally supports conservative assumptions, including the selection of a smaller BMR for neurodevelopmental effects (e.g., − 1 IQ point). For MeHg, the use of a lower, more conservative BMR, such as − 1 IQ point instead of the previously used BMR of − 5.45 IQ points, would have resulted in much lower PoDs than those in Tables 1 and 4. However, there is currently no clear consensus on the most appropriate BMR for this endpoint and other factors should also be considered, such as balancing the requirement to be health protective with the statistical power of typical observational studies to detect a given BMR. Setting a BMR below the sensitivity of the available observational studies would be questionable and contribute to increased uncertainty. The BMR is also affected by the definition of abnormal, which can vary across different datasets (e.g., abnormal IQ scores defined as ≤ 70 or − 2SD below the population mean, versus IQ scores ≤ 85 or − 1SD below the mean, or some other definition for abnormal). The risk assessment community would benefit from a more robust discussion and guidance on BMR selection, specifically for neurodevelopmental outcomes from observational epidemiological studies.

5. All PoDs and TRVs in Tables 1 and 4 relied on point-estimates for conversions between exposure metrics, including the hair:blood ratios and other parameters (e.g., body weight, elimination rate constant) included in the one-compartment TK model to convert blood THg to oral intake of MeHg. There is underlying variability in each of these factors that point-estimates cannot fully capture. Other approaches, such as probabilistic methods, may better characterize this variability or alternatively, more sophisticated physiologically based pharmacokinetic (PBPK) models such as in Pope & Rand (2021) could be explored.

6. The PoDs and TRVs used in risk assessment (Tables 1 and 4) are exclusively based on data from observational epidemiology studies. However, there is a substantial body of animal toxicity data investigating the neurodevelopmental effects of MeHg exposure during gestational and early postnatal periods (ATSDR 2024). The SAB review of the US EPA’s draft 1995 oral RfD explicitly recommended integrating findings from animal toxicity studies as supportive evidence along with observational epidemiology data when selecting PoDs and deriving a TRV (US EPA, 1997a). Subsequent attempts have been made to estimate alternative candidate TRVs from animal data including candidate oral RfDs of 0.01 to 0.05 µg/kg-bw/day derived by the US EPA from data on non-human primates (US EPA 2001a, 2001b) and oral MRLs of 0.009 and 0.04 µg/kg-bw/day derived by the ATSDR from mouse and rat studies respectively (ATSDR 2024). While these candidate animal-based TRVs (range: 0.009 to 0.05 µg/kg-bw/day) are far below the range of human data-based oral TRVs currently used in risk assessment (0.1 to 0.23 µg/kg-bw/day from Table 4), it should be noted that conservative default UFs from 100 to 1000 were applied to derive the animal-based TRVs. A critical discussion on the relevance of different animal models of MeHg toxicity and corresponding chemical-specific adjustment factors for key parameters, such as species-specific differences in MeHg brain:blood ratios (Newland et al. 2008), toxicokinetics in blood (Yamamoto & Shima 2009), and maternal–fetal transfer (Cambier et al. 2018) may result in refinement of the UFs and corresponding animal-toxicity based PoDs and TRVs to be closer to the epidemiology-based PoDs and TRVs in Tables 1 and 4. Future work refining the animal-toxicity based PoDs and TRVs could provide supporting information even if PoDs and TRVs for neurodevelopmental effects of MeHg continue to be based on birth-cohort studies.

## Summary

The comparison of PoDs and TRVs for neurodevelopmental effects of prenatal MeHg exposure demonstrates broad consensus among risk assessment organizations in their selection of PoDs and TRVs (Tables 1 and 4), despite differences in the underlying data used to derive those PoDs, and in the selection of UFs and conversion parameters. Sensitivity analyses conducted in this review identified supplementary PoDs within a similar range, lending additional support for the robustness of the PoDs and TRVs from Tables 1 and 4 currently used in risk assessment. The consistency in TRVs strengthens confidence that MeHg risk assessments are likely to yield comparable risk estimates, regardless of the specific TRV applied.

However, despite general alignment on PoDs and TRVs, significant methodological challenges remain, particularly in the application of observational epidemiological data used to derive these values. Many PoDs and TRVs are based on reviews of older datasets that relied on a limited number of data points. In addition, deriving PoDs from linear regression slopes rely on the assumption of a linear relationship, and deriving BMD-based PoDs from such linear regression slopes requires selecting a BMR—a decision that lacks consensus and can significantly affect the resulting BMD. These challenges are not unique to MeHg and carry broader implications for risk assessments of other chemicals that rely on observational epidemiological data.

To advance the field, it is essential that future work addresses these methodological limitations. In particular, there is a need for a comprehensive quantitative syntheses of neurodevelopmental outcomes associated with prenatal MeHg exposure based on a systematic review of all eligible data. In addition, authors of new observational epidemiological studies are strongly encouraged to provide sufficient detail when publishing results, including reporting estimates in a format that is better suited to facilitate independent dose–response modelling to help support transparent and robust PoD selection by readers and risk assessors.

## Supplementary Information

Below is the link to the electronic supplementary material.

Supplementary file1 (DOCX 110 kb)

Supplementary file2 (DOCX 118 kb)

Supplementary file3 (DOCX 136 kb)

Supplementary file4 (XLSX 60 kb)

Supplementary file5 (DOCX 510 kb)

Supplementary file6 (XLSX 18 kb)

Supplementary file7 (XLSX 18 kb)

Supplementary file8 (DOCX 245 kb)

Supplementary file9 (XLSX 170 kb)

## Data Availability

The data used for this review are either available in the cited references or in the provided Supplemental Files.

## Abbreviations

ADI: Allowable daily intake
ATSDR: US Agency for Toxic Substances and Disease Registry
BMD: Benchmark dose
BMDL: Lower limit for a given confidence interval on the BMD
BMR: Benchmark response
BNT: Boston naming test
CB: Cord blood
CF: Conversion factor
EFSA: European Food Safety Authority
FAO: Food & Agriculture Organization
HBGV: Health-based guidance value
HC: Health Canada
IPCS: International Programme on Chemical Safety
JECFA: Joint FAO/WHO Expert Committee on Food Additives
LCL: Lower limit for a given confidence interval
LOAEL: Lowest-observed-adverse-effect level
MB: Maternal blood
MeHg: Methylmercury
MH: Maternal hair
MRL: Minimal risk level
MSRC: Methylmercury Study Report to Congress
NOAEL: No-observed-adverse-effect level
PoD: Point of departure
pTDI: Provisional tolerable daily intake, used interchangeably with TDI
pTWI: Provisional tolerable weekly intake, used interchangeably with TWI
RfD: Reference dose
SAB: Science Advisory Board
THg: Total mercury
TK: Toxicokinetic
TRV: Toxicological reference value
TDI: Tolerable daily intake, used interchangeably with pTDI
TWI: Tolerable weekly intake, used interchangeably with pTWI
UCL: Upper limit for a given confidence interval
UF: Uncertainty factor
US EPA: US Environmental Protection Agency
US FDA: US Food & Drug Administration
WHO: World Health Organization

## Footnotes

1 ‘Toxicological’ is added in this review while the US EPA IRIS Glossary uses simply ‘Reference Value’ or RfV.

2 “*Abnormal*” for each outcome is defined as: time to walking (> 18 months), delayed time to talking (> 24 months), “*mental*” symptoms (Y vs N), seizures (Y vs N), abnormal neurological score (scores 4–11 vs normal score 0–3), the overall combined outcomes (“*abnormal*” score on 1 or more outcome vs normal on all outcomes).

3 The same TK model was used by the US EPA for blood to oral conversion in the derivation of the 2001 oral RfD, but with different point-estimate inputs.

4 The sign of the continuous outcome BMR will be determined by the direction of effect for the specific outcome variable: a BMR = -0.036 SDy when a lower score (or β < 0) is adverse and a BMR = 0.36 SDy when a higher score (or β < 0) is adverse. For most neurodevelopmental test scores, such as IQ scores, a lower score (or β < 0) indicates worse function so the appropriate BMR in this case would be -0.36 SDy.

5 More commonly reported as a ratio of 250:1 when the units are expressed as MH(µg/kg):CB(µg/L).

6 US EPA assumed equivalency between CB and MB THg without any conversion factor.

7 Re-scaled coefficient defined as ∆ IQ points by Axelrad et al. 2007 = (standardized coefficient as ∆SD_Y_) * (SD of 15 for an IQ score).

8 The pooled cognitive domain regression coefficient from Axelrad et al. (2007) was interpreted as a loss in IQ by these groups.

9 "*…uncertainty factors of 1.5 for pharmacokinetic and 1.5 for pharmacodynamic variability in the relationship between mercury hair levels and mercury ingestion rates, and a modifying factor of 1.5 for domain-specific differences between the Faroe Islands and Seychelles studies ((1.5* + *1.5)* × *1.5* = *4.5)*" (p.100 of ATSDR 1999).

10 The basis for categorized result was not explicitly stated, but assumed to be based on p < 0.05 for the regression coefficient.

11 From Eq[8] on p. A-68 of ATSDR 2024: “*PoD*_*hair*_ = *(-1 IQ point)/(-0.18 IQ points per increase of 1 µg Hg/g hair)* = *5.56 µg Hg/g hair*”.

12 Reported by ATSDR as a ratio of 203:1 for units expressed as MH(µg/kg):MB(µg/L).

13 The TK model reported as Eq[10] on p.A-68 in the ATSDR 2024 report is identical to the TK model by WHO (1990) used by all other groups (summarized in Table 2 in the current review). The model inputs used by ATSDR were provided in Table A-26 and on p.A-66 to A-67 of the ATSDR report.

14 Rounded down from 0.137 µg/kg-bw/day (i.e., 0.41 µg/kg-bw/day ÷ UF3 = 0.137 µg/kg-bw/day).

15 Table 2 in the JECFA review appears to summarize BMD estimates extracted from the US EPA 1997a, b oral RfD supporting document (US EPA, 1997b).

16 More commonly reported as a ratio of 250:1 when the units are expressed as MH(µg/kg):CB(µg/L).

17 Also reported as an “*approximate bench mark dose*” (Health Canada 1997) and a NOAEL (no observed adverse effects level) in Feeley & Lo (1998).

18 More commonly reported as a ratio of 250:1 when the units are expressed as MH(µg/kg):MB(µg/L).

19 More commonly reported as a ratio of 200:1 when the units are expressed as MH(µg/kg):CB(µg/L).

20 “*Children*” have been reported as ≤ 12 years (Health Canada 2007) and ≤ 18 years (Legrand et al. 2010) in different Health Canada MeHg risk assessment publications.

21 More commonly reported as a ratio of 250:1 when the units are expressed as MH(µg/kg):MB(µg/L).

22 The binary outcome BMR reported as 5% “*excess risk*” by the NAS and US EPA (NAS 2000; US EPA 2001b) was in fact 5% added risk, using the more current terminology for binary outcome BMRs.

23 Applying BMR 5% added risk (i.e., -5.45 IQ or -0.363SDy) to the Axelrad et al. pooled coefficient in this review was done to allow for direct comparison to the previously derived BMDL_05_ values, and should not imply this to be the most appropriate BMR for IQ loss or other adverse neurodevelopmental effects for risk assessment purposes. There remains considerable debate on this topic. Table S3A.3 in Supplemental File S3A shows a variety of different candidate BMRs.

24 For a negative BMR, as in the case of MeHg (BMR = -0.36 SDy, equivalent to -5.45 IQ points), the calculated BMD (BMR/β) or BMDU (BMR/β_90% UCL_) will approach an infinite positive value for the BMD or BMDU as β or the β_90% UCL_ approaches 0, and then becomes negative when β or β_90% UCL_ > 0 (beneficial direction). In such cases, the BMD or BMDU have been described as “∞” by Coull et al. (2003) or as *undefined* by Crump et al. (2000) and the NAS report (2000).

25 The BMD and BMDLs used for the Coull et al. meta-analysis reported in Table 1 of Coull et al. (2003) also show the central tendency BMD estimates for the Seychelles which were not reported in either the source study by Crump et al. (2000) or the NAS report (2000), and therefore not shown in Table 3 of the current review.

26 Assuming normality of the posterior distribution of 1/BMDs, the standard error for the 90% CI of 1/BMD can be calculated from: 1/BMDL = (upper 5th of 1/BMD distribution) = (mean of 1/BMD distribution) + SE*1.645, which can be rearranged to solve for SE as: SE = [(upper 5th of 1/BMD distribution)—(mean of 1/BMD distribution)] ÷ 1.645 = [(1/7.7)—(1/21)] ÷ 1.645 = 0.05. Using the calculated SE of 0.05 for the 90% CI of the posterior distribution of 1/BMD, the BMDU can be calculated similarly: BMDU = (lower 5th of 1/BMD distribution) = (mean of 1/BMD distribution)—SE*1.645 = (1/21)—(0.05)*1.645 = -0.0346 as the lower 5th percentile of 1/BMDs and BMDU = 1/-0.0346 = -29.1 µg/g MH THg.

27 The BMRs in Table 2 were for a binary BMR of 5% added risk and not % extra risk as modelled for the Iraq abnormal neurological scores.

28 New Zealand cohort assessed 23 outcome tests, but only reported coefficients for 5 of 23 tests and only p-values for the remaining 18 tests without information on the direction of effect (Table S2.1 in Supplemental File S2).

29 The Axelrad et al. meta-analysis was based on 15 coefficients, but 3 of these were collapsed to a composite measure so the actual meta-analysis was conducted on 13 coefficients (12 + 1 composite coefficient).

30 Outcome scores adjusted for all covariates except exposure.

31 See Sect. 5.2.3 Modelling observational data from epidemiological studies on pages 5–36 to 5–38 (WHO 2020).

32 Reported as binary outcome BMRs of 5% or 10% added risk for background abnormal probability P(0) of 5% or 16%, converted to IQ changes using the “*hybrid approach*” methods in Supplemental File S3A and S3B.
