# Supplementary material for: Neurodevelopmental effects of methylmercury (MeHg): a review of epidemiological points of departure (PoDs), toxicological reference values (TRVs), and key uncertainties in human health risk assessment
Source: Arch Toxicol. 2026 Mar 10;100(6):2191–219. doi: 10.1007/s00204-026-04345-8 (PMC13221417; doi:10.1007/s00204-026-04345-8)
Supplement: Supplementary file 1 — Supplementary file1 (DOCX 110 kb) [file 204_2026_4345_MOESM1_ESM.docx]

**Supplemental File S1.**

**PoDs and TRVs for Neurological Effects of MeHg Exposure in the General Population (i.e. adults)**

*Archives of Toxicology*

Neurodevelopmental effects of methylmercury (MeHg): A review of epidemiological points of departure (PoDs), toxicological reference values (TRVs), and key uncertainties in human health risk assessment

Blechinger, Scott R.^1^* (ORCID 0000-0002-4991-4597, Scopus ID 6506155596)

Singh, Kavita^2^ (ORCID N/A, Scopus ID 58382466900)

Afghan, Abdul^1^ (ORCID N/A, Scopus ID 58522410100)

Smith, Catherine A.^1^ (ORCID N/A, Scopus ID 46461849300)

^1^ Bureau of Chemical Safety, Food and Nutrition Directorate, Health Canada, Ottawa, Canada

^2^ Environmental Health Science and Research Bureau, Health Canada, Ottawa, Canada

*Corresponding author: scott.blechinger@hc-sc.gc.ca

The earliest point of departures (PoDs) and toxicological reference values (TRVs) for the health effects of methyl mercury (MeHg) exposure were primarily based on poisoning cases in adults from Japan (Minamata Bay and Niigata) and Iraq. Most risk assessment organizations subsequently arrived at more conservative lower PoDs and TRVs to protect against neurodevelopmental effects in children exposed prenatally to MeHg. While these lower PoDs and TRVs for this sensitive subgroup have largely replaced the earlier values based on adult neurotoxicity, the latter values may still be considered applicable for risk assessment of neurological effects in the adult general population. A brief summary of some of the PoDs and TRVs for the adult general population are provided below. Note that older publications reporting biomarker and oral mercury intakes often did not specify if the values referred to total mercury (THg) or MeHg. In this review, it was assumed that biomarker concentrations in blood and hair represented THg and oral intakes were MeHg unless otherwise specified in the source publication.

# Swedish Expert Group (1971)

In 1968, the Swedish National Institute of Public Health appointed an expert group (Swedish Expert Group or SEG here) to evaluate the toxicological and epidemiological health effects data related to exposure to MeHg from fish (SEG, 1971). A large part of the SEG report was based on information gathered in a study trip to Japan by several of the expert group members who reviewed Swedish translations of many of the previously unpublished Japanese-language adult case reports of MeHg poisoning from Minamata Bay and Niigata, and the SEG report also included interviews with many of the key Japanese clinical investigators. The work from this study trip was summarized in a Swedish language report (Berlin et al., 1969) and subsequently in the English-language SEG review (1971). A similar report containing much of the same information was also published by academics from the Swedish Karolinska Institute (3 of the authors were also on the SEG) under contract to the US EPA (US EPA, 1971).

Estimates of MeHg exposure based on fish intake from both Minamata Bay and Niigata were not used to assess risk due to unreliable data on fish consumption and MeHg levels in fish (p.187 of the report). Instead blood and hair THg, principally from Niigata cases, were used to assess the dose-response and establish thresholds for adult neurological effects (summarized in Chapter 9.2 of the report; p.165-180). Briefly, whole blood THg concentrations of 17 cases from Niigata were presented in Table 9.5 of the SEG report, including the date on neurological symptom onset and the date of blood sampling. Eight of the 17 cases had multiple blood THg measures over time. This data was graphically visualized in Figure 9.1 to extrapolate the blood levels at day 0 of symptom onset based on the assumption that exposure ceased at symptom onset. The highest day 0 extrapolated blood concentrations were > 2000 µg/L THg while the lowest three exposed cases (cases #17, 18, 19 in Figure 9.1 of the SEG report) had estimated blood levels ranging from 200 – 400 µg/L THg. Measures of THg in hair were also reported for 36 of the Niigata cases in Table 9.7 (p.175-177) with date of symptom onset and date of hair sampling to allow for extrapolation to day 0 in Figure 9.3 (p.178). From Figure 9.3, the day 0 hair mercury concentrations were estimated to fall between >200 to 1000 µg/g THg “as a general rule”. Only a single case (#30) had a hair mercury concentration < 200 µg/g, which was reported to be 52 µg/g THg at 1-30 days after the onset of disease symptoms.

The SEG also reviewed the available data on potential prenatal effects, including 22 reported cases of prenatal poisoning from Minamata Bay (Table 10.1 on p.203-204 of the SEG report). Data were reported on hair THg in the 22 putative cases for children 1-6 yrs old (5 – 100 µg/g THg) and their mothers (2 – 190 µg/g THg). Since the maternal and child hair samples were taken several years after birth, there was no reliable estimate of prenatal exposure from these data. A single case of prenatal poisoning from Niigata was also reported, with measured maternal hair levels of 290 and 82 µg/g THg at 2.5 months and 4 months, respectively, post-partum and estimated maternal hair levels at birth of approximately 600 µg/g THg (p.204). The SEG considered “the data on prenatal poisoning [to be] particularly limited” (p.29). Ultimately, no PoD or TRV were derived for neurodevelopmental effects following prenatal MeHg exposure, while the safety factor of 10 was applied to the PoD for adult neurotoxicity, in part, to account for this uncertainty (p.276 of the SEG report).

The SEG noted that the underlying Japanese source materials reporting the blood and hair THg concentrations for the Niigata adult poisoning cases provided somewhat inconsistent information on mercury levels and dates (e.g. duration of exposure, onset of symptoms, etc.), and therefore, these data were considered uncertain by the SEG. Nonetheless, based on the Niigata data in adults, the SEG determined that neurological symptoms may occur in sensitive individuals as low as 200 µg/L THg in blood and approximately 50 µg/g THg in hair.^[[1]](#footnote-2)^ The estimated lowest neurological effect level of 200 µg/L THg in blood was then converted to a level in red blood cells (RBCs) of 400 µg/kg (reported as 0.4 µg/g THg) by assuming the concentration of THg in whole blood is 50% of RBCs. This value in RBCs was converted to an equivalent estimated oral intake of approximately 0.3 mg/day MeHg (300 µg/day MeHg) based on a regression equation using data from Swedish volunteers by Birke et al. (1967)^[[2]](#footnote-3)^: ([RBC ug/kg THg] = 1400 * [oral intake of mg/day MeHg] + 3). Substituting the lowest mercury effect level in RBCs of 400 µg/kg THg (equivalent to 200 µg/L in blood), the regression equation gives an estimated oral intake of 0.284 mg/day MeHg (284 µg/day MeHg), which was presumably rounded up and reported as “about 0.3 mg/day as MeHg” (p.29 & p.271). This per person daily oral PoD was also expressed on a body weight basis as approximately 4 µg/kg-bw/day MeHg for a 70 kg adult (presumably rounded down from 4.29 µg/kg-bw/day MeHg) (Table 14.1 on p.269). The SEG report explicitly identified the lowest adult neurological effect levels (PoDs) in blood, RBCs, hair, and oral intake (Table 14.1, p.268), the recommended safety factor of 10 (p.276), and the derived TRVs (p.277) (see Table S1.1 below).

**Table S1.1 PoDs and TRVs for adult neurotoxicity of MeHg derived by SEG (1971) or inferred by the authors**

| Exposure Metric | PoD | UF | TRV |
| --- | --- | --- | --- |
| hair | **SEG Derived PoD**  **50 µg/g THg ^a^**  (reported on p.25 and p.28 of the SEG report as “the lowest level in the hair” in Niigata poisoning cases, assumed to be based on rounding down from the lowest measured hair level of 52 µg/g THg in case #30 from the Niigata patients reported on p.179 and p.268 of the SEG report) | **10**  (from p.276 of the report, to cover off uncertainties related to “possible genetic, subclinical and other long-term effects” and potential “prenatal lesions”) | **Author Inferred TRV**  **5 µg/g THg ^b^**  (not explicitly derived by SEG, inferred from a hair PoD of 50 µg/g THg ÷ UF10) |
| blood | **SEG Derived PoD**  **200 µg/L THg**  (reported as 0.2 µg/g, lower end of clinical disease from Niigata patients, lowest 3 cases (#17,18,19) had extrapolated day 0 blood levels of ~ 200 – 400 µg/L THg on p. and p.268-269 of the SEG report) |  | **SEG Derived TRV**  **20 µg/L THg**  (reported as 0.02 µg/g on p.277 of the report) |
| RBCs | **SEG Derived PoD**  **400 µg/kg THg**  (reported as 0.4 µg/g, 2x the blood PoD of 200 µg/L THg, reported on p.269 of the SEG report) |  | **SEG Derived TRV**  **40 µg/kg THg**  (reported as 0.04 µg/g on p.277 of the report) |
| oral  (per person per day) | **SEG Derived PoD**  **300 µg/day MeHg**  (reported as 0.3 mg/day, reported on p.269 of the SEG report)  (i.e. from regression equation by Birke et al. (1967) in Figure 11.2 and p.217-219:  [RBC µg/kg THg] = 1400 * [oral intake of mg/day MeHg] + 3  rearranged to:  [oral intake of mg/day MeHg] = ([RBC µg/kg THg] -3) ÷ 1400  when substituted with the lowest effect level in RBCs of 400 µg/kg THg:  [oral intake of mg/day MeHg] = (400 -3) ÷ 1400  [oral intake of mg/day MeHg] = 0.284 mg/day MeHg (**284 µg/day MeHg**)  [oral intake of mg/day MeHg] = ~ 0.3 mg/day MeHg (**300 µg/day MeHg**) |  | **SEG Derived TRV**  **30 µg/day MeHg**  (reported as 0.03 mg/day on p.277 of the report) |
| oral  (per kg body weight per day) | **SEG Derived PoD**  **4 µg/kg-bw/day MeHg**  (for 70 kg adult, reported on p.269 of the SEG report)  for a 70 kg adult:  if using the 284 µg/day MeHg: (284 µg/day) ÷ 70 kg = 4.06 µg/kg-bw/day MeHg  if using the 300 µg/day MeHg: (300 µg/day) ÷ 70 kg = 4.29 µg/kg-bw/day MeHg)  both values can be rounded down to ~4 µg/kg-bw/day MeHg |  | **SEG Derived TRV**  **0.4 µg/kg-bw/day MeHg**  (for 70 kg adult, reported on p.277 of the report) |

^a^ the PoD in hair was also reported as 60 µg/g THg in Table 14.1 of p.269 and p.290 of the SEG report

^b^ also reported as a TRV in hair of 6 µg/g THg on p.30, p.277, and p.290 of the SEG report

# JECFA (1972-1989)

The Joint FAO/WHO Expert Committee on Food Additives (JECFA) concluded at its 16^th^ meeting that *“The lowest mercury levels associated with the onset of clinical disease have been reported to be 50 µg/g in hair and 0.2 µg/g in whole blood* ^[[3]](#footnote-4)^*, corresponding to 0.4 µg/g in blood cells. These levels would seem to represent individuals on the lower end of a distribution curve of methylmercury levels in blood and hair in the population exposed in Niigata”* (FAO/WHO, 1972). This conclusion was principally based on data of adult poisoning cases from Niigata, Japan from an unpublished report by Tsubaki (1972) submitted to JECFA (FAO/WHO, 1972). The JECFA committee further determined, based on regression equations by Kojima & Araki (1972) and Birke et al. (1967), that the PoD in tissue biomarkers represented an oral daily intake (PoD) of approximately 300 µg/day^[[4]](#footnote-5)^ MeHg (reported as 0.3 mg/day “mainly as methylmercury compounds”) (FAO/WHO, 1972). Although not explicitly reported by JECFA, this per person daily oral PoD can also be expressed as a per person weekly PoD of 2100 µg/week MeHg (i.e. 300 µg/day * 7 days/week). Based on this estimated oral PoD, the JECFA committee derived the first “provisional” tolerable weekly intake (pTWI) of 300 µg/week THg of which no more than 200 µg/week should be present as MeHg (i.e. two thirds as MeHg) (FAO/WHO, 1972). While not explicitly stated, it is inferred that an uncertainty factor (UF) of 10 was used for derivation of the pTWI (i.e. [2100 µg/week MeHg]/[200 µg/week MeHg] ≈ 10).

At a subsequent meeting of JECFA, the per person pTWIs for THg and MeHg were expressed on a body weight basis for a 60 kg adult and reported as 5.0 µg/kg bw/week THg and 3.3 µg/kg bw/week MeHg (FAO/WHO, 1989). While not explicitly reported by JECFA, the per body weight pTWIs can be also expressed as equivalent TDI of 0.714 µg/kg bw/day THg or 0.476 µg/kg bw/day MeHg, which are frequently cited in secondary sources (Health Canada, 2007). While the JECFA report included some limited data from Minamata Bay of exposed mothers who gave birth to children exhibiting cerebral palsy-like symptoms and severe mental disability, the dose-response was not characterized nor was an effect level selected for sensitive subpopulations, including fetal exposure. Therefore, the JECFA PoDs, pTWIs and pTDIs were considered to apply to the whole population (FAO/WHO, 1972). These PoDs and TRVs for neurological effects in adults were subsequently reaffirmed in the 33^rd^ meeting of JECFA (FAO/WHO, 1989). While JECFA later derived lower PoDs and TRVs for neurodevelopmental effects of prenatal MeHg exposure (see Table 1 of the main paper), the committee continued to state at their 67^th^ meeting that the earlier derived PoDs and TRVs remained “adequate to take account of neurotoxicity” in adults (FAO/WHO, 2007).

**Table S1.2 PoDs and TRVs for adult neurotoxicity of MeHg derived by JECFA (1972) or inferred by the authors**

| Exposure Metric | PoD | UF | TRV |
| --- | --- | --- | --- |
| hair | **JECFA Derived PoD**  **50 µg/g THg**  (lower end of clinical disease from Niigata patients) | **10**  (inferred from the ratio between the per person weekly PoD of 2100 µg/week MeHg and the reported rounded pTWI of 200 µg/week MeHg, ratio ≈ 10) | **Author Inferred TRV**  **5 µg/g THg**  (not explicitly derived by JECFA, inferred from the hair PoD of 50 µg/g THg ÷ UF10) |
| blood | **JECFA Derived PoD**  **200 µg/L THg**  (lower end of clinical disease from Niigata patients) |  | **Author Inferred TRV**  **20 µg/L THg**  (not explicitly derived by JECFA, inferred from the blood PoD of 200 µg/L THg ÷ UF10) |
| RBCs | **JECFA Derived PoD**  **400 µg/kg THg**  (reported as 0.4 µg/g, or 2x the blood PoD of 200 µg/L THg) |  | **Author Inferred TRV**  **40 µg/kg THg**  (not explicitly derived by JECFA, inferred from the RBC PoD of 400 µg/kg THg ÷ UF10) |
| oral  (per person per day) | **JECFA Derived PoD**  **300 µg/day MeHg**  (reported as 0.3 mg/day; it is inferred by the authors of this manuscript that the PoD of ~300 µg/day was a rounded average from values of 284 and 322 µg/day calculated from the two regression equations below:  1. Birke et al. (1967) for hair THg:  [RBC µg/g THg] = 1.4 * [oral intake of mg/day MeHg] +0.003  rearranged to:  [oral intake of mg/day MeHg] = ([RBC µg/g THg] -0.003) ÷ 1.4  when substituted with the PoD in RBCs of 0.4 µg/g THg:  [oral intake of mg/day MeHg] = (0.4 -0.003) ÷ 1.4  [oral intake of mg/day MeHg] = 0.284 mg/day or **284 µg/day MeHg**)  2. Kojima & Araki (1972) for RBC THg:  [hair µg/g THg] = 150*[intake mg/day MeHg] + 1.66  rearranged to:  [intake mg/day MeHg] = ([hair µg/g THg] – 1.66) ÷ 150  when substituted with the hair PoD of 50 µg/g THg:  [intake mg/day MeHg] = [50 – 1.66] ÷ 150 = 0.322 mg/day or **322 µg/day MeHg** |  | **Author Inferred TRV**  **30 µg/day MeHg**  (not explicitly derived by JECFA, inferred from the RBC PoD of 300 µg/day THg ÷ UF10) |
| oral  (per person per week) | **Author Inferred PoD**  **2100 µg/week MeHg**  (not explicitly derived by JECFA; the authors of this manuscript inferred this PoD as derived from the per person daily PoD: [300 µg/day MeHg]*[7days/week] = 2100 µg/week MeHg) |  | **JECFA Derived TRV**  **200 µg/week MeHg** ^a^  (JECFA reported a pTWI (FAO/WHO, 1972), presumably rounded from the more precise per person weekly pTWI of 210 µg/week MeHg derived directly from the weekly PoD: [2100 µg/week MeHg] ÷ UF10 = 210 µg/week ≈ 200 µg/week rounded)  **Author Inferred TRV**  **210 µg/week MeHg** ^a^  (a more precise per person pTWI if derived directly from the per person PoD: [300 µg/day MeHg]*[7days/week] ÷ UF10 = 210 µg/week) |
| oral  (per kg body weight per week) | **Author Inferred PoD**  **35 µg/kg bw/week MeHg**  (not explicitly derived by JECFA; the authors of this manuscript inferred this PoD as derived from the per person weekly PoD: [2100 µg/week MeHg] ÷ 60kg = [7days/week] = 35 µg/kg bw/week MeHg) |  | **JECFA Derived TRV**  **3.3 µg/kg bw/week MeHg** ^a^  (JECFA reported a pTWI (FAO/WHO, 1989) presumably derived from the rounded pTWI: [200 µg/week MeHg] ÷ 60kg = 3.3 µg/kg bw/week)  **Author Inferred TRV**  **3.5 µg/kg bw/week MeHg** ^a^  (a more precise per body weight pTWI if derived directly from the per person PoD: [300 µg/day MeHg]*[7days/week] ÷ 60kg = 3.5 µg/kg bw/week) |
| oral  (per kg body weight per day) | **Author Inferred PoD**  **5 µg/kg bw/day MeHg**  (the per body weight daily PoD was not explicitly stated by JECFA but can be derived from the reported daily per person PoD of ~300 µg/day MeHg and using the default body weight of 60kg used by JECFA at that time: [300 µg/day] ÷ 60kg = 5.0 µg/kg bw/day MeHg)  Note: this corresponds to a blood to oral conversion factor (**CF**) of 0.025 where: [PoD MB µg/L]***CF** = [PoD oral µg/kg bw/day]  or  **CF** = [PoD oral µg/kg bw/day] ÷ [PoD MB µg/L]  **CF** = [5.0 µg/kg bw/day] ÷ [200 µg/L]  **CF = 0.025** |  | **Author Inferred TRV**  **0.5** **µg/kg bw/day MeHg** ^a^  (a per body weight pTDI was not explicitly derived by JECFA but could be derived directly from the per person per day PoD and using an adult body weight of 60kg:  [300 µg/day] ÷ 60kg ÷ UF10 = 0.5 µg/kg bw/day MeHg) |

^a^ The JECFA did not explicitly report a daily per kg body weight pTDI, however a pTDI of 0.47 µg/kg bw/day MeHg or sometimes 0.476 µg/kg bw/day MeHg is sometimes reported as being attributed to JECFA (Feeley & Lo, 1998; Health Canada, 2007) via derivation from JECFA’s rounded weekly per person pTWI of 200 µg/week (assumed to be rounded down from 210 µg/week) or weekly per kg body weight pTWI of 3.3 µg/kg bw/week MeHg (assumed to be rounded down from 3.5 µg/kg bw/week MeHg). However a more precise TRV can be inferred by directly derivation from JECFA’s per person daily PoD of 300 µg/day MeHg with a UF of 10 and 60kg body weight.– to avoid confusion these unrounded weekly TRVs and more precise directly-derived daily TRV inferred by the authors are also provided in the table.

# WHO IPCS (1976-1990)

In 1976, the World Health Organization International Programme on Chemical Safety (WHO IPCS) followed up on the 1972 JECFA review to include more detailed analyses of data for adult poisoning cases from Niigata, Japan, multiple publications from the Iraq poisoning, as well as data on fish-eating populations with high dietary MeHg exposure from Sweden, Samoa, and Peru (WHO, 1976). A quantitative reanalysis of adult poisoning cases from Niigata (SEG, 1971) and multiple dose-response case series from Iraq (Al-Mufti et al., 1976; Al-Shahristani et al., 1976; Bakir et al., 1973) were used to estimate biomarker and oral intake PoDs (Table 4 in the WHO 1976 report). The WHO reported PoDs ranging from 200-500 µg/L THg in blood, 50-125 µg/g THg in hair, and long-term daily intakes of 3-7 µg/kg-bw/day MeHg^[[5]](#footnote-6)^ (Table 6 in the WHO 1976 report). These PoDs were expected to be *“associated with the earliest effects in the most sensitive group in the adult population”* corresponding to an approximate prevalence of 5% of in a population with exposure at the PoD experiencing paresthesia (burning, tingling of extremities). Conversion from the lower range blood PoD of 200 µg/L THg to oral intake range of 3-7 µg/kg-bw/day MeHg was based on the most conservative coefficient from a series of regression equations in Table 3 of the WHO 1976 report corresponding to a conversion factor (CF) of 0.0143 (i.e. blood*CF=intake, see Table S1.3 below). These blood, hair, and oral intake PoDs were in agreement with those from the JECFA 1972 evaluation and similarly, were considered to apply only to neurological effects in adults. The WHO 1976 review also considered epidemiological data for infants exposed to MeHg in utero from Minamata Bay, Iraq, and other case reports suggesting fetal development may represent a more sensitive period of exposure. However, similar to the JECFA 1972 evaluation, the WHO IPCS considered these data to be too preliminary to quantify the potential risks to children from prenatal exposure to MeHg (WHO, 1976). A subsequent update by the WHO IPCS in 1990 used more recently published data from the Iraq poisoning incident, which concluded that *“The general population does not face a significant health risk from methylmercury. Certain groups with a high fish consumption may attain a blood methylmercury level (about 200 µg/L, corresponding to 50 µg/g of hair) associated with a low (5%) risk of neurological damage to adults.”* (WHO, 1990).

**Table S1.3 PoDs and TRVs for adult neurotoxicity of MeHg derived by the WHO IPCS (1976)**

| Exposure Metric | PoD | UF | TRV |
| --- | --- | --- | --- |
| hair | **WHO Derived PoD**  **50 µg/g THg** | **N/A**  (WHO did not select a UF for derivation of a TRV) | **N/A**  (WHO did not derive a TRV) |
| blood | **WHO Derived PoD**  **200 µg/L THg** |  |  |
| oral  (per kg body weight per day) | **WHO Derived PoD**  **range: 3 - 7 µg/kg-bw/day MeHg**  (i.e. estimated from the range of blood PoD 200 – 500 µg/L THg using regression equation from Table 3 of WHO (1976):  (blood conc. µg/L THg) = intake (MeHg µg per 70 kg person/day)*(beta)  rearranged to:  intake (MeHg µg/70 kg person/day) = (blood conc. µg/L THg) ÷ (beta)  where beta was 1.0 (the most conservative beta ranging from 0.3 – 1.0), and the estimated intake is then multiplied by 70 kg-bw to express per body weight (µg/kg-bw/day MeHg):  intake = (blood conc. µg/L THg) ÷ [(beta)*(70 kg)]  intake = (200 to 500 µg/L THg) ÷ [(1.0)*(70 kg)]  rearranged to:  intake = (200 to 500 µg/L THg) * (0.0143) ^a^  intake = 2.9 to 7.1 µg/kg-bw/day MeHg  intake = 3 – 7 µg/kg-bw/day MeHg (rounded) |  |  |

^a^ (blood ug/L THg) ÷ [(1.0)*(70kg)] = (blood ug/L) * (1/70) = (blood ug/L) * (0.0143) where 0.0143 corresponds to a conversion factor (CF) from blood to oral intake: intake (ug/kg-bw/day MeHg) = (blood ug/L THg) * CF

# Government of Canada (1971 – 2010)

Since the early 1970’s various programs of Health Canada (formerly the Department of National Health and Welfare, or simply Health and Welfare Canada) and other Departments including Indigenous Services Canada have considered the potential health effects of oral MeHg exposure from food and drinking water.

## Health Canada’s Bureau of Chemical Safety (BCS) (1971 – 2010) - retail foods

Since the early 1970’s various programs of Health Canada have considered the potential health effects of MeHg exposure from retail food (Health & Welfare Canada, 1971), the consumption of fish in Indigenous populations (Health & Welfare Canada, 1979), and drinking water (Health Canada, 1986). This section and Table A3 describes the historical basis of Health Canada’s BCS PoDs and TRVs for adult neurotoxicity used for risk assessment of MeHg exposure from retail food.

As early as 1971, conference presentations by the Food Advisory Bureau of the Food and Drug Directorate of Health and Welfare Canada (now Health Canada’s BCS), provided a review of the draft findings of the SEG (1971), including a blood PoD of 200 µg/L THg reported as the approximate lower level of neurological effects in adult poisoning cases from Japan (Health & Welfare Canada, 1971). While a TRV was not derived, these data were used collectively to support continued application of the Canadian temporary “administrative tolerance” or guideline for mercury in retail fish of 0.5 ppm wet weight (Health & Welfare Canada, 1971). In 1972, JECFA’s hazard opinion on MeHg recommended a daily PoD of 300 µg/day MeHg and derived rounded weekly pTWI’s of 200 µg/week MeHg or 3.3 µg/kg bw/week MeHg for a 60 kg adult (FAO/WHO, 1972) and later that year Health and Welfare Canada started applying the JECFA opinion for assessing risks from MeHg exposure in retail fish (Health & Welfare Canada, 1972). Health Canada’s BCS has continued to employ the JECFA TRV, but it was expressed in later assessments as an oral TDI of 0.47 µg/kg bw/day MeHg for a 60 kg adult (Table S1.4 below) (Feeley & Lo, 1998; Health Canada, 2007; Legrand et al., 2010). The reported JECFA weekly pTWIs of 200 µg/week or 3.3 µg/kg bw/week MeHg were likely rounded down from the more precise pTWIs of 210 µg/week and 3.5 µg/kg bw/week MeHg and therefore, an alternative oral TRV of 0.5 µg/kg bw/day MeHg can be derived using JECFA’s unrounded PoDs or TWIs (see footnote b in Table S1.4 below).

**Table S1.4 PoDs and TRVs for adult neurotoxicity of MeHg used by Health Canada’s BCS for risk assessment of MeHg in retail food**

| Exposure Metric | PoD | UF | TRV |
| --- | --- | --- | --- |
| hair | **Author Inferred PoD**  **50 µg/g THg**  (This hair PoD was selected by the SEG (1971) and JECFA (1972). Since the JECFA opinion was adopted in 1972 by Health Canada’s former Food Advisory Bureau (Health & Welfare Canada, 1972), it is inferred that the hair PoD of 50 µg/g THg was also adopted and has continued to be used by Health Canada’s BCS. Coincidentally the same hair PoD can be converted from the blood PoD of 200 µg/L THg (below) using the hair:blood ratio of 0.250:1 as recommended for all populations in Legrand et al. (2010).) | **10**  The “safety factor” of 10 recommended by the SEG (1971) was used by JECFA in derivation of their pTWI (FAO/WHO, 1972). Since the JECFA opinion was adopted in 1972 by Health Canada’s former Food Advisory Bureau (Health & Welfare Canada, 1972), and UF10 was reported in Feeley & Lo (1998), it is inferred that the UF10 was also adopted by Health Canada’s BCS. | **Author Inferred TRV**  **5 µg/g THg** ^a^  (Not explicitly derived by Health Canada/s BCS but can be calculated directly from the hair PoD of 50 µg/g THg ¸ UF10 = hair TRV of 5 µg/g THg; alternatively the same hair TRV can be derived from the blood “guidance value” of 20 µg/L THg below using a hair:blood ratio of 0.250:1.) |
| blood | **BCS Derived TRV**  **200 µg/L THg**  (This blood PoD was selected by the SEG (1971) and JECFA (1972). Since the JECFA opinion was adopted in 1972 by Health Canada’s former Food Advisory Bureau (Health & Welfare Canada, 1972), and was reported in Feeley & Lo (1998) as a LOAEL for derivation of the pTDI) |  | **Author Inferred TRV**  **20 µg/L THg**  (Reported as a blood “guidance value” in Legrand et al. (2010).) |
| oral  (per person per day) | **Author Inferred TRV**  **300** **µg/day MeHg**  (This oral PoD was selected by the SEG (1971) and JECFA (1972). Since the JECFA opinion was adopted in 1972 by Health Canada’s former Food Advisory Bureau (Health & Welfare Canada, 1972), it is inferred that the oral PoD of 300 µg/day MeHg was also adopted and has continued to be used by Health Canada’s BCS) |  | **BCS Derived TRV**  **30 µg/day MeHg**  (Reported as a per person pTDI in Feeley & Lo (1998), i.e. assumed that this oral PoD was used for derivation of the BCS TRV of 30 µg/day MeHg reported in Feeley & Lo (1998): PoD of 300 µg/day MeHg ÷ UF10) |
| oral  (per kg body weight per day) | **Author Inferred TRV**  **5.0 µg/kg bw/day MeHg**  (Not explicitly derived by either SEG (1971) or JECFA (1972) but can be calculated from the JECFA daily per person PoD assuming a 60kg adult:  [300 µg/day MeHg] ÷ 60kg  = 5.0 µg/kg bw/day MeHg) |  | **BCS Derived TRV**  **0.47 µg/kg bw/day MeHg**  (Reported as a pTDI by Health Canada’s BCS (Health Canada, 1997, 2007; Legrand et al., 2010) calculated from JECFA’s rounded pTWI of 3.3 µg/kg bw/week ÷ 7days/week = 0.47 µg/kg bw/day ^b^ )  **Author Inferred TRV**  **0.5** **µg/kg bw/day MeHg**  (To align with similar notation in the JECFA Table S1.2, an alternative daily per body weight TRV can also be derived directly from JECFA’s daily per person PoD and unrounded TWIs ^c^ .) |

^a^ A hair TRV of 6 µg/g THg rather than 5 µg/g has historically been used by the Medical Services Branch of Health Canada (Health & Welfare Canada, 1979) and later Indigenous Services Canada (Tikhonov et al., 2021). The reason for this difference is that the Medical Services Branch initially only derived a blood TRV of 20 µg/L THg and used a hair:blood conversion of 0.300:1 to derive the hair TRV of 6 µg/g THg.

^b^ The reported pTDI of 0.47 µg/kg bw/day was calculated from the JECFA’s rounded TWI of 3.3 µg/kg bw/week, when ÷ 7days/week gives the reported pTDI of 0.47 µg/kg bw/day. However, unrounded JECFA TWIs can be calculated directly from JECFA’s oral PoD of 300 µg/day MeHg as follows: JECFA daily PoD of 300 µg/day ÷ UF10 = oral daily TDI of 30 µg/day, and unrounded weekly TWIs can be calculated as: daily TDI of 30 µg/day * 7 days/week = unrounded weekly TWIs of 210 µg/week or 3.5 µg/kg bw/week for a 60 kg adult

^c^ An alternative oral TDI of 0.5 µg/kg bw/day MeHg can be derived more directly from JECFAs unrounded daily PoD (i.e. daily PoD of 300 µg/day MeHg ÷ UF10 ÷ 60kg = TDI of 0.5 µg/kg bw/day MeHg) or unrounded weekly TWI i.e. unrounded weekly TWI of 210 µg/week MeHg ÷ 7 days/week ÷ 60kg = TDI of 0.5 µg/kg bw/day MeHg. See Table S1.2 for additional explanation of JECFA’s PoDs and TRVs.

## Indigenous Services Canada - Exposure to MeHg through country foods^[[6]](#footnote-7)^

In 1979, a report by the Medical Services Branch of Health and Welfare Canada (now Health and Social Sector of the Indigenous Services Canada)^[[7]](#footnote-8)^ synthesized the findings from the SEG (1971), JECFA (1972), and WHO (1976). Based on adverse effects in adults from the Japanese and Iraq poisonings incidents, they selected a PoD in blood of 200 µg/L THg (Health & Welfare Canada, 1979). After applying the safety factor of 10 as recommended by the SEG (1971), the Medical Services Branch derived a blood “guideline” of 20 µg/L THg. levels below this guideline as representing the “normal acceptable range” (Health & Welfare Canada, 1979; Legrand et al., 2010). However, for the purposes of public health risk management and risk communication, blood THg concentrations above 100 μg/L were classified as “at risk” (Health & Welfare Canada, 1979; Legrand et al., 2010). Using a hair_ppb_:blood_ppb_ ratio of 300:1 (i.e. hair_ppm_:blood_ppb_ ratio of 0.300:1), the blood PoD of 200 µg/L , “at risk” blood level of 100 µg/L, and blood TRV of 20 µg/L THg correspond to an equivalent hair PoD of 60 µg/g, hair “at risk” level of 30 µg/g, and hair TRV of 6 µg/g THg, respectively (Health & Welfare Canada, 1979; Health Canada, 1999). A later publication recommended using a more commonly applied hair_ppb_:blood_ppb_ ratio of 250:1 (i.e. hair_ppm_:blood_ppb_ ratio of 0.250:1), which yields corresponding hair THg values of 50 µg/g (PoD), 25 µg/g (“at risk”) and 5 µg/g (TRV) (Legrand et al., 2010). Notably, the hair PoD of 50 µg/g THg was also originally selected by the SEG (1971), JECFA (FAO/WHO, 1972), WHO (1976), and later Health Canada (1999), based directly on measured hair THg levels in adult poisoning cases in Japan. Coincidentally, this value also results from converting the blood PoD of 200 µg/L THg using a hair_ppb_:blood_ppb_ ratio of 250:1 (i.e. hair_ppm_:blood_ppb_ ratio of 0.250:1). Regardless, both the 300:1 and 250:1 ratios are considered reasonable point estimates and yield similar population-level conversions between hair and blood THg (Singh et al. 2023). For historical consistency, Indigenous Services Canada continues to employ the hair_ppb_:blood_ppb_ ratio of 300:1 and the associated hair “at risk” level and hair TRV of 30 and 6 µg/g THg, respectively, for public health management of MeHg exposure in Indigenous people (excluding children and women of childbearing age) in Canada through country food consumption (Tikhonov et al., 2021). As discussed in Section 2.5 of the main paper, a more conservative lower blood guideline level of 8 μg/L is applied to children and women of childbearing age to protect against potential neurodevelopmental effects of dietary MeHg in country foods (Legrand et al. 2010; Tikhonov et al., 2021).

## Other Health Canada programs – drinking water

Health Canada reviewed the available epidemiological and toxicity data on mercury in order to set a drinking water guideline (Health Canada, 1986). In this review, data from adult MeHg poisoning cases were used to selected PoDs in blood (200 µg/L THg), hair (50 µg/g THg), and oral intake (300 µg/day MeHg). These PoDs were based on a contract report for the WHO (Piotrowski & Inskip, 1981), which was an update to the previous findings from the SEG (1971), JECFA (1972), and WHO (1976).

# US EPA (1985)

The United States Environmental Protection Agency’s (US EPA) oral reference dose (RfD) for adult neurotoxicity of MeHg was established at a consensus meeting of the Integrated Risk Information System (IRIS) Work Group on December 2, 1985 and was briefly summarized in a 1987 IRIS Chemical File (US EPA, 1987) and in Section 4.1.1.1 of the US EPA Water Quality Criterion for the Protection of Human Health (US EPA, 2001b). The principal supporting study was the 1976 evaluation by the WHO IPCS (1976) that reported the “earliest effects in man” were at blood concentrations between 200 – 500 µg/L (reported as “ng Hg/ml”) and represented “both pre- and postnatal exposures” and were “equivalent to intakes in the range of 3-7 µg/kg-bw/day”. The LOAEL was “associated with CNS effects such as ataxia, paresthesia, etc.” (US EPA, 1987). The US EPA selected as PoDs the lower end of these blood and oral intake levels corresponding to 200 µg/L THg and 3 µg/kg-bw/day MeHg respectively. The US EPA applied a UF of 10 to account for the use of a lowest observed adverse effect level (LOAEL) vs. no-observed adverse effect level (NOAEL), but considered that an additional UF was unnecessary “since the effects are seen in sensitive individuals”. An oral RfD of 0.3 µg/kg-bw/day MeHg was derived and constituted the agency’s TRV for adult neurological effects of MeHg until subsequent opinions, which focussed on neurodevelopmental effects in children exposed prenatally (US EPA, 1997, 2001a, 2001b).

# US FDA (1986)

In 1986, a review by the US Food & Drug Administration (US FDA) determined that the “threshold value at which symptoms of toxicity associated with MeHg are first noticeable” was 200 µg/L THg in blood and 50 µg/g THg in hair (Tollefson & Cordle, 1986). This conclusion was based principally on data from the Japanese poisoning cases in adults as analyzed by the SEG (1971) and JECFA (1972), which stated that the “lowest blood level for the appearance of signs and symptoms of methylmercury poisoning was 200 ppb (0.2 ppm)” (i.e. 200 µg/L blood THg). Using a dose-response relationship for fish consumers in Sweden (Skerfving, 1974), a steady state blood concentration of 200 µg/L THg was converted to a daily intake of 300 µg/day MeHg, equivalent to a daily intake of 4.28 µg/kg-bw/day MeHg for a 70 kg adult (Tollefson & Cordle, 1986). Considering the uncertainties in the dataset, including mercury-selenium interactions and the sensitivity of the developing fetus, the US FDA applied a safety factor of 10 to arrive at a “maximum tolerable level” or acceptable daily intake (ADI) of 30 µg/day MeHg, which is equivalent to 0.43 µg/kg-bw/day MeHg for a 70 kg adult. The blood and hair concentrations corresponding to the ADI could be expressed as 20 µg/L THg in blood and 5 µg/g THg in hair respectively (Tollefson & Cordle, 1986). The ADI and blood/hair guideline levels derived by the US FDA were based on adverse neurological effects in adults who were exposed to very high levels of mercury from contamination incidents. However, the application of a larger safety factor of 10 was considered to account for potential sensitivity of the fetus and therefore, the ADI was considered to be protective of both the general population and women of childbearing age (WCBA) (Tollefson & Cordle, 1986). The US FDA has subsequently updated its hazard characterization for prenatal MeHg exposure and has since employed a different risk assessment approach to protect against potential neurodevelopmental effects (US FDA, 2014).

# References

Al-Mufti, A. W., Copplestone, J. F., Kazantzis, G., Mahmoud, R. M., & Majid, M. A. (1976). Epidemiology of organomercury poisoning in Iraq. I. Incidence in a defined area and relationship to the eating of contaminated bread. *Bulletin of the World Health Organization*, *53 Suppl*(Suppl), 23–36. https://pmc.ncbi.nlm.nih.gov/articles/PMC2366407/

Al-Shahristani, H., Shihab, K., & Al-Haddad, I. K. (1976). Mercury in hair as an indicator of total body burden. *Bulletin of the World Health Organization*, *53 Suppl*(Suppl), 105–112. https://pmc.ncbi.nlm.nih.gov/articles/PMC2366395/

Bakir, F., Damluji, S. F., Amin-Zaki, L., Murtadha, M., Khalidi, A., al-Rawi, N. Y., Tikriti, S., Dahahir, H. I., Clarkson, T. W., Smith, J. C., & Doherty, R. A. (1973). Methylmercury poisoning in Iraq. *Science (New York, N.Y.)*, *181*(4096), 230–241. https://doi.org/10.1126/science.181.4096.230

Berlin, M., Ramel, C., & Swensson, A. (1969). *Poisoning by the consumption of fish containing a methyl mercury compound. A report from a study trip to Japan in 1968. (Swedish) - cited in Swedish Expert Group, 1971.*

Birke, G., Johnels, A., Plantin, L., Sjostrand, B., & Westermark, T. (1967). Mercury poisoning through eating fish? *Lakartidningen*, *64*, 3628–3637.

FAO/WHO. (1972). *Mercury. Evaluation of mercury, lead, cadmium and the food additives amaranth, diethylpyrocarbonate, and octyl gallate. (FAS 4) WHO Food Additives Series, No. 4, 1972, nos 249-256. 16th Meeting of the Joint FAO/WHO Expert Committee on Food Additives which*. https://inchem.org/documents/jecfa/jecmono/v004je02.htm

FAO/WHO. (1989). *Methylmercury. Toxicological evaluation of certain food additives and contaminants. (FAS 24) WHO Food Additives Series, No. 24. Cambridge University Press, 1989, nos 651-664 (Thirty-third report of the Joint FAO/WHO Expert Committee on Food Additives).* https://inchem.org/documents/jecfa/jecmono/v024je12.htm

FAO/WHO. (2007). *Methylmercury (addendum): Safety evaluation of certain food additives and contaminants. (FAS 58) WHO Food Additive Series 58. Prepared by the Sixty-seventh meeting of the Joint FAO/WHO Expert Committee on Food Additives (JECFA)*. https://apps.who.int/iris/bitstream/handle/10665/43645/9789241660587_eng.pdf;sequence=1

Feeley, M., & Lo, M.-T. (1998). Risk Assessment for Mercury in Health Canada - Development of the Provisional Tolerable Daily Intake (pTDI) Value. In W. Pilgrim, N. Burgess, & M.-F. Giguere (Eds.), *Conference Proceedings. Mercury in Eastern Canada and the Northeast States. New Brunswick, September 21-23, 1998. Ecological Monitoring and Assessment Network (EMAN), Environment Canada-Atlantic Region, Health Canada and New Brunswick Dept. of the Env’t.* (p. 32). https://hawcproject.org/media/study-attachment/Feeley__Lo_1998_-_Health_Canada_pTDI_for_MeHg.pdf

Health & Welfare Canada. (1971). The Canadian approach to acceptable daily intakes of mercury in foods. (A.B. Morrison, Food & Drug Directorate, Department of National Health and Welfare, Ottawa). Special Symposium on Mercury in Man’s Environment. Feb. 15-16, 1971. In J. Watkins (Ed.), *Proceedings of the Royal Society of Canada* (pp. 157–164). https://search.worldcat.org/title/15773759?oclcNum=15773759

Health & Welfare Canada. (1972). *Mercury in Fish. Letter from the Food Advisory Bureau, Health Protection Branch, Department of National Health & Welfare to the Wellington-Dufferin-Guelph Health Unit, Guelph, Ontario . Dec. 1, 1972. (unpublished)*.

Health & Welfare Canada. (1979). *Methylmercury in Canada: exposure of Indian and Inuit residents to methylmercury in the Canadian environment. A review of the Medical Services Branch, Department of National Health & Welfare, Mercury Program Findings to December 31, 1978.*

Health Canada. (1986). *Mercury. Guidelines for Canadian Drinking Water Quality: Guideline Technical Document - Mercury. April 1979 (Updated September 1986)*. https://www.canada.ca/en/health-canada/services/publications/healthy-living/guidelines-canadian-drinking-water-quality-guideline-technical-document-mercury.html#a12

Health Canada. (1997). *pTDI of methylmercury (MeHg) for pregnant women and infants - rationale. Bureau of Chemical Safety. (unpublished)*.

Health Canada. (1999). *Methylmercury in Canada: exposure of Indian and Inuit residents to methylmercury. Volume. 3. A review of the Medical Services Branch, Health Canada Program Findings to December 31, 1996.*

Health Canada. (2007). *Human Health Risk Assessment of Mercury in Fish and Health Benefits of Fish Consumption. (Bureau of Chemical Safety, Food Directorate, Health Products and Food Branch)*. https://www.canada.ca/content/dam/hc-sc/migration/hc-sc/fn-an/alt_formats/hpfb-dgpsa/pdf/nutrition/merc_fish_poisson-eng.pdf

Health Canada. (2023). *Guidance for Evaluating Human Health Effects in Impact Assessment: Country Foods.* https://publications.gc.ca/collections/collection_2024/sc-hc/H129-54-5-2023-eng.pdf

Kojima, K., & Araki, T. (1972). *Paper submitted to WHO (unpublished, cited in FAO-WHO 1972)*.

Legrand, M., Feeley, M., Tikhonov, C., Schoen, D., & Li-Muller, A. (2010). Methylmercury blood guidance values for Canada. *Canadian Journal of Public Health*, *101*(1), 28–31. https://doi.org/10.1007/bf03405557

Piotrowski, J., & Inskip, M. (1981). *Health Effects of Methylmercury, MARC Report No. 24, Monitoring and Assessment Research Centre (MARC), Chelsea College, University of London.* https://stg-wedocs.unep.org/bitstream/handle/20.500.11822/28017/MARC_RprtNo24.pdf?sequence=1&isAllowed=y

SEG. (1971). Methyl mercury in fish : a toxicologic-epidemiologic evaluation of risks : report from an expert group. In *Nordisk Hygienisk Tidskrift: Vol. Supplement*. https://hawcproject.org/media/study-attachment/Swedish_Expert_Group_1971.pdf

Skerfving, S. (1974). Methylmercury exposure, mercury levels in blood and hair, and health status in Swedes consuming contaminated fish. *Toxicology*, *2*(1), 3–23. https://doi.org/https://doi.org/10.1016/0300-483X(74)90038-9

Tikhonov, C., Schwartz, H., Marushka, L., Chan, H. M., Batal, M., Sadik, T., Ing, A., & Fediuk, K. (2021). Regionally representative hair mercury levels in Canadian First Nations adults living on reserves. *Canadian Journal of Public Health*, *112*(1), 97–112. https://doi.org/10.17269/s41997-021-00508-5

Tollefson, L., & Cordle, F. (1986). Methylmercury in fish: a review of residue levels, fish consumption and regulatory action in the United States. *Environmental Health Perspectives*, *68*, 203–208. https://doi.org/10.1289/ehp.8668203

Tsubaki, T. (1972). *Paper submitted to WHO (unpublished, cited in FAO-WHO 1972)*.

US EPA. (1971). *Mercury in the Environment: A Toxicological and Epidemiological Appraisal. (US EPA Contract No. CPA 70-30, prepared by Friberg L. and Vostal J. (editors) of the Karolinska Institute)*. https://nepis.epa.gov/Exe/ZyPDF.cgi/9101G15H.PDF?Dockey=9101G15H.PDF

US EPA. (1987). *Methyl Mercury (CAS No. 22967-92-6). Revised 11/16/1986. Integrated Risk Infomration System Chemical Files Volume II. EPA/600/8-86/032b. March 1987.* https://nepis.epa.gov/Exe/ZyPDF.cgi/2000G6KS.PDF?Dockey=2000G6KS.PDF

US EPA. (1997). Volume V: Health Effects of Mercury and Mercury Compounds. Mercury Study Report to Congress. December 1997 (EPA-452/R-97-003) Office of Air Quality Planning and Standards, Office of Research and Development, U.S. Environmental Protection Agency. In *Mercury Study Report to Congress*. https://www.epa.gov/mercury/mercury-study-report-congress

US EPA. (2001a). Methylmercury (MeHg) (CASRN 22967-92-6). I. Chronic Health Hazard Assessments for Noncarcinogenic Effects. I.A. Reference Dose for Chronic Oral Exposure (RfD). Last Revised 07/27/2001. Integrated Risk Information System (IRIS). Chemical Assessment Summary. In *Integrated Risk Information System (IRIS) Chemical Assessment Summary National Center for Environmental Assessment*. https://iris.epa.gov/static/pdfs/0073_summary.pdf

US EPA. (2001b). *Water Quality Criterion for the Protection of Human Health: Methylmercury. Final. (January 2001, EPA 823-R-01-001). Office of Science and Technology Office of Water U.S. Environmental Protection Agency Washington, DC 20460*. https://doi.org/https://www.epa.gov/sites/default/files/2020-01/documents/methylmercury-criterion-2001.pdf

US FDA. (2014). *Quantitative Assessment of the Net Effects on Fetal Neurodevelopment from Eating Commercial Fish (As Measured by IQ and also by Early Age Verbal Development in Children).* https://www.fda.gov/food/metals-and-your-food/quantitative-assessment-net-effects-fetal-neurodevelopment-eating-commercial-fish-measured-iq-and

WHO. (1976). *Environmental Health Criteria 1: Mercury. International Programme on Chemical Safety (IPCS).* https://inchem.org/documents/ehc/ehc/ehc001.htm

WHO. (1990). *Environmental Health Criteria 101: Methylmercury. International Programme on Chemical Safety (IPCS).* https://inchem.org/documents/ehc/ehc/ehc101.htm

1. The 3 Niigata cases (#17, #18, #19) with the lowest extrapolated day 0 blood concentrations (from 200 – 400 µg/L THg) would have had estimated hair levels of 50 – 100 µg/g THg based on a hair:blood ratio of 0.250:1. However, these 3 cases were reported to have much higher measured hair levels of 210 - 280 µg/g THg at 0-43 days after symptom onset (Table 9.7 on p.176). This mismatch of estimated day 0 blood and hair mercury concentrations 0-43 days after symptom onset were not discussed by the Swedish Expert Group. In contrast, if hair measures were considered more reliable estimates of MeHg exposure when symptoms were first reported, then these “lowest” or most sensitive 3 cases would give corresponding blood levels of ~800 – 1100 µg/L using the hair:blood ratio of 0.250:1 vs the estimated extrapolated day 0 blood concentrations from 200 – 400 µg/L THg from Figure 9.1 (p.170). [↑](#footnote-ref-2)
2. in Figure 11.2 and p.217-219 of the SEG report [↑](#footnote-ref-3)
3. 0.2 µg/g THg in blood is equivalent to 200 µg/L THg [↑](#footnote-ref-4)
4. The oral PoD of 300 µg/day MeHg was presumed to have been estimated from two regression equations: 1. An unpublished report submitted to JECFA (Kojima & Araki, 1972) where [hair µg/g THg] = 150*[intake mg/day MeHg] + 1.66, and 2. Birke et al. (1967) where [RBC µg/g THg] = 1.4 * [oral intake of mg/day MeHg] + 0.003 [↑](#footnote-ref-5)
5. The conversion between blood THg (µg/L) to oral intake MeHg (µg/70kg person/day) in Table 3 of WHO (1976) was based on estimated MeHg daily intake, and therefore, predicted oral PoDs are also in units of MeHg. [↑](#footnote-ref-6)
6. “Country food” or “traditional food” is defined as “any food that is trapped, fished, hunted, harvested or grown for subsistence or medicinal purposes, outside of the commercial food chain.” (Health Canada, 2023) [↑](#footnote-ref-7)
7. Biomonitoring and dietary surveys to assess MeHg exposure in Indigenous communities in Canada continues today under the administration of Indigenous Services Canada (ISC) (Tikhonov et al., 2021), and Crown-Indigenous Relations and Northern Affairs Canada (CIRNAC) via the Northern Contaminants Program (NCP) . Exposures to MeHg from country food continue to be managed through shared responsibilities of governments and health authorities at the Federal, Provincial/Territorial and Indigenous level (Health Canada, 2023). [↑](#footnote-ref-8)
