# Supplementary material for: Neurodevelopmental effects of methylmercury (MeHg): a review of epidemiological points of departure (PoDs), toxicological reference values (TRVs), and key uncertainties in human health risk assessment
Source: Arch Toxicol. 2026 Mar 10;100(6):2191–219. doi: 10.1007/s00204-026-04345-8 (PMC13221417; doi:10.1007/s00204-026-04345-8)
Supplement: Supplementary file 2 — Supplementary file2 (DOCX 118 kb) [file 204_2026_4345_MOESM2_ESM.docx]

**Supplemental File S2.**

**Description of the Cohorts Used for PoD Selection by Risk Assessment Organizations**

*Archives of Toxicology*

Neurodevelopmental effects of methylmercury (MeHg): A review of epidemiological points of departure (PoDs), toxicological reference values (TRVs), and key uncertainties in human health risk assessment

Blechinger, Scott R.^1^* (ORCID 0000-0002-4991-4597, Scopus ID 6506155596)

Singh, Kavita^2^ (ORCID N/A, Scopus ID 58382466900)

Afghan, Abdul^1^ (ORCID N/A, Scopus ID 58522410100)

Smith, Catherine A.^1^ (ORCID N/A, Scopus ID 46461849300)

^1^ Bureau of Chemical Safety, Food and Nutrition Directorate, Health Canada, Ottawa, Canada

^2^ Environmental Health Science and Research Bureau, Health Canada, Ottawa, Canada

*Corresponding author: scott.blechinger@hc-sc.gc.ca

The points of departure (PoDs) in this review were almost exclusively based on the findings from three cohorts: New Zealand Cohort at 6-7 years, Faroe Islands Cohort 1 at 6.8 years, Seychelles Main Cohort at 5.5 and 9 years. The sections that follow include a brief description of each cohort (timeframe, eligibility and recruitment, prenatal Hg biomarker measured), a table reporting information on the regression coefficients for outcome tests assessed, the modelled benchmark dose/benchmark dose lower confidence limit (BMD/BMDL) estimate (where applicable), and whether the regression coefficient was included in the meta-analysis by Axelrad et al. (2007).

# New Zealand Cohort

The New Zealand cohort included mothers who gave birth in one of 25 maternity hospitals from the northern part of the North Island of New Zealand between 1977-1978. All 16,293 eligible mothers were invited to participate, with 10,930 giving consent, completing an initial questionnaire on demographics, lifestyle, and fish consumption habits, and providing a sample of scalp hair that was collected when the mothers were in the post-natal ward of the maternity hospital. Mothers who reported high fish consumption ( >3 fish meals/week; n=935) in the questionnaire were selected for the initial analysis of MH THg (average THg in 1cm segments from a 9cm length). Of the 935 high fish consuming mothers who underwent hair analysis, 73 mothers had an average MH THg of ≥ 6 ug/g (range: 6.0 - 86.4 ug/g). Maternal ethnicity was reported as 65/73 (89%) Polynesian (i.e. 45/73 [62%] Pacific Islander + 20/73 [27%] Māori), and 8/73 (11%) European. The 73 mother-child pairs with high fish consumption during pregnancy and high MH THg were referred to as the "high mercury" or "exposed" group in subsequent analyses for neurodevelopmental outcomes at 4 yrs (Kjellström et al., 1986).

At the 6-7 yr assessment, 57 of the “exposed” mother-child pairs were matched 1:3 to three other groups with lower MH THg concentrations (group 2: hair range 3-5.99 µg/g and “high fish”; group 3: hair range 0.1-2.99 µg/g and “high fish”; group 4: hair range 0.1-2.99 µg/g and “low fish”). There were 228 fully-matched dyads and an additional 10 incompletely matched (n=4 “exposed” + n=6 from other groups) to give a total analysis sample size of 238 mother-child pairs (Kjellström et al., 1989). In the whole sample, the geometric mean (GM) of MH THg was 3.19 µg/g (min= 0.5, p25= 1.67, p75=6.12, max= 86.4 µg/g) (Kjellström et al., 1989). At 6-7 yrs of age, 23 outcome tests were assessed and analyzed for association with MH THg with multivariate linear regression; however, estimates for only 5 of the 23 tests were reported as a change in the test score for a category increase in MH THg (“exposed” from 6-86.4 µg/g vs reference group from 0.1-5.99 µg/g) in Kjellstrom et al. (1989). A reanalysis of the same 5 tests was done by Crump et al. (1998) who reported regression coefficients as a change in test score for a linear increase of 1 µg/g MH THg and also performed BMD modelling using a P(0)=5% background abnormal and benchmark response (BMR)=10% added-risk. The BMD estimates were also included in the National Academy of Sciences (NAS) report (NAS, 2000), but using a BMR=5% extra-risk instead for consistency with the BMD results from the Faroe Islands and the Seychelles (Table S2.1).

**Table S2.1** Regression coefficients and benchmark dose estimates for **23 tests** from the New Zealand Cohort at 6-7 years

| **Avg. child age (years)** | **Regression Coefficient (β = Δ test score for an 🠙 of 1 µg/g MH THg) ^a^** | | | | | | | **Regression coefficient re-scaled in Axelrad et al. (2007) meta-analysis as β_∆IQ_ (SE) ^b^** | | **BMD estimates ^c^ (µg/g MH THg)** | | |
| --- | --- | --- | --- | --- | --- | --- | --- | --- | --- | --- | --- | --- |
|  | **Outcome Tests (n=23 ^d^)** | **β ^e^** | **95% LCL** | **95% UCL** | **SE** | **p ^f^** |  | | **BMD_05_** | | **BMDL_05_** |  |
| 6-7 years | Burt Word Recognition Test ^e^ | NR | NR | NR | NR | >0.10 | . | | . | | . |  |
|  | Clay Diagnostic Survey - concepts | NR | NR | NR | NR | 0.03 | . | | . | | . |  |
|  | Clay Diagnostic Survey – letter test | NR | NR | NR | NR | 0.07 | . | | . | | . |  |
|  | Clay Diagnostic Survey – word test | NR | NR | NR | NR | >0.10 | . | | . | | . |  |
|  | Clay Diagnostic Survey – reading accuracy | NR | NR | NR | NR | >0.10 | . | | . | | . |  |
|  | Clay Diagnostic Survey – reading level | NR | NR | NR | NR | >0.10 | . | | . | | . |  |
|  | Key Math Diagnostic Assessment | NR | NR | NR | NR | >0.10 | . | | . | | . |  |
|  | MSCA – general cognitive | NR | NR | NR | NR | 0.06 | . | | . | | . |  |
|  | MSCA – memory | NR | NR | NR | NR | >0.10 | . | | . | | . |  |
|  | MSCA – motor (ln-transformed) | -0.008 | -0.018 | 0.0023 | NR | >0.10 | . | | 13 | | 6 |  |
|  | MSCA – perceptual | -0.50 | -0.92 | -0.077 | NR | 0.02 | -0.80 (0.315) | | 8 | | 4 |  |
|  | MSCA – quantitative | NR | NR | NR | NR | >0.10 | . | | . | | . |  |
|  | MSCA – verbal reasoning | NR | NR | NR | NR | >0.10 | . | | . | | . |  |
|  | Peabody Picture Vocabulary Test ^e^ | NR | NR | NR | NR | >0.10 | . | | . | | . |  |
|  | TOLD – grammar completion | NR | NR | NR | NR | 0.005 | . | | . | | . |  |
|  | TOLD – grammar understanding | NR | NR | NR | NR | 0.02 | . | | . | | . |  |
|  | TOLD – oral vocabulary | NR | NR | NR | NR | >0.10 | . | | . | | . |  |
|  | TOLD – picture vocabulary | NR | NR | NR | NR | >0.10 | . | | . | | . |  |
|  | TOLD – sentence initiation | NR | NR | NR | NR | >0.10 | . | | . | | . |  |
|  | TOLD – spoken language | -0.42 | -0.98 | 0.13 | NR | >0.10 | -0.56 (0.282) | | 12 | | 6 |  |
|  | WISC R – full-scale IQ | -0.42 | -1.1 | 0.18 | NR | >0.10 | -0.50 (0.268) | | 12 | | 6 |  |
|  | WISC R – performance IQ | -0.47 | -1.1 | 0.16 | NR | >0.10 | -0.51 (0.310) | | 13 | | 6 |  |
|  | WISC R – verbal IQ | NR | NR | NR | NR | >0.10 | . | | . | | . |  |

“.” signifies the value was not reported.

^a^ Regression coefficients reported from last row of Table III in Crump et al.(Crump et al., 1998), which corresponded to the “extended” adjustment that included social class and parental education variables and omitted the mother-child pair with 86 µg/g MH THg - the single highest value >4x the next highest MH THg and considered an outlier by Crump et al.(Crump et al., 1998) and in the NAS report (2000).

^b^ The regression coefficients used in the meta-analysis (Table 3 of Axelrad et al. (2007)) were taken from the second row of Table III in Crump et al.(1998), which were based on the same adjustment variables originally included by Kjellstrom et al.(1989), but with the highest exposed mother-child pair omitted. These regression coefficients were then rescaled in Axelrad et al. (2007) as partially standardized regression coefficients expressed as β_∆IQ_ (change in IQ score for an increase of 1 µg/g MH THg).

^c^ BMD estimates originally reported in Crump et al. (1998) using the K-power model (with k=1), P(0)=5% and a BMR=10% added-risk, but re-analyzed for BMR=5% extra-risk as reported in Table 7-2 of NAS report (NAS, 2000), which is equivalent to a continuous outcome BMR= -0.36SD by the “hybrid approach” (see Supplemental File S3.A and S3.B).

^d^ Reported in Crump et al. (1998) as 26 tests, but 5 of these “tests” were re-expressions of a single test score, 2 re-expressions for the Burt word recognition test (i.e. score, age-equivalent) and 3 re-expressions for Peabody (i.e. standard score, percentile rank, stanine). When only 1 test score each reported for Burt and Peabody the total outcome tests is 23.

^e^ β>0 reflects improved performance for all outcome tests.

^f^ Regression coefficients were not reported for 18 of the 23 tests, exact p-values were reported for 6 of these 18 and only p>0.1 for the remaining 12 (p.707 of Crump et al. 1998)

# Faroe Islands Cohort 1

There are multiple birth-cohorts from the Faroe Islands exploring the association between neurodevelopment and prenatal methyl mercury (MeHg) exposure, including Cohorts 1, 2, and 3 (Weihe & Joensen, 2012). Cohort 1 recruited pregnant women who gave birth at the national hospital in Torshavn or 2 other more remote hospitals between 1986-1987 (n=997), while Cohort 2 (1994-1995, n=182) and Cohort 3 (1998-2000, n=565) only recruited women who gave birth in Torshavn hospital. The information below describes Faroe Islands Cohort 1, which was the cohort used by several risk assessment organizations to select PoDs.

The Faroe Cohort 1 was a prospective birth cohort in the Faroe Islands, which followed an initial small pilot study that found elevated levels of mercury in cord blood of Faroese mothers. The Faroe Cohort 1 included all eligible singleton mother-infant pairs born between March 1, 1986 to December 31, 1987 at one of three hospitals in the Faroe Islands:

- Hospital 1: Faroe National Hospital in Torshavn (in the largest and capital city on the central island of Streymoy)
- Hospital 2*: Klaksvik Hospital (Klaksvik is Faroe Islands’ second largest city on the northern island of Bordoy)
- Hospital 3*: Suduroy Hosptial (in the village of Tvøroyri on the southern most of the Faroe Islands)

* The identity of Hospital 2 and 3 are not explicitly stated in the published papers. However, the identity of each recruitment hospital were inferred by cross-referencing different publications and comparing the number of participants recruited and mercury biomarkers levels for each of the 3 recruitment hospitals (Grandjean et al., 1992, 1995, 1997; Grandjean & Weihe, 1993).

Covariate data were collected in a questionnaire that was administered by midwives and included lifestyle, dietary, and demographic factors. THg was analyzed in both cord blood (CB) (n=997) and MH (n=1020) collected at the time of birth (Grandjean et al., 1992). The GM of THg in CB was 22.5 µg/L (min=0.9, p25= 13.1, p75= 40.8, max= 351.0 µg/L) and the GM of THg in MH was 4.21 µg/g (min=0.17, p25= 2.53, p75= 7.66, max= 39.1 µg/g) (Debes et al., 2006). Grandjean et al. first reported an assessment of test outcomes at average age of 6.8 yrs as linear regression coefficients expressed as a change in test score for an increase in log_10_-transformed CB THg (Grandjean et al., 1997). These same outcomes were later reanalyzed by the authors as regression coefficients expressed as a change in the test score for an increase in 1 µg/g MH THg, as reported in Budtz-Jorgensen et al. (2005) and included in the NAS report (2000). BMD modelling was also performed by these authors (Budtz-Jørgensen et al., 1999, 2000) and included in the NAS report (2000). A total of 6 of 17 eligible coefficients reported in Budtz-Jorgensen et al. (2005) were included in the meta-analysis by Axelrad et al. (2007) (Table S2.2).

**Table S2.2** Regression coefficients and benchmark dose estimates for **17 tests** from the Faroe Islands Cohort 1 at 6.8 years

| **Avg. child age**  **(years)** | **Regression Coefficient (β = Δ test score an 🠙 of 1 µg/g MH THg) ^a^** | | | | | | **Regression coefficient re-scaled in Axelrad et al. (2007) meta-analysis as β_∆IQ_ (SE) ^b^** | **BMD estimates ^c^ (µg/g MH THg)** | |
| --- | --- | --- | --- | --- | --- | --- | --- | --- | --- |
|  | **Outcome Tests (n=17 ^d^)** | **β ^e^** | **95% LCL** | **95% UCL** | **SE** | **p** |  | **BMD_05_** | **BMDL_05_** |
| 6.8 years | BNT - cues | -0.1065 | . | . | 0.0305 | 0.0005 | . | 15 | 10 |
|  | BNT - no cues | -0.095 | . | . | 0.0315 | 0.003 | -0.260 (0.086) | . | . |
|  | BVMGT - copying errors | 0.0365 ^e^ | . | . | 0.0295 | 0.220 | -0.104 (0.083) | 28 | 15 |
|  | BVMGT - reproduction | 0.0006 | . | . | 0.01 | 0.950 | . | . | . |
|  | CVLT - learning | -0.044 | . | . | 0.052 | 0.400 | . | . | . |
|  | CVLT - long-delay recall | -0.032 | . | . | 0.018 | 0.075 | . | 27 | 14 |
|  | CVLT - recognition | -0.011 | . | . | 0.011 | 0.300 | . | . | . |
|  | CVLT - short-delay recall | -0.029 | . | . | 0.016 | 0.069 | -0.169 (0.093) | . | . |
|  | Finger tap - both hands | -0.0945 | . | . | 0.069 | 0.170 | . | . | . |
|  | Finger tap – preferred hand | -0.0965 | . | . | 0.0365 | 0.008 | . | 20 | 12 |
|  | Finger tap - non-preferred hand | -0.041 | . | . | 0.034 | 0.230 | . | . | . |
|  | NES2 CPT - reaction time | 2.20 ^e^ | . | . | 0.650 | 0.0008 | . | 18 | 10 |
|  | NES2 CPT - total missed | 0.0765 ^e^ | . | . | 0.0565 | 0.180 | . | . | . |
|  | NES2 - hand-eye coordination errors | 0.0028 ^e^ | . | . | 0.0017 | 0.099 | . | . | . |
|  | WISC-R - block design | -0.0875 | . | . | 0.049 | 0.075 | -0.124 (0.057) ^f^ | . | . |
|  | WISC-R - digit span | -0.0125 | . | . | 0.009 | 0.160 |  | . | . |
|  | WISC-R - similarities | -0.0195 | . | . | 0.025 | 0.430 |  | . | . |

“.” signifies the value was not reported.

^a^ Regression coefficients in Table 2 of Budtz-Jorgensen et al. (2005) were reported as β = Δ test score for an 🠙of 10 µg/L CB THg, but re-expressed here as Δ test score for an 🠙of 1 µg/g MH THg to be consistent with the same exposure increase in Tables S2.1, S2.3, and S2.4. Conversions were as follows: reported β ÷ 10 to obtain Δ test score for an 🠙of 1 µg/L CB THg, and then ÷ 0.2 to obtain Δ test score for an 🠙of 1 µg/g MH THg assuming a conversion ratio for MH (µg/g):CB(µg/L) of 0.200:1 as was used by Axelrad et al. (2007).

^b^ The regression coefficients used in the meta-analysis are shown in Table 3 of Axelrad et al. (Axelrad et al., 2007). These values were taken from Table 2 of Budtz-Jorgensen et al. (2005) and rescaled in Axelrad et al. (2007) as partially standardized regression coefficients expressed as β_∆IQ_ (change in IQ score for an increase of 1 µg/g MH THg).

^c^ BMD estimates originally reported using the K-power model (with k=1), P(0)=5% and a BMR=5% extra-risk in Table 1 of Budtz-Jorgensen et al. (1999) and also reported in Table 7-2 and 7-4 of the NAS report (2000), which is equivalent to a continuous outcome BMR= -0.36SD by the “hybrid approach” (see Supplemental File S3.A and S3.B). Note: the NAS reported a BMD_05_ (BMDL_05_) of 17(10) for NES2 CPT reaction time scores, but this was reported as 18(10) in Table 1 of Budtz-Jorgensen et al. (1999). The data from Budtz-Jorgensen et al. (1999) is reported in this document.

^d^ Reported in Table 2 of Budtz-Jorgensen et al. (2005) as 21 separate rows of coefficients, but 7 of these coefficients were re-expressions of the same test score: 3 re-expressions for the WISC similarities (i.e. all children, examiner A, examiner B), WISC block designs (i.e. untransformed, square-root transformed), and NES2 total missed (i.e. untransformed, ln-transformed). When only test 1 score each is reported for WISC similarities, WISC block design, and NES2 total missed then the total number of outcome tests is 17.

^e^ Where indicated, β>0 reflects worse performance on these outcomes tests. For all other tests, β>0 reflects improved performance.

^f^ Axelrad et al. (2007) derived a composite WISC-R full-scale IQ score based on three WISC-R subtests (block design, similarities, digit span).

# Seychelles Main Cohort

Multiple birth-cohorts from the Seychelles Islands were under the umbrella of the Seychelles Child Development Study (SCDS) that explored the association between neurodevelopment and prenatal MeHg exposure. The SCDS included the Pilot study, Main Cohort, and Nutrition Cohorts 1 and 2 (van Wijngaarden et al., 2012). All four SCDS cohorts recruited pregnant women who gave birth on the principal island of Mahe and measured THg in MH: the Pilot study (1985-1986, n=789), Main Cohort (1989-1990, n=740), Nutrition Cohort 1 (2001, n=276), and Nutrition Cohort 2 (2008-2001, n=1265). The latter two nutrition cohorts also collected information on dietary habits (fish intake) and measured biomarkers of fish nutrients (e.g. omega-3 fatty acids). The information below describes the Seychelles Main Cohort, which was the principal Seychelles cohort data used for PoD selection by the US EPA, JECFA, and ATSDR.

The Seychelles Main Cohort was established based on a target population of all Seychellois children born between Feb.28, 1989 to March 1, 1990 and whose mothers were regular residents of the principal island of Mahe, Republic of Seychelles. Maternal hair samples were routinely collected for THg analysis at time of delivery from 99% of all consenting Seychellois mothers during this period. Eligible mother-child pairs registering live births between the target dates were identified retrospectively from a review of obstetric log books and were subsequently invited to participate in the study by health nurses. Of the 779 mother-child pairs that were initially enrolled (50% of all Seychellois births during the study period), 740 pairs were included in the study (excluded were 15 with insufficient hair for THg analysis, 6 twins, and 18 who met a priori exclusion criteria) (Marsh et al., 1995; Myers et al., 1995; Shamlaye et al., 1995). The average THg was reported as a p50=5.84 µg/g MH (min=0.43, p25= 3.19, p75= 8.92, max= 26.85 µg/g) (Davidson et al., 1998). Neurological exams and maternal questionnaires were completed at the first follow-up at 6.5 months, with multiple separate follow-up assessments up to the most recent at 24 yrs age. Multivariate linear regression of test outcomes at 5.5 yrs (66 months) from Davidson et al. (1998) and at 8.9 yrs (107 months) from Myers et al. (2003) reported coefficients as a change in test scores for an increase in 1 µg/g MH THg. Outcome test scores at 5.5 yrs were also analyzed by Crump et al. (2000) using BMD modelling of participant-level data with “extended covariates”, a P(0)=5% background abnormal and a continuous outcome BMR= -0.61SD based on the “hybrid approach”, which was equivalent to a binary outcome BMR=10% added-risk. At the request of the NAS committee, the BMD results were reanalyzed by Crump using a continuous outcome BMR= -0.36SD, which is equivalent to a binary outcome BMR=5% added-risk by the “hybrid approach” (see Supplemental File S3.A and S3.B). Results of the BMD analysis were reported in Table 7-2 of the NAS report (2000) (Table S2.3). Regression coefficients for outcomes assessed in this cohort at 8.9 years were reported in Myers et al. (2003), with 5 of 22 eligible coefficients selected for inclusion in the meta-analysis by Axelrad et al. (2007) (Table S2.4).

**Table S2.3** Regression coefficients and benchmark dose estimates for **6 tests** from the Seychelles Main Cohort at 5.5 years

| **Avg. child age (years)** | **Regression Coefficient (β = Δ test for an 🠙 of 1 µg/g MH THg) ^a^** | | | | | | **Regression coefficient re-scaled in Axelrad et al. (2007) meta-analysis as β_∆IQ_ (SE)** | **BMD estimates ^b^ (µg/g MH THg)** | |
| --- | --- | --- | --- | --- | --- | --- | --- | --- | --- |
|  | **Outcome Tests (n=6)** | **β** | **95% LCL** | **95% UCL** | **SE** | **p** |  | **BMD_05_** | **BMDL_05_** |
| 5.5 years  (66 months) | BVMGT - copying errors | 0.04^d^ | . | . | 0.05 | . | . | *** ^c^ | 25 |
|  | Child Behavior Checklist | -0.11 | . | . | 0.09 | . | . | 21 | 17 |
|  | MSCA - general cognitive | -0.057 | . | . | 0.10 | . | . | *** ^c^ | 23 |
|  | Preschool Language Scale | 0.13 | . | . | 0.057 | . | . | *** ^c^ | 23 |
|  | WJTA - applied problems | 0.11 | . | . | 0.14 | . | . | *** ^c^ | 22 |
|  | WJTA - letter/word recognition | 0.02 | . | . | 0.09 | . | . | *** ^c^ | 22 |

“.” signifies the value was not reported.

^a^ Regression coefficients reported from Table 3 of Davidson et al. (1998).

^b^ BMD estimates originally reported in Table 2 of Crump et al. (2000) using the K-power model (with k=1), background probability of an abnormal score of P(0)=5%, and a binary outcome BMR=10% added-risk, equivalent to a continuous outcome BMR= -0.61SD by the “hybrid approach”, but re-analyzed and reported in Table 7-2 of the NAS report (2000) for a binary outcome BMR=5% added-risk which is equivalent to a continuous outcome BMR= -0.36SD by the “hybrid approach” (see Supplemental File S3.A and S3.B)

^c^ ***: denotes BMD_05_ values were undefined since the central estimate was in the direction of benefit (Crump et al., 2000; NAS, 2000).

^d^ Where indicated, β>0 reflects worse performance on these outcomes tests. For all other tests, β>0 reflects improved performance.

**Table S2.4** Regression coefficients and benchmark dose estimates for **22 tests** from the Seychelles Main Cohort at 8.9 years

| **Avg. child age**  **(years)** | **Regression Coefficient (β = Δ test score for an 🠙 of 1 µg/g MH THg) ^a^** | | | | | | **Regression coefficient re-scaled in Axelrad et al. (2007) meta-analysis as β_∆IQ_ (SE) ^c^** | **BMD estimates (µg/g MH THg)** | |
| --- | --- | --- | --- | --- | --- | --- | --- | --- | --- |
|  | **Outcome Tests (n=22)** | **β ^b^** | **95% LCL** | **95% UCL** | **SE** | **p** |  | **BMD_05_** | **BMDL_05_** |
| 8.9 years  (107 months) | BNT - total score | -0.012 | . | . | 0.046 | 0.79 | -0.038 (0.144) | . | . |
|  | Bruininks-Oseretsky Test - motor proficiency | 0.093 | . | . | 0.056 | 0.10 | . | . | . |
|  | Child Behavior Check List | -0.031^c^ | . | . | 0.10 | 0.76 | . | . | . |
|  | CPT-Connor's - attentiveness | -0.0063 | . | . | 0.10 | 0.95 | . | . | . |
|  | CPT-Connor's - reaction time | -0.13^c^ | . | . | 0.16 | 0.41 | . | . | . |
|  | CPT-Connor's – risk taking | 0.11 | . | . | 0.22 | 0.60 | . | . | . |
|  | Connors Teacher Rating Scale - hyperactivity index ^d^ | -0.0067 | . | . | 0.0023 | 0.004 | . | . | . |
|  | CVLT - long-delay recall | 0.011 | . | . | 0.010 | 0.28 | . | . | . |
|  | CVLT - short-delay recall | 0.013 | . | . | 0.010 | 0.19 | 0.19 (0.144) | . | . |
|  | Finger tap – preferred hand | -0.050 | . | . | 0.053 | 0.34 | . | . | . |
|  | Finger tap - non-preferred hand | 0.016 | . | . | 0.041 | 0.69 | . | . | . |
|  | Grooved pegboard – preferred hand ^e^ | 3.3*10^-5 b^ | . | . | 1.9*10^-5^ | 0.08 | . | . | . |
|  | Grooved pegboard – non-preferred (male) ^e^ | 6.5*10^-5 b^ | . | . | 2.5*10^-5^ | 0.01 | . | . | . |
|  | Grooved pegboard – non-preferred (female) ^e^ | -2.5*10^-5 b^ | . | . | 2.6*10^-5^ | 0.34 | . | . | . |
|  | Haptic discrimination test | -0.010 | . | . | 0.018 | 0.60 | . | . | . |
|  | Trail Making Test - time A | 0.004^c^ | . | . | 0.004 | 0.33 | . | . | . |
|  | Trail Making Test - time B | 0.007^c^ | . | . | 0.005 | 0.17 | . | . | . |
|  | Visual Motor Integration – Beery-Buktenica | -0.010 | . | . | 0.12 | 0.93 | -0.013 (0.150) | . | . |
|  | WISC-III - FIQ | -0.13 | . | . | 0.10 | 0.20 | -0.17 (0.130) | . | . |
|  | WJTA - applied problems | -0.057 | . | . | 0.15 | 0.71 | . | . | . |
|  | WJTA - letter-word recognition | 0.19 | . | . | 0.39 | 0.62 | . | . | . |
|  | WRAML - design memory | -0.021 | . | . | 0.029 | 0.48 | -0.109 (0.150) | . | . |

“.” signifies the value was not reported.

^a^ Regression coefficients reported from Tables 2, 3, and 4 of Myers et al. (2003).

^b^ Where indicated, β>0 reflects worse performance on these outcome tests. For all other tests, β>0 reflects improved performance.

^c^ The regression coefficients used in the meta-analysis from Table 3 of Axelrad et al. (2007) were taken from the second last columns of Tables 2, 3, and 4 of Myers et al. (2003). These regression coefficients were then rescaled in Axelrad et al. (2007) as partially standardized regression coefficients expressed as β_∆IQ_ (change in IQ score for an increase of 1 µg/g MH THg).

^d^ Modelled as transformed score (transformation not reported).

^e^ Modelled as transformed score (negative reciprocal of the test score).

# References

Axelrad, D. A., Bellinger, D. C., Ryan, L. M., & Woodruff, T. J. (2007). Dose-response relationship of prenatal mercury exposure and IQ: an integrative analysis of epidemiologic data. *Environmental Health Perspectives*, *115*(4), 609–615. https://ehp.niehs.nih.gov/doi/10.1289/ehp.9303

Budtz-Jørgensen, E., Debes, F., Grandjean, P., & Weihe, P. (2005). *Adverse mercury effects in 7 year old children expressed as loss in “IQ.” Report to the U.S. Environmental Protection Agency. Document ID EPA-HQ-OAR-2002-0056-6046.* https://www.regulations.gov/document/EPA-HQ-OAR-2002-0056-6046

Budtz-Jørgensen, E., Grandjean, P., Keiding, N., White, R. F., & Weihe, P. (2000). Benchmark dose calculations of methylmercury-associated neurobehavioural deficits. *Toxicology Letters*, *112*–*113*, 193–199. https://doi.org/10.1016/s0378-4274(99)00283-0

Budtz-Jørgensen, E., Keiding, N., & Grandjean, P. (1999). *Benchmark Modeling of the Faroese Methylmercury Data. Final Report to the US EPA. Odense and Copenhagen, Denmark, 6 July, 1999. Research Report 99/5. Department of Biostatistics, University of Copenhagen.* https://hero.epa.gov/hero/index.cfm/reference/details/reference_id/3841176

Crump, K. S., Kjellström, T., Shipp, A. M., Silvers, A., & Stewart, A. (1998). Influence of prenatal mercury exposure upon scholastic and psychological test performance: benchmark analysis of a New Zealand cohort. *Risk Analysis : An Official Publication of the Society for Risk Analysis*, *18*(6), 701–713. https://doi.org/10.1023/b:rian.0000005917.52151.e6

Crump, K. S., Van Landingham, C., Shamlaye, C., Cox, C., Davidson, P. W., Myers, G. J., & Clarkson, T. W. (2000). Benchmark concentrations for methylmercury obtained from the Seychelles Child Development Study. *Environmental Health Perspectives*, *108*(3), 257–263. https://doi.org/10.1289/ehp.00108257

Davidson, P. W., Myers, G., Cox, C., Axtell, C., Shamlaye, C., Sloane-Reeves, J., Cernichiari, E., Needham, L., Choi, A., Wang, Y., Berlin, M., & Clarkson, T. W. (1998). Effects of Prenatal and Postnatal Methylmercury Exposure From Fish Consumption on NeurodevelopmentOutcomes at 66 Months of Age in the Seychelles Child Development Study. *JAMA*, *280*(8), 701–707. https://doi.org/10.1001/jama.280.8.701

Debes, F., Budtz-Jørgensen, E., Weihe, P., White, R., & Grandjean, P. (2006). Impact of prenatal methylmercury exposure on neurobehavioral function at age 14 years. *Neurotoxicology and Teratology*, *28*(5), 536–547. https://www.sciencedirect.com/science/article/pii/S089203620600050X?via%3Dihub

Grandjean, P., & Weihe, P. (1993). Neurobehavioral effects of intrauterine mercury exposure: potential sources of bias. *Environmental Research*, *61*(1), 176–183. https://doi.org/10.1006/enrs.1993.1062

Grandjean, P., Weihe, P., Jørgensen, P. J., Clarkson, T., Cernichiari, E., & Viderø, T. (1992). Impact of Maternal Seafood Diet on Fetal Exposure to Mercury, Selenium, and Lead, Archives of Environmental Health. *An International Journal*, *47*(3), 185–195. https://doi.org/10.1080/00039896.1992.9938348

Grandjean, P., Weihe, P., & White, R. F. (1995). Milestone development in infants exposed to methylmercury from human milk. *Neurotoxicology*, *16*(1), 27–34. https://pubmed.ncbi.nlm.nih.gov/7603642/

Grandjean, P., Weihe, P., White, R. F., Debes, F., Araki, S., Yokoyama, K., Murata, K., Sørensen, N., Dahl, R., & Jørgensen, P. J. (1997). Cognitive Deficit in 7-Year-Old Children with Prenatal Exposure to Methylmercury. *Neurotoxicology and Teratology*, *19*(6), 417–428. https://doi.org/10.1016/S0892-0362(97)00097-4

Kjellström, T., Kennedy, P., Wallis, S., & Mantell, C. (1986). *Physical and mental development of children with prenatal exposure to mercury from fish: Stage 1: Preliminary tests at age 4*. National Swedish Environmental Protection Board. https://hawcproject.org/study/attachment/4150/

Kjellström, T., Kennedy, P., Wallis, S., Stewart, A., Friberg, L., Lind, B., Wutherspoon, T., & Mantell, C. (1989). *Physical and mental development of children with prenatal exposure to mercury from fish. Stage 2: Interviews and psychological tests at age 6*. National Swedish Environmental Protection Board. https://hawcproject.org/media/study-attachment/Kjellström_1989_-_New_Zealand.pdf

Marsh, D. O., Clarkson, T. W., Myers, G. J., Davidson, P. W., Cox, C., Cernichiari, E., Tanner, M. A., Lednar, W., Shamlaye, C., Choisy, O., Hoareau, C., & Berlin, M. (1995). The Seychelles study of fetal methylmercury exposure and child development: Introduction. *NeuroToxicology*, *16*(4), 583–596. https://pubmed.ncbi.nlm.nih.gov/8714865/

Myers, G. J., Davidson, P. W., Cox, C., Shamlaye, C. F., Palumbo, D., Cernichiari, E., Sloane-Reeves, J., Wilding, G. E., Kost, J., Huang, L.-S., & Clarkson, T. W. (2003). Prenatal methylmercury exposure from ocean fish consumption in the Seychelles child development study. *Lancet (London, England)*, *361*(9370), 1686–1692. https://doi.org/10.1016/S0140-6736(03)13371-5

Myers, G. J., Marsh, D. O., Davidson, P. W., Cox, C., Shamlaye, C. F., Tanner, M., Choi, A., Cernichiari, E., Choisy, O., & Clarkson, T. W. (1995). Main neurodevelopmental study of Seychellois children following in utero exposure to methylmercury from a maternal fish diet: Outcome at six months. *NeuroToxicology*, *16*(4), 653–664. https://pubmed.ncbi.nlm.nih.gov/8714870/

NAS. (2000). Toxicological Effects of Methylmercury. In *Toxicological Effects of Methylmercury (National Academy of Sciences)*. National Academies Press. https://doi.org/10.17226/9899

Shamlaye, C. F., Marsh, D. O., Myers, G. J., Cox, C., Davidson, P. W., Choisy, O., Cernichiari, E., Choi, A., Tanner, M. A., & Clarkson, T. W. (1995). The Seychelles child development study on neurodevelopmental outcomes in children following in utero exposure to methylmercury from a maternal fish diet: Background and demographics. *NeuroToxicology*, *16*(4), 597–612. https://pubmed.ncbi.nlm.nih.gov/8714866/

van Wijngaarden, E., Myers, G. J., Shamlaye, C. F., Strain, J. J., & Davidson, P. W. (2012). The Impact of Prenatal Exposure to Methylmercury and Maternal Nutritional Status on Child Development: Findings from the Seychelles Child Development Study. In *Methylmercury and Neurotoxicity* (pp. 37–53). Springer US. https://doi.org/10.1007/978-1-4614-2383-6_3

Weihe, P., & Joensen, H. D. (2012). Dietary recommendations regarding pilot whale meat and blubber in the Faroe Islands. *International Journal of Circumpolar Health*, *71*, 18594. https://doi.org/10.3402/ijch.v71i0.18594
