# Supplementary material for: Neurodevelopmental effects of methylmercury (MeHg): a review of epidemiological points of departure (PoDs), toxicological reference values (TRVs), and key uncertainties in human health risk assessment
Source: Arch Toxicol. 2026 Mar 10;100(6):2191–219. doi: 10.1007/s00204-026-04345-8 (PMC13221417; doi:10.1007/s00204-026-04345-8)
Supplement: Supplementary file 3 — Supplementary file3 (DOCX 136 kb) [file 204_2026_4345_MOESM3_ESM.docx]

**Supplemental File S3A.**

**Benchmark Response (BMR) Conversions between Continuous and Binary Outcomes using the “Hybrid Approach”**

*Archives of Toxicology*

Neurodevelopmental effects of methylmercury (MeHg): A review of epidemiological points of departure (PoDs), toxicological reference values (TRVs), and key uncertainties in human health risk assessment

Blechinger, Scott R.^1^* (ORCID 0000-0002-4991-4597, Scopus ID 6506155596)

Singh, Kavita^2^ (ORCID N/A, Scopus ID 58382466900)

Afghan, Abdul^1^ (ORCID N/A, Scopus ID 58522410100)

Smith, Catherine A.^1^ (ORCID N/A, Scopus ID 46461849300)

^1^ Bureau of Chemical Safety, Food and Nutrition Directorate, Health Canada, Ottawa, Canada

^2^ Environmental Health Science and Research Bureau, Health Canada, Ottawa, Canada

*Corresponding author: scott.blechinger@hc-sc.gc.ca

In chemical risk assessment, the benchmark dose (BMD) is an exposure level that corresponds to a toxicological relevant change in an outcome called the benchmark response (BMR) (EFSA, 2022; Haber et al., 2018). For binary or dichotomous outcomes (e.g. presence vs absence of abnormal test score, histopathological finding, etc.), the BMR represents a fixed increased probability of an “abnormal” outcome and is defined typically as a percent added-risk or extra-risk^[[1]](#footnote-1)^ (US EPA, 2025b). In contrast, for continuous outcomes (e.g. IQ score) the BMR represents a fixed change in a continuous outcome and is defined as either a change in relative deviation or standard deviation^[[2]](#footnote-2)^ of the outcome variable (US EPA, 2025a). An additional variant to selecting the BMR for a continuous outcome is to use the “hybrid approach” which selects a standard deviation change BMR that is mathematically equivalent to a binary BMR defined as either an added-risk or extra-risk (US EPA, 2025a). Multiple publications discuss the theoretical basis and methods involved in the “hybrid approach” (Budtz-Jørgensen et al., 2001; Coull et al., 2003; Crump, 1995, 2002; Crump et al., 1998, 2000; NAS, 2000; Sand et al., 2008; Suwazono et al., 2011).

Using these “hybrid approach” methods, Crump et al. (1995; 2000) presented tables of binary outcome BMRs defined as a % added-risk^[[3]](#footnote-3)^ of an “abnormal” outcome (e.g. low IQ score) for a given background “abnormal” probability P(0) and the mathematically corresponding continuous outcome BMRs for a standard deviation change in the outcome (ΔSDy). For example, Crump et al. reported that a binary outcome BMR of 5% added-risk for either a 10% or 5% background abnormal probability P(0) mathematically corresponds to a continuous outcome BMR in units of standard deviation change of the outcome variable of 0.61SDy and 0.36SDy, respectively (Crump, 1995; Crump et al., 1998, 2000). The continuous outcome BMR as a standard deviation change (ΔSDy) can be re-expressed in the original units of the outcome by multiplying by the standard deviation of the outcome: BMR_Δy_ = BMR_ΔSDy_ *SDy (e.g. for IQ test scores with a mean=100 and SD=15 where a lower score indicates worse function, a BMR_ΔSDy_ of -0.36SDy corresponds to BMR_Δy_ of -5.4 IQ points (i.e. BMR_Δy_ = -0.36SDy * 15 = -5.4).

The continuous outcome BMR_ΔSDy_ of -0.36SDy was subsequently used in the BMD modelling estimates for prenatal MeHg exposure reported by the NAS (2000) and US EPA (2001b) for data from the Faroe Islands Cohort 1 (Budtz-Jørgensen et al., 1999, 2000), New Zealand Cohort (Crump et al., 1998), and Seychelles Main Cohort (Crump et al., 2000). While these BMD estimates have been reported as BMD_05_ and BMDL_05_ values (EFSA, 2012; US EPA, 2001a) for a binary outcome BMR_AR_ of 5% added risk^[[4]](#footnote-4),^^[[5]](#footnote-5)^ with a background abnormal probability P(0) of 5%, these BMD estimates were actually based on the “hybrid approach” since the outcome test scores were modelled as continuous outcome variables, which corresponded to a standard deviation change in a continuous outcome BMR_ΔSDy_ of -0.36SDy, which is equivalent to a BMR_Δy_ of -5.4 IQ points. These continuous outcome BMRs were not explicitly stated in the assessments by the NAS (2000) and US EPA (2001b) but their equivalence to binary outcome BMRs via the “hybrid approach” are worth noting for clarity:

binary outcome BMR_AR_ of 5% added risk with a background abnormal probability P(0) of 5%

= continuous outcome BMR_ΔSDy_ of -0.36SDy

= continuous outcome BMR_Δy_ of -5.4 IQ points

There is uncertainty regarding the most appropriate toxicologically relevant continuous outcome BMR to select for neurodevelopmental outcomes such as IQ test scores. While the NAS (2000) and US EPA (2001b) reported BMD modelling estimates based on a continuous outcome BMR_Δy_ of -5.4 IQ points (= BMR_ΔSDy_ of -0.36SDy), a lower BMR_Δy_ of -1 IQ point (= BMR_ΔSDy_ of 0.067SDy) has been used for risk assessments of lead (Budtz-Jørgensen et al., 2013; EFSA, 2010) and fluoride (Grandjean et al., 2022). The EFSA also reported that a continuous outcome BMR_Δy_ of -1 IQ point corresponded to a “BMR of 1%” with the BMD estimates reported as “BMD_01_” and “BMDL_01_” (EFSA, 2010). However potential confusion may arise since this 1% was for a “relative deviation” continuous outcome BMR definition (e.g. 1 IQ point change ÷ mean of 100 IQ points *100 = 1% relative deviation BMR) (EFSA, 2010) and not a binary outcome BMR of % added-risk using the “hybrid approach”. As such, the “BMR of 1%” for a “relative deviation” continuous outcome BMR for lead used by EFSA (2010) cannot be directly compared to the binary outcome BMR of 5% added-risk (with P(0)=5% abnormal background) using the “hybrid approach” for mercury (NAS, 2000; US EPA, 2001b). However, they can be compared when expressed as continuous outcome BMRs: as BMR_ΔSDy_ (-0.067SDy for lead vs -0.36SDy for mercury) or as BMR_Δy_ (-1 IQ point for lead vs -5.4 IQ points for mercury). To express the continuous BMR_Δy_ of -1 IQ point for lead (=BMR_ΔSDy_ of -0.067SDy) as a binary outcome BMR % added-risk using the “hybrid approach” as done for mercury, the same the background probability of an “abnormal” score P(0)=5% would need to be applied and the BMR as % added-risk calculated afterward.

There has been a lack of consensus or guidance on the appropriate choice of BMR to use for BMD modelling of continuous neurodevelopmental outcomes, such as IQ scores, as well as inconsistent reporting of the BMR definition and methods used. Therefore, the “hybrid approach” methods from Crump et al. have been used in Supplemental File S3A to illustrate conversions between binary outcome BMRs (for both added-risk and extra-risk) and the mathematically equivalent corresponding continuous outcome BMR as a standard deviation change (BMR_ΔSDy_) or change in the outcome units (BMR_Δy_). Table S3A.1 shows simplifications of previously published formulas and methodology for the “hybrid approach” (Budtz-Jørgensen et al., 2001; Coull et al., 2003; Crump, 1995, 2002; Crump et al., 1998, 2000; NAS, 2000; Sand et al., 2008; Suwazono et al., 2011) and example calculations are provided in Table S3A.2 for a variety of typical scenarios. Finally, Table S3A.3 shows a summary of different combinations of binary outcome BMRs (as % added-risk and % extra-risk) and their mathematically equivalent corresponding continuous outcome BMRs (as BMR_ΔSDy_ and BMR_Δy_) expanding on the abbreviated conversion tables previously reported by Crump et al. (1995; 1998, 2000). A more extensive table of BMR conversions using the same “hybrid approach” methods is provided as MS Excel worksheets in Supplemental File S3B and allows user-defined inputs to calculate custom BMRs.

**Table S3A.1 BMR “hybrid approach” definitions and equations** (Budtz-Jørgensen et al., 2001; Coull et al., 2003; Crump, 1995, 2002; Crump et al., 1998, 2000; NAS, 2000; Sand et al., 2008; Suwazono et al., 2011)

| **Parameter** | **Definition** | **Equations** |
| --- | --- | --- |
| **P(0)** | The P(0) is the background probability of an “abnormal” outcome when exposure = 0.  (Note: for observational epidemiology studies where there is theoretically always some low level of exposure, P(0) can be considered also to apply to the general “unexposed” population, i.e. without known occupational or point-source environmental exposures). | **binary outcome P(0) calculation:**  N/A (no formula, simply specified as some default background % such as 1%, 5%, 10%, etc. corresponding to percentiles in left or right tail of a normal distribution)  **continuous outcome P(0) calculation:**  $\boldsymbol{P}\left( \boldsymbol{0} \right)\boldsymbol{=f}\left[ \frac{\boldsymbol{C-}\boldsymbol{\mu}_{\boldsymbol{(0)}}}{\boldsymbol{SDy}} \right]\boldsymbol{= f}\left[ \boldsymbol{z}_{\boldsymbol{@P(0)}} \right]$ [1]  where:  ***C*** = The value of the outcome above/below which defines “abnormal” (e.g. for IQ scores, intellectual disability is sometimes defined as an IQ score ≤ 70 ± 5 measurement error, so an IQ score ≤ 65 to ≤75 maybe considered “abnormal” ^[[6]](#footnote-6)^). When a binary outcome P(0) is pre-specified (e.g. 5%) and the mean µ_(0)_ and SDy of the outcome are known, then the corresponding cut-off value *C* can be solved for using equation [1]  $\boldsymbol{\mu}_{\boldsymbol{(}\boldsymbol{0}\boldsymbol{)}}$**=** The mean of the outcome when exposure = 0 (or in the general “unexposed” population) (e.g. for IQ scores a population mean = 100)  ***SDy*** = The standard deviation of the outcome in the “unexposed” population (e.g. for IQ scores, SD=15)  $\boldsymbol{z}_{\boldsymbol{@}\boldsymbol{P}\boldsymbol{(}\boldsymbol{0}\boldsymbol{)}}$ *=* The z-value at P(0) (i.e. probability of observing a value of *C* given a mean *µ_(0)_* and standard deviation *SDy*)  $\boldsymbol{f}$ = standard normal distribution function which returns the tail probability or area of the standard normal curve for a given z-value, in this case z_@P(0)_ (e.g. calculated with MS Excel ‘NORM.S.DIST(z-value,TRUE)’, Stata ‘display normal(z-value)’, or R ‘pnorm(z-value)’) |
| **binary outcome BMR_%_** | For a binary outcome BMR using the “hybrid approach”, the BMR may be defined as an increased **probability of an “abnormal” outcome** above the background P(0), and is typically calculated as either % *added*-risk (also called additional risk or excess risk) or % *extra*-risk. | **BMR_%_ “hybrid approach” calculations:**  **BMR % added-risk:**  $\boldsymbol{BMR}_{\boldsymbol{\%}\boldsymbol{AR}}\boldsymbol{=}\boldsymbol{P}\left( \boldsymbol{BMD} \right)\boldsymbol{-} \boldsymbol{P}\left( \boldsymbol{0} \right)$ [2]  or  **BMR % extra-risk:**  $\boldsymbol{BMR}_{\boldsymbol{\%}\boldsymbol{ER}}\boldsymbol{=}\frac{\boldsymbol{[}\boldsymbol{P}\left( \boldsymbol{BMD} \right)\boldsymbol{-} \boldsymbol{P}\left( \boldsymbol{0} \right)\boldsymbol{]}}{\boldsymbol{[}\boldsymbol{1}\boldsymbol{-}\boldsymbol{P}\left( \boldsymbol{0} \right)\boldsymbol{]}}$ [3]  where:  ***P(BMD*)** = The probability (%) of an “abnormal” outcome at the exposure = BMD)  ***P(0)*** = defined above (The background probability (%) of an “abnormal” outcome when exposure = 0) |
| **P(BMD)** | The P(BMD) is the probability of an increased “abnormal” outcome when exposure = BMD.  The calculations for a P(BMD) differ slightly for a binary outcome BMR as % *added*-risk (also called additional risk or excess risk) vs % *extra*-risk; this difference becomes insignificant when the background “abnormal” probability P(0) is low. | **binary outcome P(BMD) calculation:**  Calculated by rearrangement from equation [2] for a BMR % added-risk:  ${\boldsymbol{P}\boldsymbol{(}\boldsymbol{BMD}\boldsymbol{)}}_{\boldsymbol{\%}\boldsymbol{AR}}=P\left( 0 \right)+ BMR$ [4]  Calculated by rearrangement from equation [3] for a BMR % extra-risk:  ${\boldsymbol{P}\boldsymbol{(}\boldsymbol{BMD}\boldsymbol{)}}_{\boldsymbol{\%}\boldsymbol{ER}}=P\left( 0 \right)+ BMR*[1-P\left( 0 \right)]$ [5]  **continuous outcome P(BMD) calculation:**  $\boldsymbol{P}\left( \boldsymbol{BMD} \right)\boldsymbol{=f}\left[ \frac{\boldsymbol{C-}\boldsymbol{\mu}_{\boldsymbol{(BMD)}}}{\boldsymbol{SDy}} \right]\boldsymbol{= f}\left[ \boldsymbol{z}_{\boldsymbol{@P(BMD)}} \right]$ [6]  where:  ***C*** = The value of the outcome above/below which defines “abnormal” (e.g. for IQ scores, intellectual disability is sometimes defined as an IQ score ≤ 70 ± 5 measurement error, so an IQ score ≤ 65 to ≤75 maybe considered “abnormal”).  $\boldsymbol{\mu}_{\boldsymbol{(}\boldsymbol{BMD}\boldsymbol{)}}$= mean of the outcome when exposure = BMD; typically this is not known but solved for by rearrangement of [6]  ***SDy*** = standard deviation of the outcome (e.g. for IQ scores, SD=15)  $\boldsymbol{z}_{\boldsymbol{@}\boldsymbol{P}\boldsymbol{(}\boldsymbol{BMD}\boldsymbol{)}}$ *=* z-value at P(BMD), where P(BMD) is typically calculated from either equations [4] or [5] for % added risk or % extra risk respectively  $\boldsymbol{f}$ = standard normal distribution function which returns the tail probability or area of the standard normal curve for a given z-value, in this case z_@P(BMD)_ (e.g. calculated with MS Excel ‘NORM.S.DIST(z-value,TRUE)’, Stata ‘normal(z-value)’, or R ‘pnorm(z-value)’) |
| **continuous outcome BMR_ΔSDy_** | For a continuous outcome (e.g. IQ scores), the BMR is a toxicologically relevant change in the outcome at the benchmark dose (BMD). The BMR_ΔSDy_ expression is a **change in the continuous outcome relative to the standard deviation of the outcome (ΔSDy)**, which can also be expressed as a difference in the z-value for P(0) and P(BMD) | **continuous outcome BMR_ΔSDy_ calculation:**  $\boldsymbol{BMR}_{\boldsymbol{DSDy}}\boldsymbol{=}\left[ \frac{\boldsymbol{\mu}_{\left( \boldsymbol{BMD} \right)}\boldsymbol{-} \boldsymbol{\mu}_{\left( \boldsymbol{0} \right)}}{\boldsymbol{SDy}} \right]$ [7]  where:  ***µ_(BMD)_*** = The mean of the outcome when exposure = BMD (typically not known but solved for  $\boldsymbol{\mu}_{\left( \boldsymbol{0} \right)}$= The mean of the outcome when exposure = 0 (or in the general “unexposed” population) (e.g. for IQ scores a population mean = 100)  ***SDy*** = The standard deviation of the outcome (e.g. for IQ scores, SD=15)    or  $\boldsymbol{BMR}_{\boldsymbol{DSDy}}\boldsymbol{=}\boldsymbol{f}^{\boldsymbol{-}\boldsymbol{1}} \left[ \boldsymbol{P}\left( \boldsymbol{0} \right)\boldsymbol{]-}\boldsymbol{f}^{\boldsymbol{-}\boldsymbol{1}}\boldsymbol{[}\boldsymbol{P}\boldsymbol{(}\boldsymbol{BMD}\boldsymbol{)} \right]\boldsymbol{=}\left[ \boldsymbol{z}_{\boldsymbol{@}\boldsymbol{P}\left( \boldsymbol{0} \right)\boldsymbol{-}}\boldsymbol{z}_{\boldsymbol{@}\boldsymbol{P}\left( \boldsymbol{BMD} \right)} \right]$ [8]    where:  $\boldsymbol{f}^{\boldsymbol{-}\boldsymbol{1}}$ = The inverse standard normal distribution function which returns the z-value corresponding to a given probability, in this case either P(0) or P(BMD) (e.g. each z-value calculated with MS Excel ‘NORM.S.INV(probability)’, Stata ‘invnormal(probability)’, or R ‘qnorm(probability)’)  $\boldsymbol{z}_{\boldsymbol{@}\boldsymbol{P}\boldsymbol{(}\boldsymbol{0}\boldsymbol{)}}$ *=* The z-value at P(0), where P(0) is pre-specified % or calculated using [1]  $\boldsymbol{z}_{\boldsymbol{@}\boldsymbol{P}\boldsymbol{(}\boldsymbol{BMD}\boldsymbol{)}}$ *=* The z-value at P(BMD), where P(BMD) is typically calculated from either equations [4] or [5] for BMR % added risk or BMR % extra risk respectively |
| **continuous outcome BMR_Δy_** | For a continuous outcome (e.g. IQ scores), the BMR is a toxicologically relevant change in the outcome at the benchmark dose (BMD). The BMR_Δy_ expression is an absolute **change in units of the continuous outcome (Δy).** | **continuous outcome BMR_ΔSDy_ calculation:**  $\boldsymbol{BMR}_{\boldsymbol{Dy}}\boldsymbol{=}\left[ \boldsymbol{\mu}_{\left( \boldsymbol{BMD} \right)}\boldsymbol{-} \boldsymbol{\mu}_{\left( \boldsymbol{0} \right)} \right]$ [9]  where:  ***µ_(BMD)_*** = The mean of the outcome when exposure = BMD  $\boldsymbol{\mu}_{\left( \boldsymbol{0} \right)}$= The mean of the outcome when exposure = 0 (or in the general “unexposed” population) (e.g. for IQ scores a population mean = 100)  or  [9] can be also obtained by rearrangement of [7] for BMR_ΔSDy_:  $\boldsymbol{BMR}_{\boldsymbol{Dy}}\boldsymbol{=}\boldsymbol{BMR}_{\boldsymbol{DSDy}}\boldsymbol{*}\boldsymbol{SDy}$ [10]  or  $\boldsymbol{BMR}_{\boldsymbol{Dy}}\boldsymbol{=}\left[ \boldsymbol{f}^{\boldsymbol{-}\boldsymbol{1}}\boldsymbol{P}\left( \boldsymbol{0} \right)\boldsymbol{]-}\boldsymbol{f}^{\boldsymbol{-}\boldsymbol{1}}\boldsymbol{P}\boldsymbol{(}\boldsymbol{BMD}\boldsymbol{)} \right]\boldsymbol{*}\boldsymbol{SDy}\boldsymbol{=}\left[ \boldsymbol{z}_{\boldsymbol{@}\boldsymbol{P}\left( \boldsymbol{0} \right)\boldsymbol{-}}\boldsymbol{z}_{\boldsymbol{@}\boldsymbol{P}\left( \boldsymbol{BMD} \right)} \right]\boldsymbol{*}\boldsymbol{SDy}$ [11]    where:  $\boldsymbol{f}^{\boldsymbol{-}\boldsymbol{1}}$ = The inverse standard normal distribution function which returns the z-value corresponding to a given probability, either P(0) or P(BMD) (e.g. each z-value calculated with MS Excel ‘NORM.S.INV(probability)’, Stata ‘invnormal(probability)’, or R ‘qnorm(probability)’)  $\boldsymbol{z}_{\boldsymbol{@}\boldsymbol{P}\boldsymbol{(}\boldsymbol{0}\boldsymbol{)}}$ *=* The z-value at P(0), where P(0) is pre-specified % or calculated using [1]  $\boldsymbol{z}_{\boldsymbol{@}\boldsymbol{P}\boldsymbol{(}\boldsymbol{BMD}\boldsymbol{)}}$ *=* The z-value at P(BMD), where P(BMD) is typically calculated from either equations [4] or [5] for BMR % added risk or BMR % extra risk respectively  ***SDy*** = The standard deviation of the outcome (e.g. for IQ scores in a general “unexposed” population, SD=15) |

Using the equations in Table S3A.1, example calculations are provided for different scenarios depending on the inputs available:

**Table S3A.2 BMR “hybrid approach” calculations for examples using equations 1-11 defined in Table S3A.1**

| **Example** | **Calculations** |
| --- | --- |
| **Example 1:**  **unknown:**  BMR_ΔSDy_  BMR_Δy_  **provided:**  P(0)  BMR_%AR_  µ_(0)_  SDy | The evaluations by the NAS (2000) and US EPA (US EPA, 2001b) reported BMD estimates for continuous neurodevelopmental test scores, but only reported a binary outcome BMR of 5% added-risk and a background probably of an “abnormal” outcome of 5% when exposure = 0.  The “hybrid approach” gives the equivalent continuous outcome BMR_ΔSDy_:  Step 1:  Specify the known parameters provided:  P(0) = 0.05 (i.e. 5%)  BMR_%AR_ = 0.05 (i.e. 5%)  Step 2:  Using [4] the P(BMD) % added-risk is calculated as:  ${P(BMD)}_{\%AR}=P\left( 0 \right)+ BMR$  ${P(BMD)}_{\%AR}=0.05+ 0.05$  ${P(BMD)}_{\%AR}=0.10 or 10\%$ added-risk  Step 3:  Using [8], the continuous outcome BMR_ΔSDy_ is calculated as:  ${BMR}_{DSDy}=f^{-1} \left[ P\left( 0 \right)]-f^{-1}[P(BMD) \right]=\left[ z_{@P\left( 0 \right)-}z_{@P\left( BMD \right)} \right]$  ${BMR}_{DSDy}=f^{-1} \left[ 0.05]-f^{-1}[0.10 \right]=\left[ z_{@0.05 -}z_{@0.10} \right]$  (Note: each z-value can be calculated with MS Excel ‘NORM.S.INV(probability)’ or Stata ‘display invnormal(probability)’, or R ‘qnorm(probability)’)  ${BMR}_{DSDy}=$ -1.645 – (-1.282)  $\boldsymbol{BMR}_{\boldsymbol{DSDy}}\boldsymbol{=}$ **-0.363 ≈** **-0.36SDy**  (Note: The continuous outcome BMR_ΔSDy_ of -36SDy calculated above for a binary outcome BMR of 5% added-risk with a background 5% “abnormal” matches the value reported in summary tables from Crump et al. (1995; 1998, 2000). See yellow row in Table S3A.3 below.)  Step 4:  Using [10], for IQ scores with a mean of µ_(0)_=100 and SDy=15, the equivalent BMR in IQ units is:  $\boldsymbol{BMR}_{\boldsymbol{Dy}}\boldsymbol{=}$ **-0.36 * 15 = -5.4 IQ points** |
| **Example 2:**  **unknown:**  BMR_ΔSDy_  BMR_Δy_  **provided:**  P(0)  BMR_%ER_  µ_(0)_  SDy | Similar to Example 1, but for a binary outcome BMR of 5% **extra**-risk.  The “hybrid approach” gives the equivalent continuous outcome BMR_ΔSDy_:  Step 1:  Specify the known parameters provided:  P(0) = 0.05 (i.e. 5%)  BMR_%ER_ = 0.05 (i.e. 5%)  Step 2:  Using [5] the P(BMD) % extra-risk is calculated as:  ${P(BMD)}_{\%ER}=P\left( 0 \right)+ BMR*[1-P\left( 0 \right)]$  ${P(BMD)}_{\%ER}=0.05+ 0.05*[1-0.05]$  ${P(BMD)}_{\%ER}=0.05+ 0.05*[0.95]$  ${P(BMD)}_{\%ER}=0.0975 or 9.75\%$ **extra**-risk  Step 3:  Using [8], the continuous outcome BMR_ΔSDy_ is calculated as:  ${BMR}_{DSDy}=f^{-1} \left[ P\left( 0 \right)]-f^{-1}[P(BMD) \right]=\left[ z_{@P\left( 0 \right)-}z_{@P\left( BMD \right)} \right]$  ${BMR}_{DSDy}=f^{-1} \left[ 0.05]-f^{-1}[0.0975 \right]=\left[ z_{@0.05 -}z_{@0.0975} \right]$  (Note: each z-value can be calculated with MS Excel ‘NORM.S.INV(probability)’, Stata ‘display invnormal(probability)’, or R ‘qnorm(probability)’)  ${BMR}_{DSDy}=$ -1.645 – (-1.296)  $\boldsymbol{BMR}_{\boldsymbol{DSDy}}\boldsymbol{=}$ **-0.349 ≈** **-0.35SDy**  (Note: The continuous outcome BMR_ΔSDy_ of -35SDy corresponding to a binary outcome BMR 5% extra-risk is nearly identical to the BMR_ΔSDy_ of -36SDy for 5% added-risk calculated in Example 1 – since the P(0) of 5% is a small value close to zero, there is only a very small difference between the BMR_ΔSDy_ for the equivalent BMR % added-risk or % extra-risk.)  Step 4:  Using [10], for IQ scores with a mean of µ_(0)_=100 and SDy=15, the equivalent BMR in IQ units is:  $\boldsymbol{BMR}_{\boldsymbol{Dy}}\boldsymbol{=}$ **-0.35 * 15 = -5.25 IQ points** |
| **Example 3:**  **unknown:**  BMR_ΔSDy_  µ_(BMD)_  C  BMR_%AR_  BMR_%ER_  **provided:**  P(0)  BMR_Δy_  µ_(0)_  SDy | Previous risk assessments of lead (Budtz-Jørgensen et al., 2013; EFSA, 2010) and fluoride (Grandjean et al., 2022) used a continuous outcome BMR_Δy_ of -1 IQ point based on a general population mean IQ of µ_(0)_ =100 and SDy=15. Since neither risk assessment used the “hybrid approach”, a background “abnormal” probability P(0) of 5% was assumed, which was consistent with the BMD modelling approach taken for the MeHg assessments by the NAS (2000) and US EPA (2001b).  For a continuous outcome BMR_ΔSDy_ of -0.067SDy, the “hybrid approach” gives the equivalent binary outcome **BMR % added-risk (BMR_%AR_)** or **BMR % extra-risk (BMR_%ER_)**:  Step 1:  Specify the known parameters provided:  BMR_Δy_ = -1 IQ point  P(0) = 0.05 (i.e. 5% in left tail or normal distribution)  µ_(0)_ = 100 (i.e. mean IQ in general “unexposed” population)  SDy = 15 (i.e. SD of mean IQ in general “unexposed” population)  Step 2:  From [9] substituted BMR_Δy_ into [7], BMR_ΔSDy_ is calculated as:  BMR_ΔSDy_ = \|µ_(BMD)_ - µ_(0)_\|/SDy = BMR_Δy_ / SDy  BMR_ΔSDy_ = -1 / 15  **BMR_ΔSDy_ = -0.067**  Step 3:  Rearranging [9] the mean outcome score at the BMD or µ_(BMD)_ is calculated as:  $\mu_{\left( BMD \right)}={BMR}_{Dy}+ \mu_{\left( 0 \right)}$  $\mu_{\left( BMD \right)}=-1+ 100$  $\boldsymbol{\mu}_{\left( \boldsymbol{BMD} \right)}\boldsymbol{=}\boldsymbol{99}$ **IQ points**  Step 4:  Rearranging [1], the value of the outcome above/below which defines “abnormal” (*C*) when background probability of an “abnormal” score P(0) is given, then *C* is calculated as:  $C= {(f}^{-1}\left[ P\left( 0 \right) \right]*SDy)+\mu_{(0)}$  $C= {(f}^{-1}\left[ 0.05 \right]*15)+100$  $C= {(f}^{-1}\left[ 0.05 \right]*15)+100$  (Note: $f^{-1}\left[ 0.05 \right]$ can be calculated as: MS Excel ‘NORM.S.INV(0.05)’, Stata ‘display invnormal(0.05)’, or R ‘qnorm(0.05)’)  $C= -1.64485*15+100$  $\boldsymbol{C= 75.327}$ **IQ score** as cut-off for “abnormal” IQ score corresponding to background “abnormal” probability P(0) of 5%  Step 5:  Using [6] the probability of an “abnormal” outcome at the BMD or P(BMD) is calculated as:  $P\left( BMD \right)=f\left[ \frac{C- \mu_{(BMD)}}{SDy} \right]= f\left[ z_{@P(BMD)} \right]$  $P\left( BMD \right)=f\left[ \frac{75.327 - 99}{15} \right]= f\left[ -1.5782 \right]$  (Note: $f\left[ -1.5782 \right]$ can be calculated as: MS Excel ‘NORM.S.DIST(-1.5782,TRUE)’, Stata ‘display normal(-1.5782)’, or R ‘pnorm(-1.5782)’)  $\boldsymbol{P}\left( \boldsymbol{BMD} \right)\boldsymbol{=0.05726}$ **(i.e. 5.726%** left-tail probability of having an “abnormal” IQ score ≤ C=75.33, when the sample mean IQ at the BMD is µ_(BMD)_ = 99 with SDy=15)    Step 6:  Step 6a: for BMR % added-risk or BMR_%AR_:  Using [2], the binary outcome BMR % added-risk is calculated as:  ${BMR}_{\%AR}=P\left( BMD \right)- P\left( 0 \right)$  ${BMR}_{\%AR}=0.05726-0.05$  $\boldsymbol{BMR}_{\boldsymbol{\%}\boldsymbol{AR}}\boldsymbol{=}\boldsymbol{0}\boldsymbol{.}\boldsymbol{00726}$ **or 0.73%** (see blue row in Table S3A.3 below.)  Step 6b: for BMR % extra-risk or BMR_%ER_:  Using [3], the binary outcome BMR % extra-risk is calculated as:  ${BMR}_{\%ER}=\frac{[P\left( BMD \right)- P\left( 0 \right)]}{[1-P\left( 0 \right)]}$  ${BMR}_{\%ER}=\frac{[0.05726- 0.05]}{[1-0.05]}$  $\boldsymbol{BMR}_{\boldsymbol{\%}\boldsymbol{ER}}\boldsymbol{=}\boldsymbol{0}\boldsymbol{.}\boldsymbol{00764}$ **or 0.76%**  (Note: The continuous outcome BMR_Δy_ of -1 IQ points used for risk assessments of lead and fluoride, corresponds to a “hybrid approach” binary outcome BMR of 0.73% added-risk or 0.76% extra-risk when the background “abnormal” probability P(0) is 5%. This corresponds to an abnormal IQ cut-off of 75.33 IQ points). |

The conversions in Table S3A.3 below are based on the methods in Tables S3A.1 and are an extension of similar tables published by Crump et al. (1995; 1998, 2000). More extensive BMR conversion tables using the same methods are provided in MS Excel worksheets in Supplemental File S3B)

**Table S3A.3 BMR “hybrid approach” conversion table for selected combinations of inputs and IQ score as an example outcome with “unexposed” population mean µ_(0)_ = 100 & SDy=15** (yellow row indicates the BMR used for methylmercury (NAS, 2000; US EPA, 2001b), blue row indicates BMR used for lead (Budtz-Jørgensen et al., 2013; EFSA, 2010) but with the same background “abnormal” P(0)=5% as for methylmercury since the “hybrid approach” was not used for lead)

| **Inputs** | **P(0)** | **binary BMR_%_** | **binary risk type** | **P(BMD)** | **z_@P(0)_** | **z_@P(BMD)_** | **continuous**  **BMR_ΔSDy_**  (z_@P(0)_ - z_@P(BMD)_) | **µ(BMD)**  **for IQ** | **continuous**  **BMR_Δy_**  **for IQ** | **C**  **(“abnormal” cut-off for IQ)** |
| --- | --- | --- | --- | --- | --- | --- | --- | --- | --- | --- |
| Varying the P(0) (1 or 5%) and binary BMR_%_ (1, 5, 10% added- or extra-risk) | **1.00%** | **1.00%** | extra | 1.99% | -2.326 | -2.056 | **-0.271** | 95.94 | **-4.06** | **65.10** |
|  | **1.00%** | **1.00%** | added | 2.00% | -2.326 | -2.054 | **-0.273** | 95.91 | **-4.09** | **65.10** |
|  | **1.00%** | **5.00%** | extra | 5.95% | -2.326 | -1.559 | **-0.767** | 88.49 | **-11.51** | **65.10** |
|  | **1.00%** | **5.00%** | added | 6.00% | -2.326 | -1.555 | **-0.772** | 88.43 | **-11.57** | **65.10** |
|  | **1.00%** | **10.00%** | extra | 10.90% | -2.326 | -1.232 | **-1.094** | 83.58 | **-16.42** | **65.10** |
|  | **1.00%** | **10.00%** | added | 11.00% | -2.326 | -1.227 | **-1.100** | 83.50 | **-16.50** | **65.10** |
|  | **5.00%** | **1.00%** | added | 6.00% | -1.645 | -1.555 | **-0.090** | 98.65 | **-1.35** | **75.33** |
|  | **5.00%** | **1.00%** | extra | 5.95% | -1.645 | -1.559 | **-0.086** | 98.71 | **-1.29** | **75.33** |
|  | **5.00%** | **5.00%** | added | 10.00% | -1.645 | -1.282 | **-0.363** | 94.55 | **-5.45** | **75.33** |
|  | **5.00%** | **5.00%** | extra | 9.75% | -1.645 | -1.296 | **-0.349** | 94.77 | **-5.23** | **75.33** |
|  | **5.00%** | **10.00%** | added | 15.00% | -1.645 | -1.036 | **-0.608** | 90.87 | **-9.13** | **75.33** |
|  | **5.00%** | **10.00%** | extra | 14.50% | -1.645 | -1.058 | **-0.587** | 91.20 | **-8.80** | **75.33** |
| Varying the z_@P(0)_ (1, 1.645, 2) and continuous BMR_ΔSDy_ (-0.067, -0.167, -0.333SDy which equates to a continuous BMR_Δy_ of -1, -2.5, or -5 IQ points) | **2.28%** | **0.38%** | added | 2.66% | -2.000 | -1.933 | **-0.067** | 99.00 | **-1.00** | **70.00** |
|  | **2.28%** | **0.39%** | extra | 2.66% | -2.000 | -1.933 | **-0.067** | 99.00 | **-1.00** | **70.00** |
|  | **5.00%** | **0.73%** | added | 5.72% | -1.645 | -1.578 | **-0.067** | 99.00 | **-1.00** | **75.33** |
|  | **5.00%** | **0.76%** | extra | 5.72% | -1.645 | -1.578 | **-0.067** | 99.00 | **-1.00** | **75.33** |
|  | **15.87%** | **1.67%** | added | 17.53% | -1.000 | -0.933 | **-0.067** | 99.00 | **-1.00** | **85.00** |
|  | **15.87%** | **1.98%** | extra | 17.53% | -1.000 | -0.933 | **-0.067** | 99.00 | **-1.00** | **85.00** |
|  | **2.28%** | **1.06%** | added | 3.34% | -2.000 | -1.833 | **-0.167** | 97.50 | **-2.50** | **70.00** |
|  | **2.28%** | **1.09%** | extra | 3.34% | -2.000 | -1.833 | **-0.167** | 97.50 | **-2.50** | **70.00** |
|  | **5.00%** | **1.97%** | added | 6.97% | -1.645 | -1.478 | **-0.167** | 97.50 | **-2.50** | **75.33** |
|  | **5.00%** | **2.07%** | extra | 6.97% | -1.645 | -1.478 | **-0.167** | 97.50 | **-2.50** | **75.33** |
|  | **15.87%** | **4.37%** | added | 20.23% | -1.000 | -0.833 | **-0.167** | 97.50 | **-2.50** | **85.00** |
|  | **15.87%** | **5.19%** | extra | 20.23% | -1.000 | -0.833 | **-0.167** | 97.50 | **-2.50** | **85.00** |
|  | **2.28%** | **2.50%** | added | 4.78% | -2.000 | -1.667 | **-0.333** | 95.00 | **-5.00** | **70.00** |
|  | **2.28%** | **2.56%** | extra | 4.78% | -2.000 | -1.667 | **-0.333** | 95.00 | **-5.00** | **70.00** |
|  | **5.00%** | **4.48%** | added | 9.48% | -1.645 | -1.312 | **-0.333** | 95.00 | **-5.00** | **75.33** |
|  | **5.00%** | **4.72%** | extra | 9.48% | -1.645 | -1.312 | **-0.333** | 95.00 | **-5.00** | **75.33** |
|  | **15.87%** | **9.38%** | added | 25.25% | -1.000 | -0.667 | **-0.333** | 95.00 | **-5.00** | **85.00** |
|  | **15.87%** | **11.15%** | extra | 25.25% | -1.000 | -0.667 | **-0.333** | 95.00 | **-5.00** | **85.00** |

**References**

Budtz-Jørgensen, E., Bellinger, D., Lanphear, B., Grandjean, P., & Investigators, on behalf of the I. P. L. S. (2013). An International Pooled Analysis for Obtaining a Benchmark Dose for Environmental Lead Exposure in Children. *Risk Analysis*, *33*(3), 450–461. https://doi.org/https://doi.org/10.1111/j.1539-6924.2012.01882.x

Budtz-Jørgensen, E., Grandjean, P., Keiding, N., White, R. F., & Weihe, P. (2000). Benchmark dose calculations of methylmercury-associated neurobehavioural deficits. *Toxicology Letters*, *112*–*113*, 193–199. https://doi.org/10.1016/s0378-4274(99)00283-0

Budtz-Jørgensen, E., Keiding, N., & Grandjean, P. (1999). *Benchmark Modeling of the Faroese Methylmercury Data. Final Report to the US EPA. Odense and Copenhagen, Denmark, 6 July, 1999. Research Report 99/5. Department of Biostatistics, University of Copenhagen.* https://hero.epa.gov/hero/index.cfm/reference/details/reference_id/3841176

Budtz-Jørgensen, E., Keiding, N., & Grandjean, P. (2001). Benchmark dose calculation from epidemiological data. *Biometrics*, *57*(3), 698–706. https://doi.org/10.1111/j.0006-341x.2001.00698.x

Coull, B. A., Mezzetti, M., & Ryan, L. M. (2003). A Bayesian hierarchical model for risk assessment of methylmercury. *Journal of Agricultural, Biological, and Environmental Statistics*, *8*(3), 253–270. https://doi.org/10.1198/1085711032291

Crump, K. S. (1995). Calculation of Benchmark Doses from Continuous Data. *Risk Analysis*, *15*(1), 79–89. https://doi.org/https://doi.org/10.1111/j.1539-6924.1995.tb00095.x

Crump, K. S. (2002). Critical Issues in Benchmark Calculations from Continuous Data. *Critical Reviews in Toxicology*, *32*(3), 133–153. https://doi.org/10.1080/20024091064200

Crump, K. S., Kjellström, T., Shipp, A. M., Silvers, A., & Stewart, A. (1998). Influence of prenatal mercury exposure upon scholastic and psychological test performance: benchmark analysis of a New Zealand cohort. *Risk Analysis : An Official Publication of the Society for Risk Analysis*, *18*(6), 701–713. https://doi.org/10.1023/b:rian.0000005917.52151.e6

Crump, K. S., Van Landingham, C., Shamlaye, C., Cox, C., Davidson, P. W., Myers, G. J., & Clarkson, T. W. (2000). Benchmark concentrations for methylmercury obtained from the Seychelles Child Development Study. *Environmental Health Perspectives*, *108*(3), 257–263. https://doi.org/10.1289/ehp.00108257

EFSA. (2010). *Scientific Opinion on Lead in Food*. https://efsa.onlinelibrary.wiley.com/doi/epdf/10.2903/j.efsa.2010.1570

EFSA. (2012). Scientific Opinion on the risk for public health related to the presence of mercury and methylmercury in food. *EFSA Journal*, *10*(12). https://doi.org/10.2903/j.efsa.2012.2985

EFSA. (2017). Update: use of the benchmark dose approach in risk assessment. In *EFSA Journal* (Vol. 15, Issue 1). https://efsa.onlinelibrary.wiley.com/doi/epdf/10.2903/j.efsa.2017.4658

EFSA. (2022). *Guidance on the use of the benchmark dose approach in risk assessment. (Published 25 October 2022)*. https://efsa.onlinelibrary.wiley.com/doi/epdf/10.2903/j.efsa.2022.7584

Grandjean, P., Hu, H., Till, C., Green, R., Bashash, M., Flora, D., Tellez-Rojo, M. M., Song, P. X. K., Lanphear, B., & Budtz-Jørgensen, E. (2022). A Benchmark Dose Analysis for Maternal Pregnancy Urine-Fluoride and IQ in Children. *Risk Analysis : An Official Publication of the Society for Risk Analysis*, *42*(3), 439–449. https://doi.org/10.1111/risa.13767

Haber, L. T., Dourson, M. L., Allen, B. C., Hertzberg, R. C., Parker, A., Vincent, M. J., Maier, A., & Boobis, A. R. (2018). Benchmark dose (BMD) modeling: current practice, issues, and challenges. *Critical Reviews in Toxicology*, *48*(5), 387–415. https://doi.org/10.1080/10408444.2018.1430121

NAS. (2000). Toxicological Effects of Methylmercury. In *Toxicological Effects of Methylmercury (National Academy of Sciences)*. National Academies Press. https://doi.org/10.17226/9899

Sand, S., Victorin, K., & Filipsson, A. F. (2008). The current state of knowledge on the use of the benchmark dose concept in risk assessment. *Journal of Applied Toxicology : JAT*, *28*(4), 405–421. https://doi.org/10.1002/jat.1298

Suwazono, Y., Nogawa, K., Uetani, M., Miura, K., Sakata, K., Okayama, A., Ueshima, H., Stamler, J., & Nakagawa, H. (2011). Application of hybrid approach for estimating the benchmark dose of urinary cadmium for adverse renal effects in the general population of Japan. *Journal of Applied Toxicology : JAT*, *31*(1), 89–93. https://doi.org/10.1002/jat.1582

US EPA. (2001a). Methylmercury (MeHg) (CASRN 22967-92-6). I. Chronic Health Hazard Assessments for Noncarcinogenic Effects. I.A. Reference Dose for Chronic Oral Exposure (RfD). Last Revised 07/27/2001. Integrated Risk Information System (IRIS). Chemical Assessment Summary. In *Integrated Risk Information System (IRIS) Chemical Assessment Summary National Center for Environmental Assessment*. https://iris.epa.gov/static/pdfs/0073_summary.pdf

US EPA. (2001b). *Water Quality Criterion for the Protection of Human Health: Methylmercury. Final. (January 2001, EPA 823-R-01-001). Office of Science and Technology Office of Water U.S. Environmental Protection Agency Washington, DC 20460*. https://doi.org/https://www.epa.gov/sites/default/files/2020-01/documents/methylmercury-criterion-2001.pdf

US EPA. (2025a). *BMDS Training Videos: Selecting Continuous BMR*. https://www.epa.gov/bmds/bmds-training-videos#contbmr

US EPA. (2025b). *BMDS Training Videos: Selecting Dichotomous BMR*. https://www.epa.gov/bmds/bmds-training-videos#dichbmr

1. binary BMR definitions: *added-* or *additional-*risk (BMR_AR_ = [P(BMD) – P(0)]) or as an *extra*-risk (BMR_ER_ = [P(BMD) – P(0)]/[1 – P(0)]) (EFSA, 2017; Haber et al., 2018) [↑](#footnote-ref-1)
2. relative deviation (RD) BMR is a specified change in the mean of the continuous outcome relative to the mean of the outcome mean at background exposure level where %RD = |µ_(BMD)_ - µ_(0)_|/ µ_(0)_ *100 ; standard deviation BMR is a specified change in the mean of the continuous outcome (ΔSDy) relative to standard deviation of the outcome mean at background exposure level or BMR_ΔSDy_ = |µ_(BMD)_ - µ_(0)_|/ SDy_(0)_ [↑](#footnote-ref-2)
3. *added*-risk is also called *additional*-risk by some organizations [↑](#footnote-ref-3)
4. The NAS report (2000) used the term *excess* risk to refer to the more commonly used terms of either *added* or *additional* risk [↑](#footnote-ref-4)
5. Note that the original BMD modelling for the New Zealand cohort (Crump et al., 1998) and Seychelles Main cohort at 5.5yrs (Crump et al., 2000) reported using a binary outcome BMR of 10% added risk with a background abnormal probability P(0) of 5%, which corresponded to a continuous outcome BMR of 0.61SDy. In order to be consistent with BMD modelling for the Faroe Islands, the NAS requested Crump et al. to provide updated BMD modelling instead using the same binary outcome BMR of 5% added-risk which were used for the BMD estimates reported (NAS, 2000). [↑](#footnote-ref-5)
6. Diagnostic & Statistical Manual of Mental Disorders (DSM-V) Library – Neurodevelopmental Disorders: <https://psychiatryonline.org/doi/full/10.1176/appi.books.9780890425787.x01_Neurodevelopmental_Disorders> [↑](#footnote-ref-6)
