# Supplementary material for: Neurodevelopmental effects of methylmercury (MeHg): a review of epidemiological points of departure (PoDs), toxicological reference values (TRVs), and key uncertainties in human health risk assessment
Source: Arch Toxicol. 2026 Mar 10;100(6):2191–219. doi: 10.1007/s00204-026-04345-8 (PMC13221417; doi:10.1007/s00204-026-04345-8)
Supplement: Supplementary file 5 — Supplementary file5 (DOCX 510 kb) [file 204_2026_4345_MOESM5_ESM.docx]

**Supplemental File S4A. Validation of the Meta-Analysis by Axelrad et al. 2007**

*Archives of Toxicology*

Neurodevelopmental effects of methylmercury (MeHg): A review of epidemiological points of departure (PoDs), toxicological reference values (TRVs), and key uncertainties in human health risk assessment

Blechinger, Scott R.^1^* (ORCID 0000-0002-4991-4597, Scopus ID 6506155596)

Singh, Kavita^2^ (ORCID N/A, Scopus ID 58382466900)

Afghan, Abdul^1^ (ORCID N/A, Scopus ID 58522410100)

Smith, Catherine A.^1^ (ORCID N/A, Scopus ID 46461849300)

^1^ Bureau of Chemical Safety, Food and Nutrition Directorate, Health Canada, Ottawa, Canada

^2^ Environmental Health Science and Research Bureau, Health Canada, Ottawa, Canada

*Corresponding author: scott.blechinger@hc-sc.gc.ca

The analysis by Axelrad et al. (2007) used a "Bayesian hierarchical model" or "hierarchical random-effects model that included both study-to-study and endpoint-to-endpoint variability" and employing a Markov Chain Monte Carlo Bayesian approach using WinBUGS (p.611 of Axelrad et al. 2007). The data used in the meta-analysis by Axelrad et al. are shown in Table S4A.1 and included 13 ^[[1]](#footnote-2)^ regression coefficients (β) from 3 cohorts, rescaled and expressed as a change in test score (IQ units) for an increase in 1 µg/g maternal hair THg.

**Table S4A.1 Rescaled regression coefficients (ΔIQ score for an increase in 1 µg/g maternal hair THg) used for the meta-analysis adapted from Table 3 in Axelrad et al. (2007)** (MS Excel file for import to R found in Supplemental File S4B.1)

| est_id | cohort | authyr | ageyr | n | domain | test | beta | se | v | plot_ order |
| --- | --- | --- | --- | --- | --- | --- | --- | --- | --- | --- |
| 1 | Faroe | Budtz-Jorgensen_2005 | 7 | 917 | general intelligence | WISC-R FIQ (composite) | -0.124 | 0.057 | 0.00325 | 3 |
| 2 | Faroe | Budtz-Jorgensen_2005 | 7 | 917 | visual-motor integration | Bender (copy errors) | -0.104 | 0.083 | 0.00689 | 4 |
| 3 | Faroe | Budtz-Jorgensen_2005 | 7 | 917 | confrontational naming | BNT (no cues) | -0.260 | 0.086 | 0.00740 | 1 |
| 4 | Faroe | Budtz-Jorgensen_2005 | 7 | 917 | verbal learning and memory | CVLT (short-term recall) | -0.169 | 0.093 | 0.00865 | 2 |
| 5 | New_Zealand | Crump_1998 | 6 | 237 | general intelligence | WISC-R FIQ | -0.500 | 0.268 | 0.07182 | 7 |
| 6 | New_Zealand | Crump_1998 | 6 | 237 | general intelligence | WISC-R PIQ | -0.510 | 0.310 | 0.09610 | 8 |
| 7 | New_Zealand | Crump_1998 | 6 | 237 | general verbal skills | TOLD (spoken language) | -0.560 | 0.282 | 0.07952 | 6 |
| 8 | New_Zealand | Crump_1998 | 6 | 237 | general development | MSCA (perceptual) | -0.800 | 0.315 | 0.09923 | 5 |
| 9 | Seychelles | Myers_2003 | 9 | 643 | general intelligence | WISC-III FIQ | -0.170 | 0.130 | 0.01690 | 9 |
| 10 | Seychelles | Myers_2003 | 9 | 643 | verbal learning and memory | CVLT (short-term recall) | 0.190 | 0.144 | 0.02074 | 13 |
| 11 | Seychelles | Myers_2003 | 9 | 643 | confrontational naming | BNT (total score) | -0.038 | 0.144 | 0.02074 | 11 |
| 12 | Seychelles | Myers_2003 | 9 | 643 | visual memory | WRAML (design memory) | -0.109 | 0.150 | 0.02250 | 10 |
| 13 | Seychelles | Myers_2003 | 9 | 643 | visual-motor integration | VMI | -0.013 | 0.150 | 0.02250 | 12 |

Axelrad et al. (2007) reported 3 sources of variation in their model: sampling error (SE), between-cohort^[[2]](#footnote-3)^ heterogeneity (σ_cohort_) as a random effect, and between-domain^[[3]](#footnote-4)^ heterogeneity (σ_domain_) as a random effect; a 4^th^ source of variation, between-estimate heterogeneity (σ_estimate_), which is standard in every RE model was not reported. Table S4A.2 below shows the pooled estimate of the regression coefficient (β_pooled_), its standard error (SE) and random effects extracted from the “primary analysis” row in Table 5 of Axelrad et al. (2007). Also shown are columns for calculated 90%^[[4]](#footnote-5)^ confidence interval (CI) and 90% prediction interval (PI). In random-effects meta-analyses, the PI is considered the best metric for characterizing the heterogeneity, or degree of dispersion, of the true effect being investigated and it is recommended that the PI is always reported along with the CI (Borenstein, 2023; Botella & Sánchez-Meca, 2024; IntHout et al., 2016). In random effects meta-analysis models, the CI represents an interval within which the average effect will fall under repeated theoretical samples, while the PI contains a probable range for the effect that could observed in future studies. The PI is generally wider than the CI since the PI is a function of the variance from SE as well as variance from the random effect(s) as shown in equations [1] and [2] below, which have been adapted and simplified from published sources (Borenstein, 2023; Botella & Sánchez-Meca, 2024; IntHout et al., 2016).

$90\% CI= \beta_{pooled} \pm\left( z or t_{df} \right)*(SE)$ [1]

where:

*β_pooled_* = average regression coefficient from the meta-analysis

*z* = from the standard normal distribution corresponding to a given % CI or PI i.e. z=1.645 for 90%CI or PI, can be computed from MS Excel by ‘=ABS(NORM.S.INV((1-0.90)/2))’

*t_df_* = value from t-distribution corresponding to a given % CI or PI and specific degrees of freedom *df*, where df may be defined as either *k*-1 or *k*-2 and k is the number of estimates (*k* may also be the smallest value of grouping/cluster for nested random effects) i.e. t=1.782 for df=12 with 90% CI or PI can be computed from MS Excel using ‘=T.INV.2T(1-0.90,12)’

*SE* = standard error of the mean pooled estimate β_pooled_ (also called the square root of the variance of the mean estimate)

$90\% PI= \beta_{pooled} \pm\left( z or t_{df} \right)*{SD}_{PI}$ [2]

where:

*β_pooled_* = same definitions as in [1]

*z* = same definitions as in [1]

*t_df_* = same definitions as in [1]

${SD}_{PI}= \sqrt{({SE}^{2}+ \sum{\sigma_{random effect}}^{2})}$ = sometimes called the ‘standard deviation of the prediction interval’ or SD_PI_ (IntHout et al., 2016) is the square root of the sum of the variance for the sampling error and sum of the variance for random effects in the model (*∑σ_random effect_^2^*). For example, a standard random effects model, sometimes called a 2-level model, will only have a single variance σ^2^ for between-estimate heterogeneity σ_estimate_^2^, often called tau^2^ or τ^2^, which gives the SD_PI_ = $\sqrt{({SE}^{2}+ {\sigma_{estimate}}^{2})}$ or also called $\sqrt{({SE}^{2}+ \tau^{2})}$. However, more complex models including hierarchical random effects models will additionally include σ^2^ for each higher level nested random effect so there will be 2 separate values of σ^2^ for a 3-level model, and 3 σ^2^ for a 4-level model, etc. (Harrer et al., 2021). In addition, any non-nested random effects will also have a σ^2^. For a standard random effects model also called a 2-level model, there is only one variance estimate (σ_estimate_^2^) for between-estimate heterogeneity, which gives the SD_PI_ = $\sqrt{({SE}^{2}+ {\sigma_{estimate}}^{2})}$, also called $\sqrt{({SE}^{2}+ \tau^{2})}$. However, for hierarchical random effects models with 3 or more nested levels and/or non-nested additional random effects, multiple variances estimates (σ^2^) could be summed as part of the calculation for SD_PI_. In the analysis by Axelrad et al. (2007), there is some uncertainty in how the random effects are structured, but presumably there should be at least 3 random effects: between-estimate heterogeneity (σ_estimate_^2^), between-cohort heterogeneity (σ_cohort_^2^), and between-domain heterogeneity (σ_domain_^2^). Therefore, together with the variance for sampling error, the standard deviation of the PI could be calculated as:

${SD}_{PI}= \sqrt{({SE}^{2}+\left( {\sigma_{estimate}}^{2}+{\sigma_{cohort}}^{2}+{\sigma_{domain}}^{2} \right))}$ [3]

However, it should be noted that Axelrad et al. (2007) only explicitly reported the between-cohort heterogeneity as the square root σ_cohort_= 0.112, which can be squared to give the variance σ_cohort_^2^ = 0.0125. The between-domain heterogeneity variance was not reported, but calculated in this review as σ_domain_^2^ = (σ_cohort_^2^/R) = (0.0125/3.0) = 0.0042 or by taking the square root to give σ_domain_ = 0.0648, where R=3.0 when σ_cohort_= 0.112 from Table 4 of Axelrad et al. (2007). The variance for the between-estimate heterogeneity (σ_estimate_^2^) was not reported in Axelrad et al. (2007) and therefore could not be used when calculating SD_PI_.

In this validation exercise, the same Axelrad et al. data from Table S4A.1 was used to conduct a frequentist multi-level meta-analysis with the ‘**rma.mv**’ and **‘predict.rma’** functions from the **{metafor}** R-package (Viechtbauer, 2010). The pooled estimate, SE, and random effect variance from this frequentist multi-level meta-analysis was used to validate the corresponding meta-analysis pooled estimates reported in Axelrad et al. (2007) based on a Bayesian approach. The pooled estimate from both Axelrad et al. (2007) and the ‘**rma.mv**’ function from **{metafor}** are shown in Table S4A.2 along with the square-root of the variance for the sampling error and random effects, the 95% and 90% CI’s and corresponding PI’s generated post-hoc with the **‘predict.rma’** function, and forest plots visualized using the **‘forest.rma’** function from **{metafor}**. Specific R-script for this analyses is provided in the Appendix of this file. The probability of either an adverse (β<0) or beneficial (β>0) regression coefficient in a future study was estimated from the predictive distribution using β_pooled_, SD_PI_, and the normal distribution function in MS Excel:

Probability adverse future study (β<0): ‘=NORM.DIST(0, β_pooled_, SD_PI_ ,TRUE)’ [4]

Probability of beneficial future study (β>0): ‘=1 - NORM.DIST(0, β_pooled_, SD_PI_ ,TRUE)’ [5]

**Table S4A2. Pooled regression coefficients from meta-analysis by Axelrad et al. (2007) and the ‘rma.mv’ function from {metafor}** (MS Excel file showing calculations in Supplemental File S4B.2)

| **Method/ Source** | **β_pooled_** | **SE** | **σ_estimate_**  **(sigma^2.2 in R)** | **σ_cohort_**  **(sigma^2.1 in R)** | **σ_domain_**  **(sigma^2.3 in R)** | **SD_PI_**  **(using Eq [3])** | **z or t** | **df ^c^** | **critical value**  **(z or t)** | **% interval** | **CI of β_pooled_**  **(using Eq [1])** | | **PI of β_pooled_**  **(using Eq [2])** | | **Prob.% of adverse future study ^d^** | **Prob. % of beneficial future study ^d^** |
| --- | --- | --- | --- | --- | --- | --- | --- | --- | --- | --- | --- | --- | --- | --- | --- | --- |
|  |  |  |  |  |  |  |  |  |  |  | lower | upper | lower | upper |  |  |
| Axelrad et al. (2007) Table 5 | -0.180 | 0.092 | 0 ^a^ | 0.112 | 0.0648 | 0.1588 | z | . | 1.960 | 95% | -0.360 | 0.000 | -0.491 | 0.131 | 87.15% | 12.85% |
|  | -0.180 | 0.092 | 0 ^a^ | 0.112 | 0.0648 | 0.1588 | z | . | 1.645 | 90% | -0.331 | -0.029 | -0.441 | 0.081 | 87.15% | 12.85% |
|  | -0.180 | 0.092 | 0 ^a^ | 0.112 | 0.0648 | 0.1588 | t | 12 | 2.179 | 95% | -0.380 | 0.020 | -0.526 | 0.166 | 87.15% | 12.85% |
|  | -0.180 | 0.092 | 0 ^a^ | 0.112 | 0.0648 | 0.1588 | t | 12 | 1.782 | 90% | -0.344 | -0.016 | -0.463 | 0.103 | 87.15% | 12.85% |
| Model 1 from {metafor} Appendix S4A.A | -0.231 | 0.1522 | 0 ^b^ | 0.2481 | 0.000002 | 0.2911 | z | . | 1.960 | 95% | -0.529 | 0.067 | -0.802 | 0.340 | 78.63% | 21.37% |
|  | -0.231 | 0.1522 | 0 ^b^ | 0.2481 | 0.000002 | 0.2911 | z | . | 1.645 | 90% | -0.481 | 0.019 | -0.710 | 0.248 | 78.63% | 21.37% |
| Model 2 from {metafor} Appendix S4A.A | -0.231 | 0.1522 | 0 ^b^ | 0.2481 | 0.000002 | 0.2911 | t | 12 | 2.179 | 95% | -0.563 | 0.101 | -0.865 | 0.403 | 78.6% | 21.37% |
|  | -0.231 | 0.1522 | 0 ^b^ | 0.2481 | 0.000002 | 0.2911 | t | 12 | 1.782 | 90% | -0.502 | 0.040 | -0.750 | 0.288 | 78.6% | 21.37% |

^a^ not reported in Axelrad et al. (2007)

^b^ estimated in Models 1 and 2 with ‘rma.mv’ function in {metafor} but was estimated to be <0.000000, so recorded as “0” (see model output in Appendix S4A.A)

^c^ df (degrees of freedom) = k – 1 = 13 -1 = 12 (where k= 13, the number of regression coefficients modelled, from Table S4A.1)

^d^ using equations [4] or [5] respectively

**Supplemental File S4A. Appendix**

**R code and results for the frequentist meta-analysis using ‘rma.mv’ function from the {metafor} R package**

All RE models below are hierarchical 3-level models with level 1 (participants) nested in level 2 (aggregated summary estimates) nested in level 3 (cohorts) and an additional un-nested random effect (domain). The model includes 4 sources of variability (Harrer et al., 2021):

- sampling error (level 1)
- between estimate heterogeneity (level 2 random effect)
- between cohort heterogeneity (level 3 random effect)
- between domain heterogeneity (non-nested random effect)

The analysis below was conducted in R version 4.4.3 (2025-02-28 ucrt) using RStudio 2024.12.1 Build 563.

Greyed text represent code blocks with output as normal text.

# Set Up

| # load libraries  library (readxl) # for reading excel files into R  library (tidyverse) # for basic data manipulation functions  library (metafor) # for meta-analysis including forest plots |
| --- |

| # import dataset and rename: Axelrad_2007 Table 3.xlsx  axel <- read_excel("[user’s directory]/Axelrad_2007 Table 3.xlsx")  View(axel) |
| --- |

# Three-Level Random Effects (RE) Models

## Model 1: Sampling error , CI’s, and PI’s estimated using the z-distribution

### Model output

| # multi-level random effects model, estimates nested in cohorts, and domain not nested but as an independent random effect  # SE calculated using z-distribution  model1 <- rma.mv(yi=beta, V=v, data=axel,  random = list(~1 \| cohort/est_id, ~1 \| domain),  slab=paste0(est_id),  level=95,  test="z",  method="REML",  digits=6)  summary(model1) |
| --- |

Multivariate Meta-Analysis Model (k = 13; method: REML)

logLik Deviance AIC BIC AICc

4.818293 -9.636585 -1.636585 0.303042 4.077701

Variance Components:

estim sqrt nlvls fixed factor

sigma^2.1 0.061573 0.248140 3 no cohort

sigma^2.2 0.000000 0.000002 13 no cohort/est_id

sigma^2.3 0.000000 0.000000 7 no domain

Test for Heterogeneity:

Q(df = 12) = 18.697383, p-val = 0.096098

Model Results:

estimate se zval pval ci.lb ci.ub

-0.231036 0.152194 -1.518037 0.129005 -0.529330 0.067259

---

Signif. codes: 0 ‘***’ 0.001 ‘**’ 0.01 ‘*’ 0.05 ‘.’ 0.1 ‘ ’ 1

### Predictions of CI’s and PI’s

#### 95% CI’s and PI’s

| # 95% CI's and PI's from model 1 calculated using z=1.96 for 95% CI/PI (2.5% in each tail)  model1.pred <- predict.rma(model1, level=95)  model1.pred |
| --- |

pred se ci.lb ci.ub pi.lb pi.ub

-0.231036 0.152194 -0.529330 0.067259 -0.80157 0.339500

| # report the standard deviation used for calculating the 95% PI  model1.pred95$pi.se |
| --- |

0.291095

#### 90% CI’s and PI’s

| # 90% CI's and PI's from model 1 calculated using z=1.645 for 90% CI/PI (5% in each tail)  model1.pred90 <- predict.rma(model1, level=90)  model1.pred90 |
| --- |

pred se ci.lb ci.ub pi.lb pi.ub

-0.231036 0.152194 -0.481372 0.019301 -0.709844 0.247773

| # report the standard deviation used for calculating the 90% PI  model1.pred90$pi.se |
| --- |

0.291095

### Predictive probabilities of a future estimate in adverse or beneficial direction

| # probability of adverse regression coefficient in a future study, to validate the same calculation in MS Excel  100*pnorm(0,mean=model1$beta,sd=model1.pred95$pi.se, lower.tail=TRUE) |
| --- |

78.63084

| # probability of adverse regression coefficient in a future study, to validate the same calculation in MS Excel  100*pnorm(0,mean=model1$beta,sd=model1.pred95$pi.se, lower.tail=FALSE) |
| --- |

21.36916

### Forest plot

Pooled estimate with 90% CI’s shown as grey diamond and 90% PI’s as a distribution (5% in each tail are shown in black with the middle 90% as red for adverse direction and/or blue for beneficial direction). Regression coefficients were reordered first by cohort, then in order of most to least adverse.

| # Model 1 forest plot  forest.rma(model1, order=order,  header="Est.", xlab ="Change in Cognitive Score (IQ units)",  plim=c(1.2,1.2), alim=c(-1,1), efac=c(0,2,2,1),  xlim=c(-5,2), digits=3, cex=0.6,  showweights="rowsum", # reporting the model weights using showweights="rowsum"  ilab=cbind(cohort,authyr,ageyr,domain,test),  ilab.lab=c("Cohort","Author_Year","Age","Domain","Test"),  ilab.xpos=c(-4.8,-4.2,-3.3,-3,-2),  ilab.pos=c(4),  level=90,predstyle="dist", col=c("darkgrey","black","#43f7fc","#fc5743")) |
| --- |


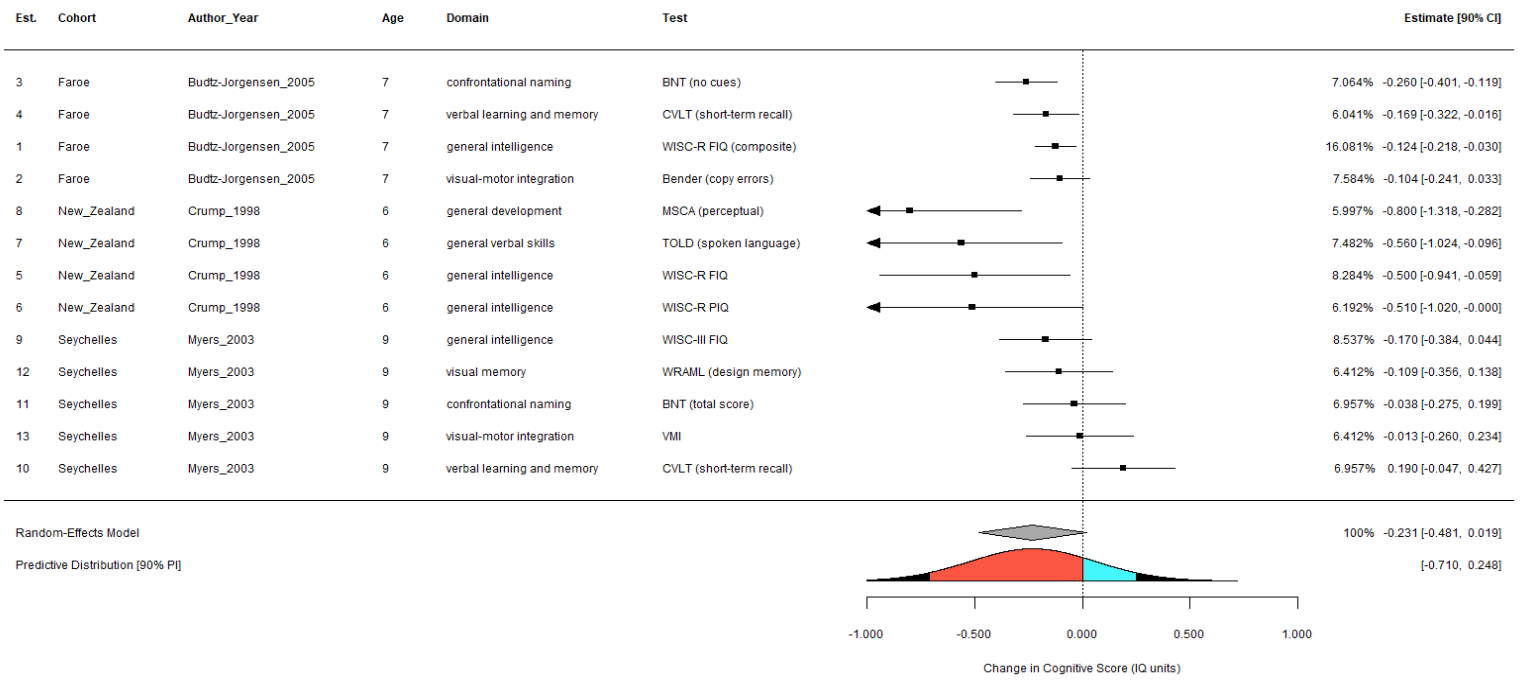


## Model 2: Sampling error , CI’s, and PI’s estimated using the t-distribution with df=13-1=12

### Model output

| # multi-level random effects model, estimates nested in cohorts, and domain not nested but as an independent random effect  # SE calculated using t-distribution  model2 <- rma.mv(yi=beta, V=v, data=axel,  random = list(~1 \| cohort/est_id, ~1 \| domain),  slab=paste0(est_id),  level=95,  test="t",  method="REML",  digits=6)  summary(model2) |
| --- |

Multivariate Meta-Analysis Model (k = 13; method: REML)

logLik Deviance AIC BIC AICc

4.818293 -9.636585 -1.636585 0.303042 4.077701

Variance Components:

estim sqrt nlvls fixed factor

sigma^2.1 0.061573 0.248140 3 no cohort

sigma^2.2 0.000000 0.000002 13 no cohort/est_id

sigma^2.3 0.000000 0.000000 7 no domain

Test for Heterogeneity:

Q(df = 12) = 18.697383, p-val = 0.096098

Model Results:

estimate se tval df pval ci.lb ci.ub

-0.231036 0.152194 -1.518037 12 0.154902 -0.562637 0.100566

---

Signif. codes: 0 ‘***’ 0.001 ‘**’ 0.01 ‘*’ 0.05 ‘.’ 0.1 ‘ ’ 1

### Predictions of CI’s and PI’s

#### 95% CI’s and PI’s

| # 95% CI's and PI's from model 1 calculated using t=2.179 with df=12 for 95% CI/PI (2.5% in each tail)  model2.pred <- predict.rma(model1, level=95)  model2.pred |
| --- |

pred se ci.lb ci.ub pi.lb pi.ub

-0.231036 0.152194 -0.562637 0.100566 -0.865277 0.403206

| # report the standard deviation used for calculating the 95% PI  model2.pred95$pi.se |
| --- |

0.291095

#### 90% CI’s and PI’s

| # 90% CI's and PI's from model 1 calculated using t=1.782 with df=12 for 90% CI/PI (5% in each tail)  model2.pred90 <- predict.rma(model1, level=90)  model2.pred90 |
| --- |

pred se ci.lb ci.ub pi.lb pi.ub

-0.231036 0.152194 -0.502288 0.040217 -0.749851 0.287779

| # report the standard deviation used for calculating the 90% PI  model2.pred90$pi.se |
| --- |

0.291095

### Predictive probabilities of a future estimate in adverse or beneficial direction

| # probability of adverse regression coefficient in a future study, to validate the same calculation in MS Excel  100*pnorm(0,mean=model2$beta,sd=model2.pred95$pi.se, lower.tail=TRUE) |
| --- |

78.63084

| # probability of adverse regression coefficient in a future study, to validate the same calculation in MS Excel  100*pnorm(0,mean=model2$beta,sd=model2.pred95$pi.se, lower.tail=FALSE) |
| --- |

21.36916

### Forest plot

Pooled estimate with 90% CI’s shown as grey diamond and 90% PI’s as a distribution (5% in each tail are shown in black with the middle 90% as red for adverse direction and/or blue for beneficial direction). Regression coefficients were reordered first by cohort, then in order of most to least adverse.

| # Model 2 forest plot  forest.rma(model2, order=order,  header="Est.", xlab ="Change in Cognitive Score (IQ units)",  plim=c(1.2,1.2), alim=c(-1,1), efac=c(0,2,2,1),  xlim=c(-5,2), digits=3, cex=0.6,  showweights="rowsum", # reporting the model weights using showweights="rowsum"  ilab=cbind(cohort,authyr,ageyr,domain,test),  ilab.lab=c("Cohort","Author_Year","Age","Domain","Test"),  ilab.xpos=c(-4.8,-4.2,-3.3,-3,-2),  ilab.pos=c(4),  level=90,predstyle="dist", col=c("darkgrey","black","#43f7fc","#fc5743")) |
| --- |


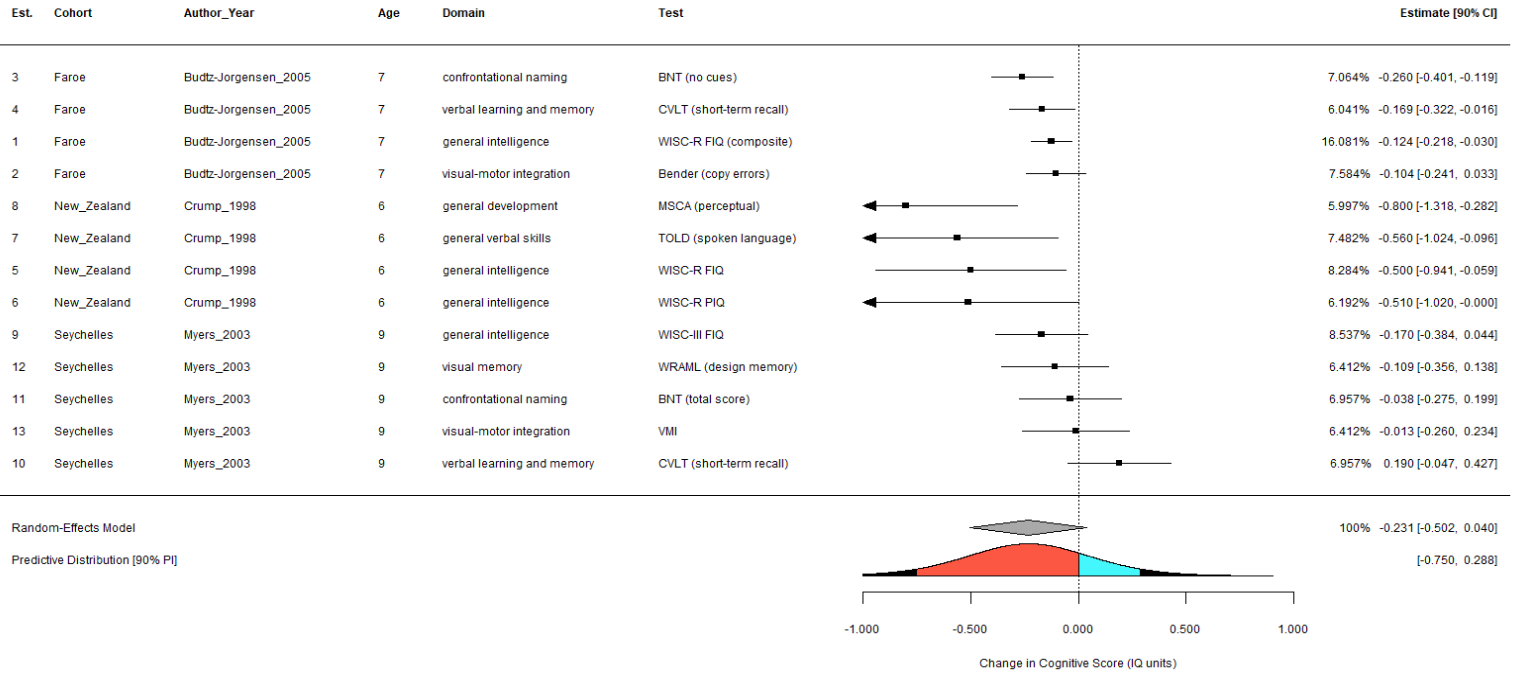


**References**

Axelrad, D. A., Bellinger, D. C., Ryan, L. M., & Woodruff, T. J. (2007). Dose-response relationship of prenatal mercury exposure and IQ: an integrative analysis of epidemiologic data. *Environmental Health Perspectives*, *115*(4), 609–615. https://ehp.niehs.nih.gov/doi/10.1289/ehp.9303

Borenstein, M. (2023). How to understand and report heterogeneity in a meta-analysis: The difference between I-squared and prediction intervals. *Integrative Medicine Research*, *12*(4), 101014. https://doi.org/https://doi.org/10.1016/j.imr.2023.101014

Botella, J., & Sánchez-Meca, J. (2024). Meta-análisis: Intervalos de confianza e Intervalos de predicción. *Anales de Psicología / Annals of Psychology*, *40*(2 SE-Metodología), 344–354. https://doi.org/10.6018/analesps.591831

Harrer, M., Cuijpers, P., Furukawa, T. A., & Ebert, D. D. (2021). *Doing Meta-Analysis With R: A Hands-On Guide* (1st ed.). Chapman & Hall/CRC Press. https://www.routledge.com/Doing-Meta-Analysis-with-R-A-Hands-On-Guide/Harrer-Cuijpers-Furukawa-Ebert/p/book/9780367610074

IntHout, J., Ioannidis, J. P. A., Rovers, M. M., & Goeman, J. J. (2016). Plea for routinely presenting prediction intervals in meta-analysis. *BMJ Open*, *6*(7), e010247. https://doi.org/10.1136/bmjopen-2015-010247

Viechtbauer, W. (2010). Conducting Meta-Analyses in R with the metafor Package. *Journal of Statistical Software*, *36*(3 SE-Articles), 1–48. https://doi.org/10.18637/jss.v036.i03

1. For Faroe Cohort 1, 6 regression coefficients were available for the meta-analysis but of these 3 coefficients for WISC-R subscale tests were combined to give a single composite WISC-R FIQ coefficient which was actually used in the meta-analyses. Therefore, a total of 15 coefficients were available (6 Faroe + 4 New Zealand + 5 Seychelles) but a total of 13 coefficients (12 + 1 composite) were used in the met-analysis (4 Faroe + 4 New Zealand + 5 Seychelles) [↑](#footnote-ref-2)
2. referred to “cohort” here, but called “study” in Axelrad et al. (2007) [↑](#footnote-ref-3)
3. referred to as “domain” here, but called “endpoint” in Axelrad et al. (2007) [↑](#footnote-ref-4)
4. 90% CI’s and PI’s were used here to be in line with the standard approach for calculating the BMDL and BMDU corresponding to the lower and upper 5% of the distribution. [↑](#footnote-ref-5)
