# Supplementary material for: Neurodevelopmental effects of methylmercury (MeHg): a review of epidemiological points of departure (PoDs), toxicological reference values (TRVs), and key uncertainties in human health risk assessment
Source: Arch Toxicol. 2026 Mar 10;100(6):2191–219. doi: 10.1007/s00204-026-04345-8 (PMC13221417; doi:10.1007/s00204-026-04345-8)
Supplement: Supplementary file 8 — Supplementary file8 (DOCX 245 kb) [file 204_2026_4345_MOESM8_ESM.docx]

**Supplemental File S6A**

**BMD Modelling of Child Neurological Scores from the Iraq Poisoning (Marsh et al., 1987)**

*Archives of Toxicology*

Neurodevelopmental effects of methylmercury (MeHg): A review of epidemiological points of departure (PoDs), toxicological reference values (TRVs), and key uncertainties in human health risk assessment

Blechinger, Scott R.^1^* (ORCID 0000-0002-4991-4597, Scopus ID 6506155596)

Singh, Kavita^2^ (ORCID N/A, Scopus ID 58382466900)

Afghan, Abdul^1^ (ORCID N/A, Scopus ID 58522410100)

Smith, Catherine A.^1^ (ORCID N/A, Scopus ID 46461849300)

^1^ Bureau of Chemical Safety, Food and Nutrition Directorate, Health Canada, Ottawa, Canada

^2^ Environmental Health Science and Research Bureau, Health Canada, Ottawa, Canada

*Corresponding author: scott.blechinger@hc-sc.gc.ca

# The Data

Clinicians conducted a neurological exam on 81 children of mothers who were pregnant during the period of the Iraq poisoning. An earlier case-series reported data on 84 children (Marsh et al., 1981), however a later publication by the same authors on a slightly smaller sample of 81 mother-child pairs provided more details (Marsh et al., 1987) and has historically been used in risk assessments of methylmercury (MeHg). Maternal hair (MH) total mercury (THg) covering the period of gestation was measured in the 81 mothers as a metric of prenatal MeHg exposure. Mercury levels in maternal hair ranged from 1 to 674 µg/g THg, and therefore, the sample of 81 mother-child pairs included both mother-child pairs exposed to a wide range of exposures from low background levels (i.e. unexposed) up to >600x higher exposure. The neurological exam was conducted in the family home when the child was between ages 3 to 5 years by 2 neurologists who were blinded to the mother’s hair mercury level (Marsh et al., 1987). The neurological exam was reported to have included “observation, measurement of head circumference and body length, cranial nerve signs, speech, involuntary movements, limb tone, strength, deep tendon reflexes, plantar responses, coordination, dexterity, primitive reflexes, sensation, posture, and ability to sit, stand, walk, and run” (Marsh et al., 1987). A scoring system was adopted with composite neurological scores assigned for each child ranging from 0 (absolutely normal) to 11 (maximum severity) and the scores were dichotomized by the authors as abnormal (scores 4-11) vs. normal (scores 0-3). The raw data for neurological scores, maternal hair THg, and other mother-reported “symptoms” were extracted from Table 1 of Marsh et al. (1987) and are provided in a MS Excel worksheet in Supplemental File S6B. Data on the mother-reported “symptoms” in children were extracted in Supplemental File S6B, but were not considered for BMD modelling due to the multiple uncertainties with this data (US EPA, 2001). Only the clinician-assigned child neurological scores were considered further for BMD analysis.

A plot of the clinician-assigned child neurological scores and maternal hair THg concentrations for the 81 mother-child pairs is shown in Figure S6A.1 with colour of the points denoting the neurological scores dichotomized as reported by Marsh et al. (1987): normal score 0-3 (**blue** points) and abnormal score ≥4 (**red** points). The plot also shows five categories of exposure (grey vertical dotted lines) with geometric mean (GM) and ranges based on Table 6-3 from US EPA (1997) and Table 6-11 from NAS (1991). The plot appears to show an exposure-dependent increase in score severity for some observations with the most severe adverse neurological scores (i.e., scores of 11) associated with the highest exposure levels (404 to 443 µg/g MH THg). However, there were also a substantial proportion of children at the highest exposure levels who were assigned normal neurological scores (0-3) including the highest maternal hair THg concentration (674 µg/g) with a child neurological score of 2.

**Figure S6A.1 Child neurological scores vs maternal hair THg in the 81 mother-child pairs from Marsh et al. (1987).** Jittering has been added to the plot in order to more clearly show overlapping datapoints. The red horizontal line distinguishes scores classified by Marsh et al. as “abnormal” (**red**, scores 4-11) vs “normal” (**blue**, scores 0-3). (summary statistics and the raw data tables are also provided in MS Excel form in Supplemental File S6B.2 and S6B.3 respectively)


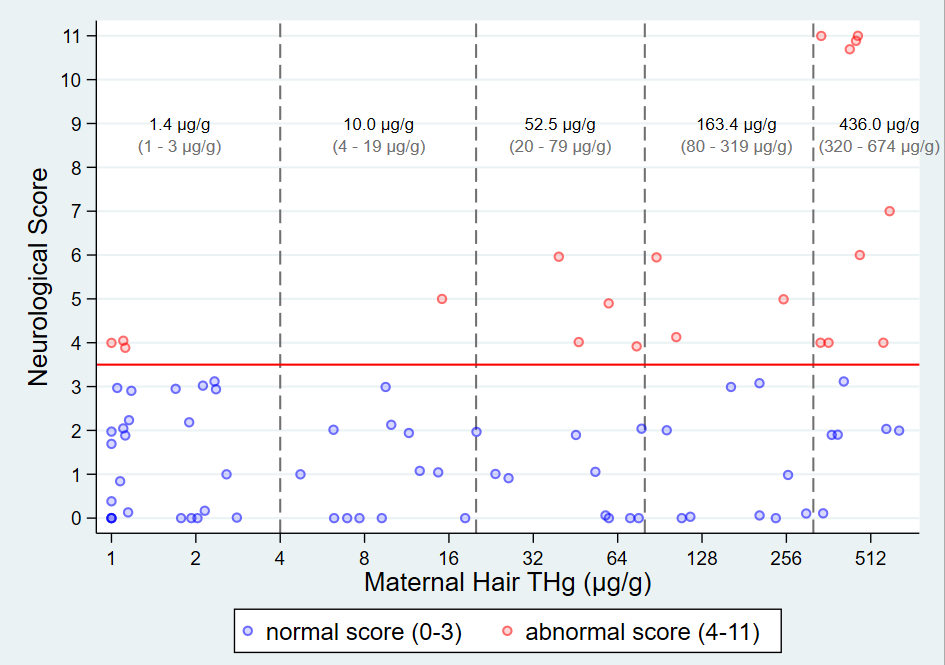


# BMD Modelling

The incidence of abnormal neurological scores by the US EPA exposure categories is shown in Table S6A.1. As in was done for the previous BMD modelling by the US EPA (US EPA, 1997) two definitions for abnormal neurological score was used, abnormal score ≥4 (4-11) as originally defined by Marsh et al. (1987), and an alternative definition of abnormal score ≥5 (5-11) from Table 6-3 from US EPA (1997) and Table 6-11 from NAS (1991).

**Table S6A.1 The incidence of abnormal neurological score by the 5 US EPA exposure categories**

(incidence data also provided in MS Excel form in Supplemental File S6B.1)

| **Hair THg** | | | **Total (n)** | **Abnormal score ≥ 4** | | **Abnormal score ≥ 5** | |
| --- | --- | --- | --- | --- | --- | --- | --- |
| **Category** | **Range (mg/g)** | **GM (mg/g)** |  | **Abnormal (n)** | **Abnormal (%)** | **Abnormal (n)** | **Abnormal (%)** |
| 1 | 1 - 3 | 1.37 | 27 | 3 | 11.11% | 0 | 0.00% |
| 2 | 4 - 19 | 10.00 | 14 | 1 | 7.14% | 1 | 7.14% |
| 3 | 20 - 79 | 52.53 | 13 | 4 | 30.77% | 2 | 15.38% |
| 4 | 80 - 319 | 163.38 | 12 | 3 | 25.00% | 2 | 16.67% |
| 5 | 320 - 674 | 436.60 | 15 | 9 | 60.00% | 6 | 40.00% |
| Totals | | | 81 | 20 | 100% | 11 | 100% |

Updated dose-response modeling of the incidence data from Table S6A.1 was performed using the US EPA’s online Benchmark Dose (BMD) Software which employs a Bayesian model-average method (US EPA, n.d.). Quantal models with a BMR of 5% extra-risk was used instead of the default of 10% extra-risk typically recommended for BMD of binary outcomes in order to align with previous BMD model estimates for MeHg in Tables 2^[[1]](#footnote-2)^. The background probability of abnormal P(0) was estimated in the models. The Bayesian model-average BMDs and BMDLs reported from the US EPA BMDS are shown in Table S6A.2. Since the US EPA does not have guidance for acceptability of their updated Bayesian BMD estimates, criteria for assessing Bayesian BMD estimates from the updated EFSA BMD guidance was used (EFSA, 2022).

**Table S6A.2 Bayesian Model Average BMD and BMDL estimates (5% extra-risk) from US EPA BMDS**

Detailed BMDS output are provided in Appendix S6A.A (for abnormal score ≥4) and Appendix S6A.B (for abnormal score ≥5).

| **Abnormal Neurological Score Definition** | **BMDL**  **(mg/g hair THg)** | **BMD**  **(mg/g hair THg)** | **BMDU**  **(mg/g hair THg)** | **BMD/BMDL ratio** | | **BMDU/BMDL**  **ratio** | |
| --- | --- | --- | --- | --- | --- | --- | --- |
|  |  |  |  | **ratio** | **EFSA Guidance (flag if ratio>20)** | **ratio** | **EFSA Guidance**  **(flag if ratio>50)** |
| ≥ 4 | 12.9 | 47.2 | 139.7 | 3.7 | no flag | 10.8 | no flag |
| ≥ 5 | 17.5 | 68.6 | 185.9 | 3.9 | no flag | 10.6 | no flag |

**References**

EFSA. (2022). *Guidance on the use of the benchmark dose approach in risk assessment. (Published 25 October 2022)*. https://efsa.onlinelibrary.wiley.com/doi/epdf/10.2903/j.efsa.2022.7584

Marsh, D. O., Clarkson, T. W., Cox, C., Myers, G. J., Amin Zaki, L., & Tikriti, S. (1987). Fetal methylmercury poisoning: Relationship between concentration in single strands of maternal hair and child effects. *Archives of Neurology*, *44*(10), 1017–1022. https://doi.org/10.1001/archneur.1987.00520220023010

Marsh, D. O., Myers, G. J., Clarkson, T. W., Amin-Zaki, L., Tikriti, S., Majeed, M. A., & Dabbagh, A. R. (1981). Dose-response relationship for human fetal exposure to methylmercury. *Clinical Toxicology*, *18*(11), 1311–1318. https://doi.org/10.3109/00099308109035071

NAS. (1991). *Methylmercury (p196-219). Chapter 6. Critique of Risk Assessments Used in Formulating Contaminant Guidelines/Tolerances for Specific Chemicals and Suggestions for Improvement. In: Seafood Safety. Committee on Evaluation of the Safety of Fishery Products.* . The National Academies Press. https://doi.org/10.17226/1612

US EPA. (n.d.). *Benchmark Dose Modeling Software (BMDS Online)*. https://bmdsonline.epa.gov/

US EPA. (1997). Volume V: Health Effects of Mercury and Mercury Compounds. Mercury Study Report to Congress. December 1997 (EPA-452/R-97-003) Office of Air Quality Planning and Standards, Office of Research and Development, U.S. Environmental Protection Agency. In *Mercury Study Report to Congress*. https://www.epa.gov/mercury/mercury-study-report-congress

US EPA. (2001). *Water Quality Criterion for the Protection of Human Health: Methylmercury. Final. (January 2001, EPA 823-R-01-001). Office of Science and Technology Office of Water U.S. Environmental Protection Agency Washington, DC 20460*. https://doi.org/https://www.epa.gov/sites/default/files/2020-01/documents/methylmercury-criterion-2001.pdf

**Appendix S6A.A US EPA Bayesian Model Average BMDS Output for Abnormal Neurological Score ≥4**

**Report Generated:** 2025-Mar-24 01:03 UTC

**BMDS Online Version:** 24.1 (pybmds 24.1; bmdscore 24.1)

**Session for outcome: abnormal score >=4**

Dataset

**Name:** outcome: abnormal score >=4

| Dose (ug/g hair THg) | N | Incidence |
| --- | --- | --- |
| 1.37 | 27 | 3 |
| 10 | 14 | 1 |
| 52.53 | 13 | 4 |
| 163.38 | 12 | 3 |
| 436.6 | 15 | 9 |

Settings

| Setting | Value |
| --- | --- |
| BMR | 5% Extra Risk |
| Confidence Level (one sided) | 0.95 |
| Maximum Multistage Degree | 2 |
| Samples | 100 |
| Burn-in | 20 |

Bayesian Summary

| Model | Prior Weights | Posterior Weights | BMDL | BMD | BMDU | Unnormalized Log Posterior Probability | Scaled Residual near BMD | Scaled Residual at Control |
| --- | --- | --- | --- | --- | --- | --- | --- | --- |
| Hill | 0.111 | 0.05 | 5.377 | 41.621 | 168.125 | -48.766 | -0.201 | 1.173 |
| Gamma | 0.111 | 0.056 | 11.882 | 50.27 | 139.793 | -36.618 | -0.368 | 1.194 |
| Logistic | 0.111 | 0.092 | 44.482 | 65.686 | 123.339 | -37.605 | -0.589 | 1.148 |
| LogLogistic | 0.111 | 0.11 | 6.891 | 47.976 | 170.254 | -43.394 | -0.301 | 1.208 |
| LogProbit | 0.111 | 0.054 | 23.477 | 129.077 | 270.707 | -43.418 | -0.653 | 0.107 |
| Multistage 2 | 0.111 | 0.071 | 22.88 | 48.821 | 108.098 | -41.04 | -0.33 | 1.217 |
| Probit | 0.111 | 0.144 | 42.367 | 61.602 | 108.357 | -35.022 | -0.486 | 1.208 |
| Quantal Linear | 0.111 | 0.357 | 19.069 | 32.848 | 70.62 | -35.142 | -0.241 | 1.041 |
| Weibull | 0.111 | 0.066 | 4.717 | 40.794 | 158.145 | -42.31 | -0.313 | 1.116 |
| Model Average | - | - | 12.863 | 47.192 | 139.746 | - | - | - |


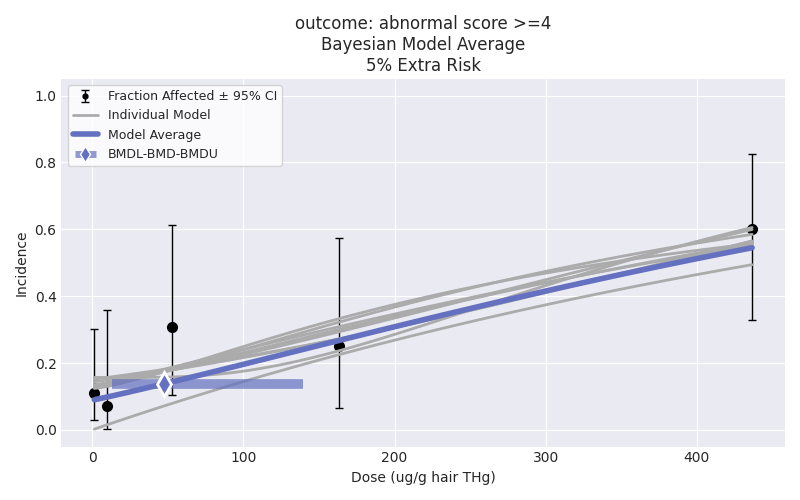


**Appendix S6A.5 US EPA Bayesian Model Average BMDS Output for Abnormal Neurological Score ≥5**

**Report Generated:** 2025-Mar-24 02:03 UTC

**BMDS Online Version:** 24.1 (pybmds 24.1; bmdscore 24.1)

Session for outcome: abnormal score >=5

Dataset

**Name:** outcome: abnormal score >=5

| Dose (ug/g hair THg) | N | Incidence |
| --- | --- | --- |
| 1.37 | 27 | 0 |
| 10 | 14 | 1 |
| 52.53 | 13 | 2 |
| 163.38 | 12 | 2 |
| 436.6 | 15 | 6 |

Settings

| Setting | Value |
| --- | --- |
| BMR | 5% Extra Risk |
| Confidence Level (one sided) | 0.95 |
| Maximum Multistage Degree | 2 |
| Samples | 100 |
| Burn-in | 20 |

## Bayesian Summary

| Model | Prior Weights | Posterior Weights | BMDL | BMD | BMDU | Unnormalized Log Posterior Probability | Scaled Residual near BMD | Scaled Residual at Control |
| --- | --- | --- | --- | --- | --- | --- | --- | --- |
| Hill | 0.111 | 0.086 | 8.314 | 52.963 | 199.666 | -36.668 | -1.095 | 0.814 |
| Gamma | 0.111 | 0.061 | 20.882 | 73.875 | 198.193 | -24.567 | -1.243 | 0.888 |
| Logistic | 0.111 | 0.074 | 77.382 | 116.739 | 266.819 | -26.278 | -1.474 | 0.214 |
| LogLogistic | 0.111 | 0.104 | 11.536 | 60.111 | 188.852 | -31.957 | -1.17 | 0.835 |
| LogProbit | 0.111 | 0.038 | 33.659 | 148.863 | 302.865 | -31.999 | -1.423 | 0.391 |
| Multistage 2 | 0.111 | 0.03 | 33.029 | 66.546 | 133.592 | -28.95 | -1.19 | 0.875 |
| Probit | 0.111 | 0.165 | 72.37 | 108.83 | 197.75 | -23.16 | -1.343 | 0.262 |
| Quantal Linear | 0.111 | 0.371 | 27.904 | 49.098 | 106.801 | -23.125 | -1.151 | 0.702 |
| Weibull | 0.111 | 0.071 | 9.497 | 55.043 | 193.518 | -30.072 | -1.186 | 0.742 |
| Model Average | - | - | 17.548 | 68.649 | 185.86 | - | - | - |


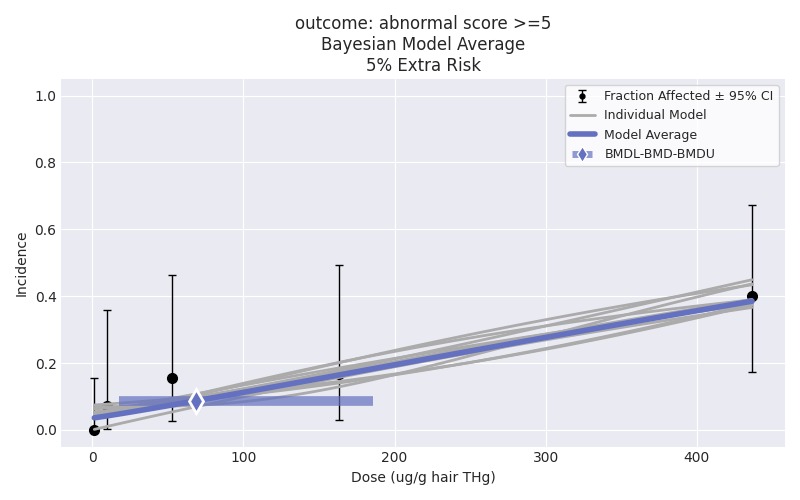


1. The BMRs in Table 2 were for a binary BMR of 5% added-risk and not % extra-risk as modelled for the Iraqi data [↑](#footnote-ref-2)
